# Structural basis of an endoplasmic reticulum EMC:Spf1 insertase–dislocase complex

**DOI:** 10.1038/s41594-026-01883-8

**Published:** 2026-10-13

**Authors:** Carolin J. Klose, J. Rajan Prabu, Emma J. Fenech, Bastian Bräuning, Ida Baydar, Barbara Steigenberger, Susanne von Gronau, Sivan Arad, Christine R. Langlois, Maya Schuldiner, Brenda A. Schulman, Matthias J. Feige

**Affiliations:** 1 Center for Functional Protein Assemblies (CPA), Department of Bioscience, TUM School of Natural Sciences, Technical University of Munich, Garching, Germany; 2 Department of Molecular Machines and Signaling, Max Planck Institute of Biochemistry, Martinsried, Germany; 3 Department of Molecular Genetics, Weizmann Institute of Science, Rehovot, Israel; 4 Center for Biochemistry and Center for Molecular Medicine Cologne, Faculty of Medicine, University of Cologne, Cologne, Germany; 5 Mass Spectrometry Core Facility, Max Planck Institute of Biochemistry, Martinsried, Germany; 6 Present Address: Scala Biodesign, Tel Aviv, Israel

**Keywords:** Cryoelectron microscopy, Endoplasmic reticulum, Membrane proteins, Multienzyme complexes

## Abstract

Most eukaryotic membrane proteins are inserted into the membrane at the endoplasmic reticulum (ER). This essential process relies on molecular quality control machineries to prevent mistargeting and incorrect structure formation. Here, we show that the ER membrane protein complex (EMC) forms an evolutionarily conserved supercomplex with the P5A-ATPase Spf1/ATP13A1. Biochemical, structural and cellular analyses demonstrate that the EMC and Spf1 associate in a stoichiometric manner and retain their individual catalytic activities, the transmembrane domain (TMD) insertase function of the EMC and the TMD dislocase activity of Spf1. Our cryo-electron microscopy structures show that the EMC insertase cavity and the Spf1 dislocase cleft are positioned in close proximity within the membrane and reveal that the ATPase cycle of Spf1 remodels the interface between the two machineries and alters cytoplasmic accessibility of both substrate-binding cavities. Together, our study provides structural insights into how membrane protein insertion and extraction machineries can be spatially coordinated at the ER membrane, suggesting that functionally distinct proteostasis factors can assemble into hubs to support membrane protein biogenesis and quality control.

## Abstract (web summary)

Klose et al. provide structural insights into a supercomplex between the EMC and Spf1 that brings together cellular machines for membrane protein insertion and extraction into a single entity, providing a framework for integrated membrane protein quality control.

## Main

Integral membrane proteins account for about one quarter of all eukaryotic genes^1,2^. They mediate essential processes in cells, including signal transduction, organelle tethering, lipid homeostasis and the transport of ions, nutrients and entire proteins. The biogenesis of membrane proteins is a complex process that relies on correct protein targeting, insertion into the lipid bilayer and structure formation within and outside the membrane. Thus, eukaryotic cells have evolved multiple complementary systems to safeguard targeting and insertion of membrane proteins into the lipid bilayer. For the secretory pathway, which acts as a hub for lipid bilayer integration and structure formation of most membrane proteins, targeting mechanisms include the signal recognition particle (SRP) pathway^1,3^, the guided entry of tail-anchored (TA) proteins (GET) system^4^ and the SRP-independent targeting pathway^5^. Downstream of the SRP pathway, the Sec61 translocon was initially identified as the principal conduit for cotranslational insertion of nascent polypeptides into the endoplasmic reticulum (ER) membrane^6–8^. In addition to Sec61, a growing number of specialized machineries have been discovered that mediate the insertion and folding of structurally diverse membrane protein clients. Recent findings show that these machineries can assemble into large multisubunit complexes, such as the multipass translocon^9^ and the holo-insertase complex^10^, to accommodate and coordinate membrane protein insertion and quality control for complex clients.

A key machinery for membrane protein biogenesis is the ER membrane protein complex (EMC). The EMC has emerged as a central player in both membrane protein biogenesis and cellular homeostasis^10–13^. The EMC is an evolutionarily conserved complex in eukaryotes, comprising eight subunits in yeast and nine in humans^14^. Cryo-electron microscopy (cryo-EM) structures of the human and yeast EMC^15–18^ reveal a sophisticated, three-layered architecture: the EMC subunits 1, 7 and 10 form a characteristic ER lumenal domain, the transmembrane core is composed of the subunits 3–6 and the cytoplasmic cap is formed by Emc2, Emc5 and in humans either Emc8 or Emc9. Central to the EMC’s function is its ability to insert transmembrane domains (TMDs) into the ER membrane, particularly terminal helices and those with moderate hydrophobicity^12,13^. This function has mostly been tied to the subunit Emc3, a member of the YidC/Oxa1 superfamily^19^. Together with the subunits Emc4 and Emc6, Emc3 forms an intramembrane groove, which was assigned this insertase function^20,21^. Beyond its insertase activity, the EMC has been shown to act as a chaperone for membrane proteins^22–25^. Furthermore, the EMC interacts with multiple machineries involved in membrane protein biogenesis and quality control, including the back of Sec61 (BOS) and PAT complexes^10^, the signal peptidase complex, the GET system or the oligosaccharyltransferase (OST) complex^24^, as well as components of the ER-associated degradation (ERAD) machinery^26^. These observations position the EMC as a central node within the ER membrane proteostasis network.

While organellar membrane protein insertases generally display preferences for certain clients, they often must also allow for promiscuous insertions to accommodate a spectrum of client properties. As a consequence, insertion is inherently error-prone and requires downstream surveillance and quality control mechanisms. In mitochondria, for example, mistargeted TA proteins are extracted from the outer membrane by the AAA-ATPase Msp1 to be degraded at the ER^27,28^. Similarly, the EMC may erroneously integrate mitochondrial TA proteins into the ER membrane^21,29–31^. Here, the P5A-ATPase Spf1 (yeast)/ATP13A1 (human) functions as a dislocase, surveilling the integration of TA proteins, as well as erroneously integrated signal sequences and TMDs^32^. This extraction activity maintains organellar integrity and can give transmembrane and signal sequence-containing proteins another chance to obtain their destined localization and native structure^31,33^. The dislocase function of Spf1/ATP13A1 is driven by its ATPase cycle, referred to as the Post–Albers cycle^34,35^. Recent cryo-EM structures of Spf1 bound to different nucleotide analogs and engaged with endogenous substrate have provided detailed insights into Spf1’s functional role and mechanisms of its catalytic cycle^32,36^. For substrate sampling and selection, Spf1 houses an intramembrane cleft with a negatively charged patch at its ER lumenal end^32^. While most P-type ATPases transport cargo from the cytosol into the ER lumen or extracellular space, Spf1 operates in the opposite direction, extracting its polypeptide cargo from the ER membrane to the cytosol^32,36^.

By acting together, insertases, chaperones and dislocases support the fidelity of organelle-specific membrane protein targeting and biogenesis. How membrane protein insertion and quality control machineries are coordinated within the ER membrane remains poorly understood. Given EMC’s central role in this network, we aimed to identify regulators and interactors that might contribute to such coordination. Our data reveal that the transmembrane helix dislocase Spf1/ATP13A1 is an endogenous stoichiometric interactor of the EMC in both yeast and human cells. Cryo-EM structures of the EMC:Spf1 complex reveal a close juxtaposition of the EMC insertase and Spf1 dislocase cavities while preserving the structural and functional integrity of both machineries. These findings provide structural insight into how membrane protein biogenesis and quality control machineries can assemble into higher-order membrane proteostasis hubs that spatially coordinate insertion and extraction activities within the ER membrane as crucial events during membrane protein biogenesis.

## Results

### The EMC forms a stoichiometric complex with the dislocase Spf1 in the ER membrane

**Fig. 1:**
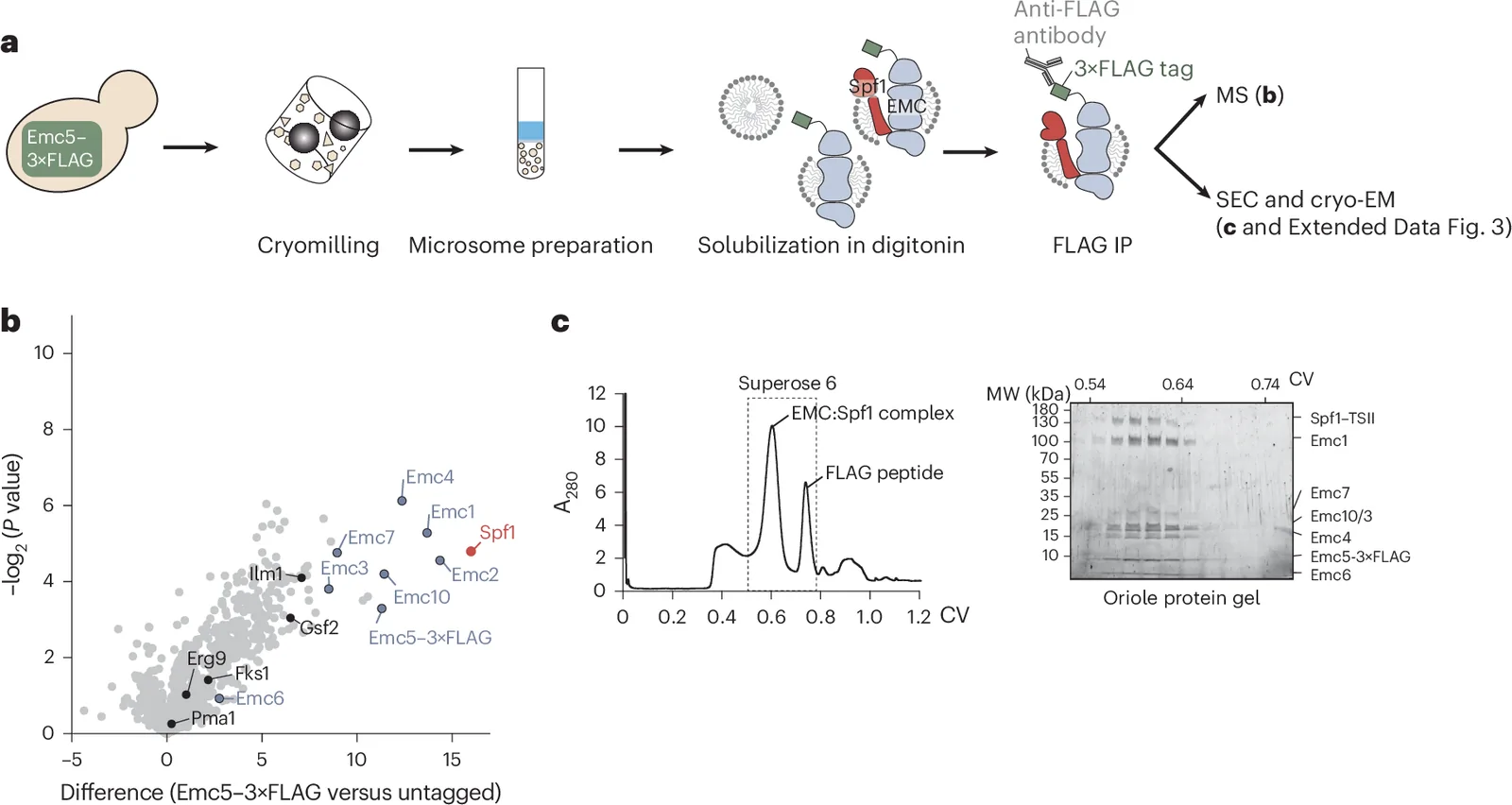
EMC and Spf1 form a stoichiometric complex. **a**, Procedure for mild isolation of endogenously tagged Emc5–3×FLAG from *S*. *cerevisiae* by cryogenic disruption of the cell wall, centrifugal isolation of microsomes and solubilization in digitonin before immunocapture of the 3×FLAG tag. **b**, The interactome of Emc5–3×FLAG reveals Spf1 (red) as the most prevalent interaction partner of the EMC in yeast. EMC subunits are labeled in light blue and previously identified interactors^22^ are labeled in black. A two-tailed Student’s *t*-test was applied to compare the interactome of Emc5 to values measured for an untagged strain. **c**, In a large-scale purification of the endogenous EMC, Spf1 coelutes in a stoichiometric manner after a single pulldown on Emc5 and SEC. The figure shows the ultraviolet absorption trace at 280 nm of the SEC and SDS–PAGE analysis of the indicated fractions (dashed box) stained for whole protein. The complete purification is shown in Extended Data Fig. 1d. Source data

**Extended Data Fig. 1:**
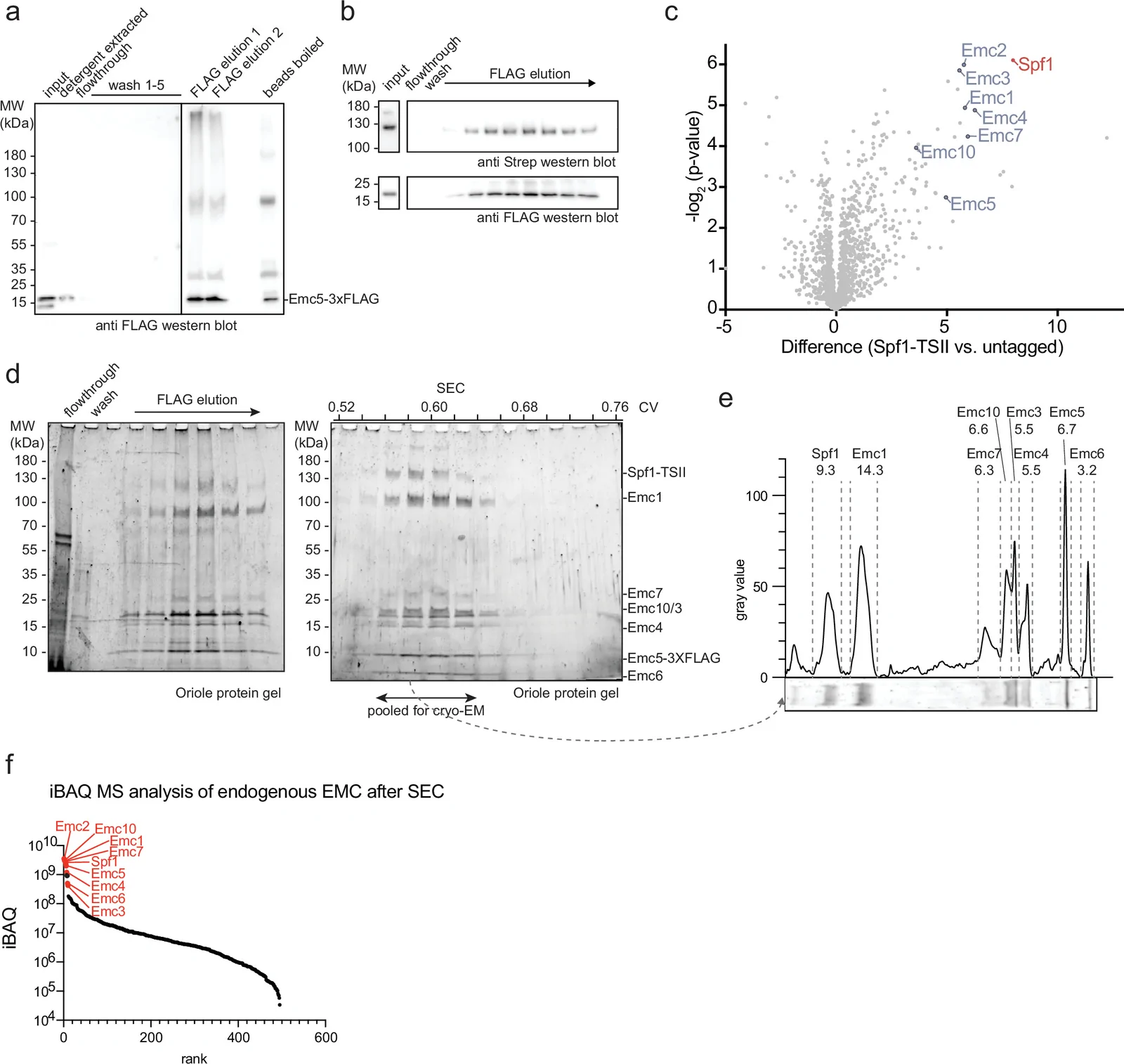
Validation of the EMC:Spf1 interaction and its stoichiometry at endogenous level in *S. cerevisiae*. **a**, Anti FLAG western blot showing the different steps of the isolation procedure of Emc5-3XFLAG from *S. cerevisiae*. The experiment was performed in independent triplicates, one representative result is shown. **b**, Co-immunoprecipitation of endogenously tagged Spf1-TSII with Emc5-3XFLAG confirms the interaction at endogenous levels. The experiment was performed in independent triplicates, one representative result is shown. **c**, The interactome of endogenously TSII-tagged Spf1 validates the interaction with the EMC from the Spf1 side. A two-tailed unpaired Student’s t-test was applied to compare the interactome of Spf1 with values measured for an untagged strain. **d**, SDS-PAGE protein gels stained with Oriole stain of the large-scale purification of endogenous EMC from a 400 g yeast pellet by an anti-FLAG pulldown and size exclusion chromatography. The second gel is also shown in Fig. 1c and has been reproduced here for completeness. **e**, Quantification of protein band intensities using densitometry of lane 4 of the SEC gel shown in d. The respective region was cropped and rotated by 90°, corrected for background signal and intensities were quantified. Individual peaks corresponding to Spf1 and the EMC subunits were marked and labeled with the integrated area under the curve. **f**, iBAQ MS analysis of the endogenously isolated EMC validates Spf1 as most abundant interaction partner among the EMC subunits. Source data

The multisubunit architecture of the EMC and recent studies on this complex^10,22,24,26^ indicate that it operates within a network of membrane protein biogenesis and quality control factors, rather than as an isolated standalone molecular machine. To gain a comprehensive view of the endogenous cofactors and modulators that associate with the EMC, we tagged one of its core subunits (Emc5) in *Saccharomyces* *cerevisiae* (from here on called yeast) with a C-terminal triple FLAG tag and performed immunoprecipitation coupled to mass spectrometry (IP–MS) to define the EMC interactome. We used a previously established mild isolation strategy^22^, using the nonionic detergent digitonin to also preserve weak interactions (Fig. 1a and Extended Data Fig. 1a). This approach identified approximately 250 candidate interactors of the yeast EMC, including previously characterized ones such as the chaperone Ilm1 (ref. ^22^), as well as proteins involved in ER targeting and insertion, the OST complex, ERAD-related proteins, ER-shaping proteins and numerous factors involved in lipid and sterol metabolism (Fig. 1b, Supplementary Table 1 and Supplementary Fig. 1a,b). Among the most strongly enriched factors was the P5A-type ATPase Spf1, with an enrichment level comparable to the EMC subunits themselves (Fig. 1b), in agreement with a previous interactomics study reporting a prominent EMC:Spf1 association^22^.

This interaction may appear unexpected, as the function of Spf1 as a transmembrane helix dislocase in the ER membrane^32^ is biochemically opposite to the role of the EMC as an insertase^13^. However, both factors contribute to membrane protein quality control, raising the possibility that their activities may be functionally coordinated. Supporting this notion, similar phenotypes after knockout (KO) of either have been described, related to defects in sterol metabolism^37–39^. Furthermore, recent studies discussed an overlap in client spectra between the human orthologs hEMC and ATP13A1 (refs. ^31,33,40^), also indicating potential connections between the two systems. To further validate this interaction, we endogenously tagged Spf1 with a TwinStrep II (TSII) tag and confirmed its presence in an anti-Emc5–FLAG IP by western blotting (Extended Data Fig. 1b). Additionally, we performed the reciprocal IP–MS experiment on endogenously tagged Spf1 and found the EMC subunits to be amongst the most highly enriched interactors (Extended Data Fig. 1c, Supplementary Table 1 and Supplementary Fig. 1a,b).

To characterize this EMC:Spf1 interaction, we purified the endogenous EMC from yeast membranes in digitonin at a preparative scale. Following affinity purification of Emc5 and subsequent size-exclusion chromatography (SEC), Spf1 coeluted with the EMC at levels comparable to the EMC subunits (Fig. 1c and Extended Data Fig. 1d,e). These results demonstrate that EMC and Spf1 form a stable endogenous complex, providing biochemical evidence for a stoichiometric association between these insertase and dislocase machineries.

### The cryo-EM structure of the EMC:Spf1 complex reveals juxtaposed insertase and dislocase sites

**Fig. 2:**
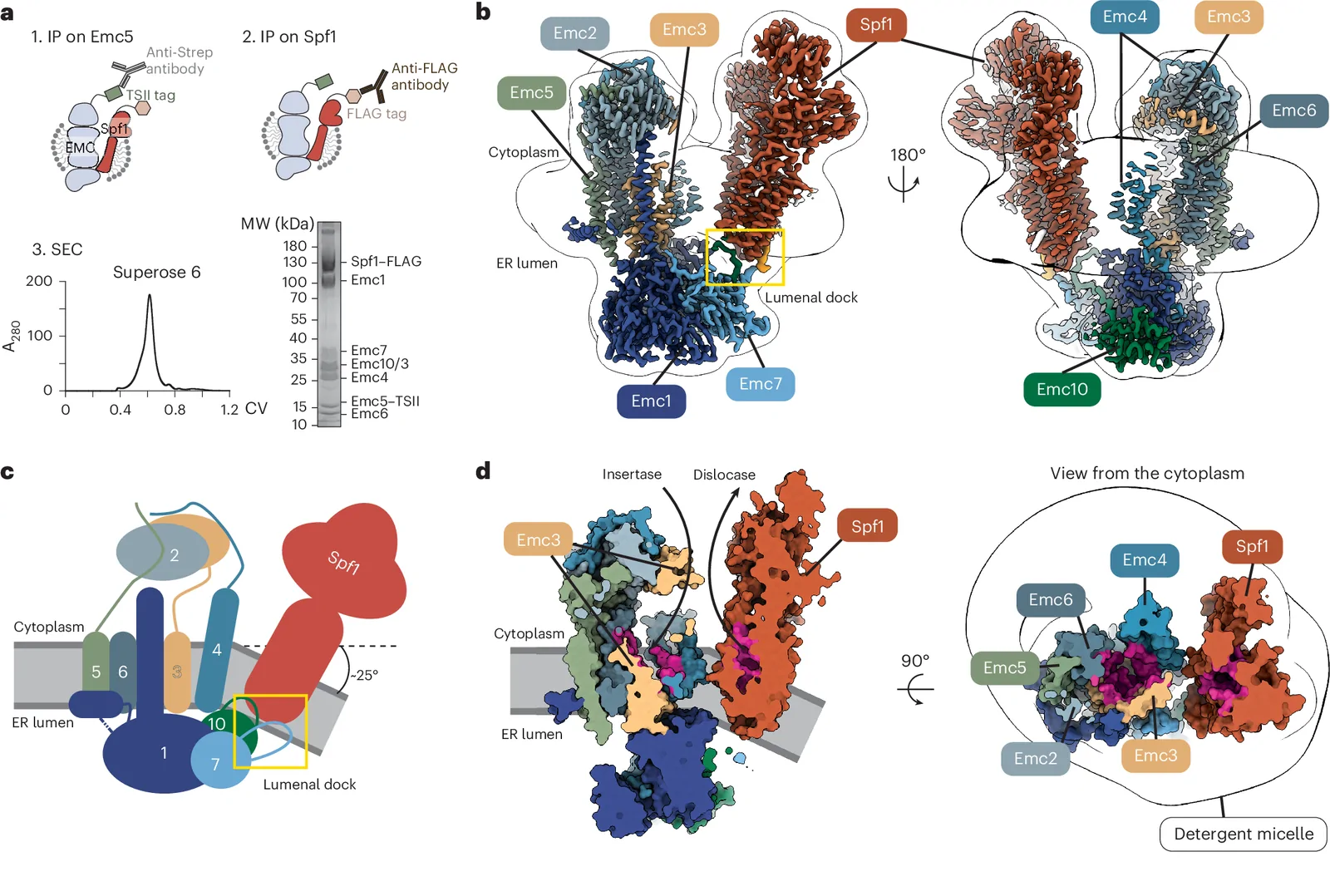
The cryo-EM structure of the EMC:Spf1 complex reveals a large composite intramembrane cavity encompassing both client helix engagement sites. **a**, Recombinant expression and purification of the EMC:Spf1 complex in insect cells using a tandem affinity pulldown and SEC, yielding a homogeneous sample for cryo-EM, as shown on the Coomassie-stained SDS–PAGE gel. **b**, Cryo-EM map of the EMC:Spf1 complex colored according to subunit identity. The outline of a lowpass-filtered map is overlaid to show the borders of the detergent micelle. The main interaction interface between the EMC and Spf1, the lumenal dock, is marked with a yellow box. **c**, Model cartoon depicting the subunit architecture of the EMC:Spf1 complex and highlighting the bend of approximately 25° in the detergent micelle and the lumenal dock. **d**, The insertase site of the EMC is facing the dislocase site of Spf1 within the intramembrane space. The view from the membrane (left) and top view from the cytoplasm (right) show the size and continuity of the composite cavity, highlighted in magenta. Source data

**Extended Data Fig. 2:**
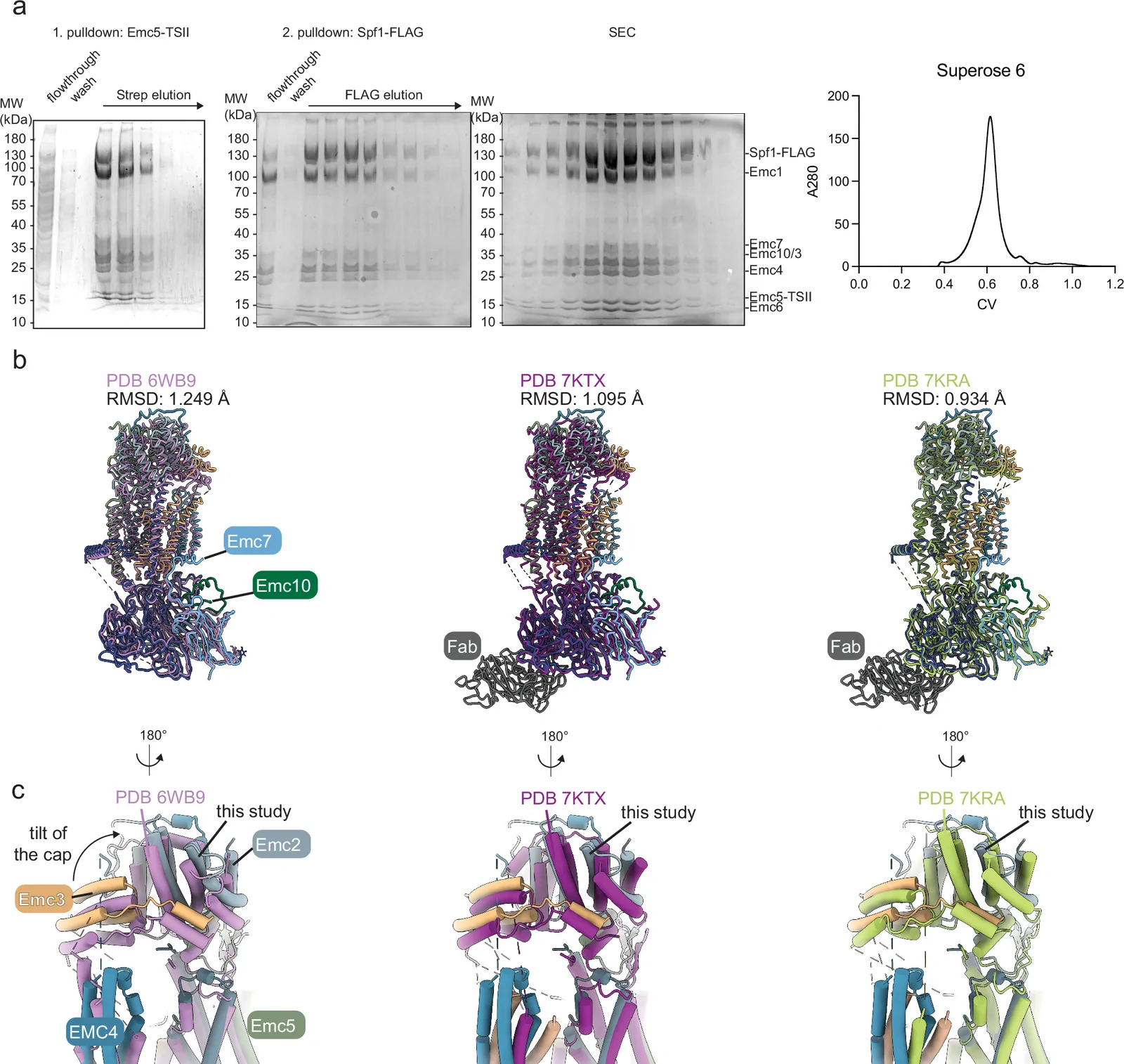
Purification of the recombinant EMC:Spf1 complex and comparison of the EMC to previously published structures. **a**, Purification of the EMC:Spf1 complex from insect cells via a tandem pulldown, first on Emc5-TSII, second on Spf1-FLAG followed by size exclusion chromatography on a Superose 6 column. The chromatogram and a lane of the SEC gel are also shown in Fig. 2a and have been reproduced here for completeness. **b**, Comparison of the EMC structure in complex with Spf1 to previously reported structures of the yeast EMC (PDBs: 6WB9, 7KTX, 7KRA). **c**, View of the cytoplasmic cap of the EMC, which is shifted in the EMC:Spf1 complex in comparison to apo EMC. The arrow indicates the direction of the cap tilt. Source data

**Extended Data Fig. 3:**
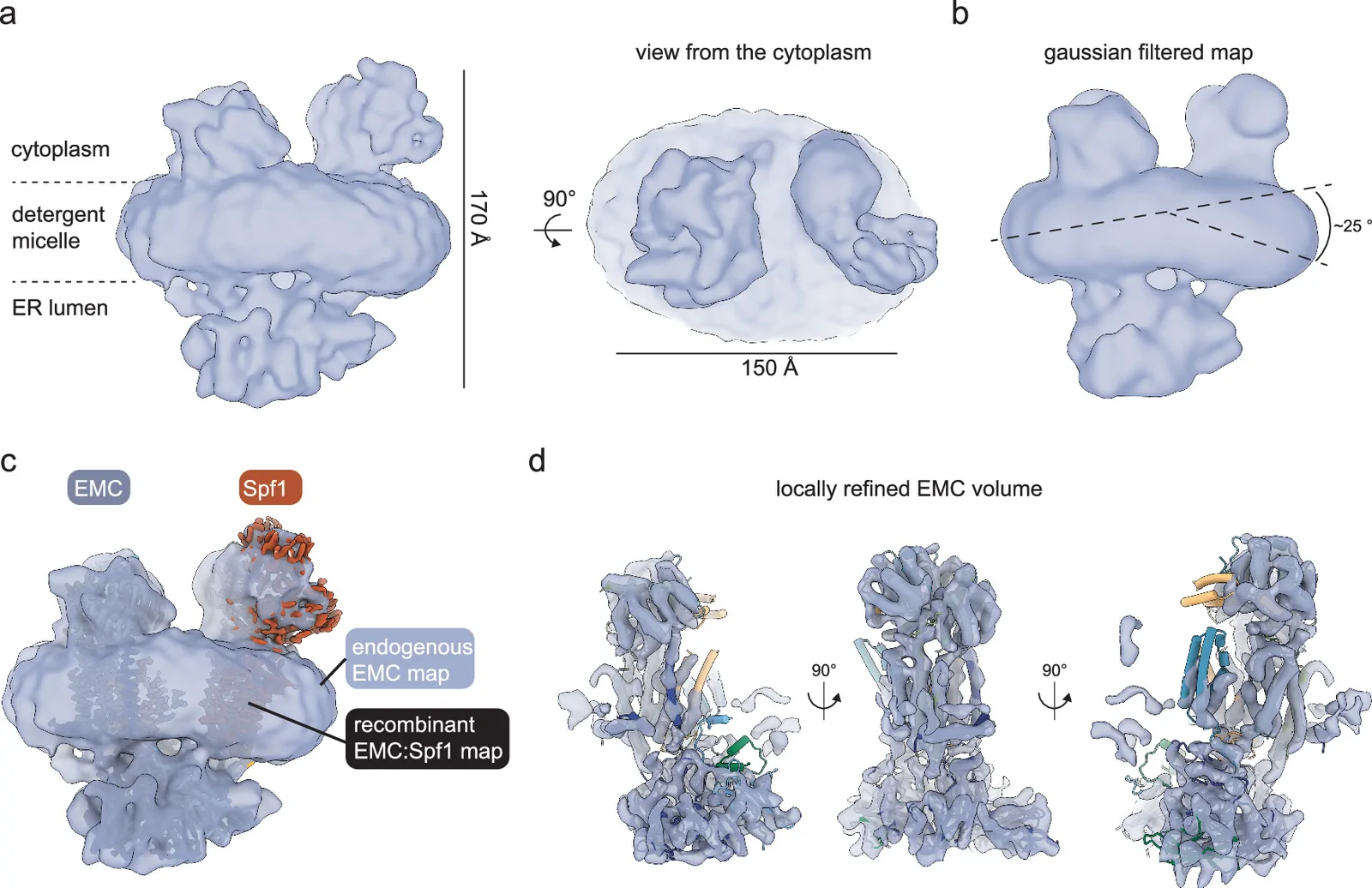
Cryo-EM map of the endogenous EMC:Spf1 from yeast. **a**, Reconstruction of the endogenous EMC:Spf1 volume after 3D flexible refinement, showing an EMC-like density (left) and an additional density (right) that likely corresponds to Spf1. **b**, The endogenous EMC:Spf1 complex introduces a bend in the detergent micelle to a similar extent as the recombinant complex. **c**, Overlay of the maps of the EMC obtained from endogenous sources (grey) and the side-chain resolution map of the recombinantly expressed EMC:Spf1 supercomplex (color coded according to subunit identity) showing that the recombinant structure is fully consistent with the endogenous map. **d**, Local refinement of the EMC component of the endogenous map shows secondary structure elements and consistency with the structural model derived from the recombinant EMC:Spf1 complex which is docked into the density here.

To gain insights into the molecular details of the EMC:Spf1 complex, we obtained large homogeneous amounts of the yeast complex by recombinant production in insect cells and a tandem affinity purification followed by SEC (Fig. 2a and Extended Data Fig. 2a). This allowed us to determine the structure of the complex by cryo-EM to a global resolution of 3.8 Å for the EMC and 4.2 Å for Spf1 (Fig. 2b, Supplementary Fig. 2 and Table 1). To verify that this architecture reflects the endogenous complex, we additionally purified EMC:Spf1 directly from yeast through endogenously tagged Emc5 and obtained a low-resolution cryo-EM map (global resolution: 8.7 Å). The recombinant complex is fully consistent with this endogenous assembly (Extended Data Fig. 3, Supplementary Fig. 3 and Table 1). To additionally assess whether the complex remains intact in a membrane-like environment, we reconstituted the recombinantly expressed, purified EMC:Spf1 complex into lipid nanodiscs, using an MSP2N2 scaffold and a yeast lipid mixture (Supplementary Fig. 4). The complex remained stable upon reconstitution, providing independent biochemical evidence that the EMC:Spf1 interaction persists following detergent removal and nanodisc reconstitution.

**Table 1:** Cryo-EM data collection, reconstruction and model refinement statistics

	EMC:Spf1 (apo) in digitonin (EMD-59745; PDB 34BQ): Spf1 component (EMD-59736), EMC component (EMD-59737)	Endogenous EMC from *S. cerevisiae* bound to Spf1 in digitonin (EMD-54652)	EMC:Spf1 in E1-ATP state in digitonin (EMD-59746; PDB 34BR)	EMC:Spf1 in E1-P state in digitonin (EMD-54635)
**Data collection and processing**				
Magnification	105,000	22,000	105,000	22,000
Voltage (kV)	300	200	300	200
Electron exposure (e^−^ per Å^2^)	65.1	60.0	62.6	60.0
Defocus range (μm)	−0.7 to −2.2	−1 to −2.6	−0.7 to −2.2	−1 to −2.6
Pixel size (Å)	0.8222	1.885	0.8222	1.871
Symmetry imposed	*C*_1_	*C*_1_	*C*_1_	*C*_1_
Initial particle images (no.)	3,817,633	521,753	2,275,267	2,059,914
Final particle images (no.)	645,091	80,281	33,661	103,225
Map resolution (Å)	4.2	8.7	3.8	6.8
FSC threshold	0.143	0.143	0.143	0.143
Map resolution range (Å)				
**Refinement**				
Initial model used (PDB code)	AlphaFold 2 7KRA		AlphaFold 2 7KRA	
Model resolution (Å)	4.2		3.8	
FSC threshold	0.143		0.143	
Model resolution range (Å)	3.2–12.6		3.2–9.9	
Map sharpening *B* factor (Å^2^)	-		-	
Model–map correlation (box)	0.74		0.70	
Model composition				
Nonhydrogen atoms	23,389		24,982	
Protein residues	2,968		3,115	
Ligands	8		8	
*B* factors (Å^2^)				
Protein	45.0		87.57	
Ligand	38.73		87.18	
R.m.s.d.				
Bond lengths (Å)	0.003		0.003	
Bond angles (°)	0.613		0.649	
Validation				
MolProbity score	1.8		1.93	
Clashscore	7.91		12.66	
Poor rotamers (%)	0		0.98	
Ramachandran plot				
Favored (%)	94.64		95.46	
Allowed (%)	5.32		4.54	
Disallowed (%)	0.03		0.00	

FSC, Fourier shell correlation.

In the EMC:Spf1 complex, all EMC subunits (Emc1–Emc7 and Emc10) adopt an overall arrangement similar to previously reported structures of the yeast EMC (Extended Data Fig. 2b; root-mean-square deviation (r.m.s.d.) to PDB 7KRA: 0.9 Å, r.m.s.d. to PDB 7KTX: 1.1 Å, r.m.s.d. to PDB 6WB9: 1.2 Å)^15,18^. Extending this former work, we resolved previously unmodeled C-terminal residues for the subunits Emc4, Emc7 (also named Sop4) and Emc10 (Supplementary Fig. 5)^15,18^. Compared to the prior structures of isolated EMC complexes, we observed a slight conformational change in Emc3, the subunit that is homologous to the evolutionarily conserved Oxa1/Alb3/YidC family of insertases^19^. In our structure, the cytoplasmic helices of Emc3 were shifted away from its transmembrane part, thereby increasing access from the cytosol to the hydrophilic groove for substrate insertion (Extended Data Fig. 2c).

**Extended Data Fig. 4:**
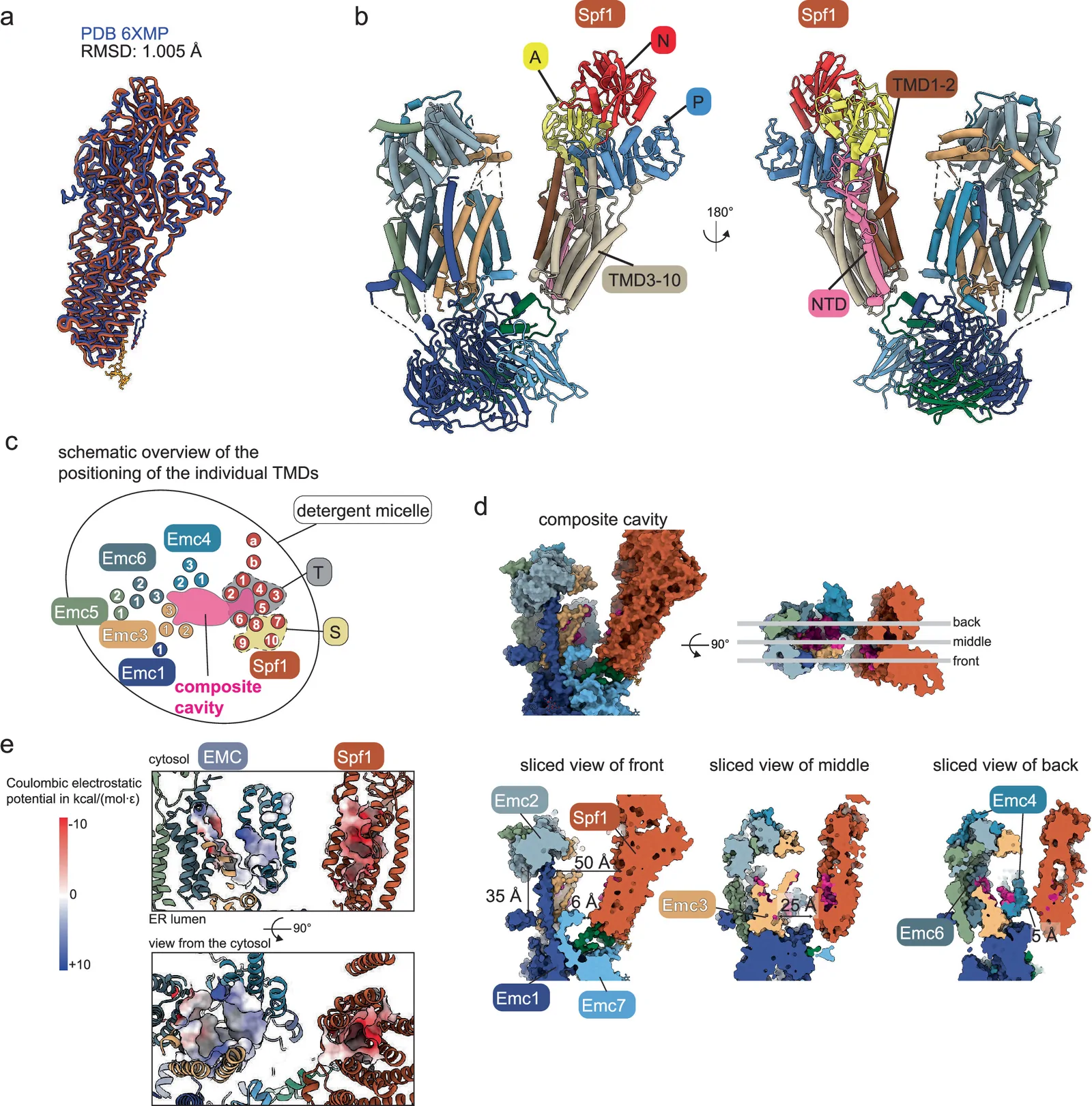
Architecture of Spf1 and the composite intramembrane cavity. **a**, Comparison of the Spf1 structure in complex with EMC to a previously reported structure of apo Spf1 (PDB 6XMP). **b**, Model of the EMC:Spf1 complex color coded according to Spf1 domain identity, featuring the actuator (A), phosphorylation (P), nucleotide binding (N) domains as well as the N-terminal domain (NTD). The substrate binding cleft is located between transmembrane domains colored in brown (TMD1-2) and in sand (TMD3-10). Arrangement of the transport (T; TMD1-6) and support domain (S; TMD7-10) is outlined in panel **c**. **c**, Schematic overview of the arrangement of the TMDs of the EMC and Spf1 in the detergent micelle and highlighting the area of the composite intramembrane cavity enclosed by these two protein complexes and the architecture of Spf1’s TMDs arranged into the transport (T) and support (S) domains. **d**, Detailed views of the composite cavity enclosed by the EMC and Spf1. The model of the EMC:Spf1 complex is shown in surface representation from the membrane (left) and the top (right). In the top view the planes are indicated where the sections displayed below (front, middle, back) were taken. Residues lining the insertase and dislocase cavities are highlighted in magenta. **e**, The EMC insertase and the Spf1 dislocase cavity bear complementary electrostatically charged surfaces. The residues delimiting the respective cavity are shown in surface representation and colored according to their electrostatic potential.

The structure of Spf1 within the complex was also consistent with previously reported Spf1 structures (Extended Data Fig. 4a,b; r.m.s.d. to PDB 6XMP: 1.0 Å)^32,36^. Spf1 is a P-type ATPase whose ability to translocate substrates is coupled to ATP hydrolysis and cycling between two major conformational states: E1 and E2 (refs. ^32,36^). In the absence of any nucleotide-state-stabilizing analog, Spf1 in complex with the EMC is in an E1 conformation, with its intramembrane cleft facing the cytosol. We observed the most prominent interaction interface between the ER lumenal parts of the EMC and Spf1, involving Emc7, Emc10 and the lumenal extensions of Spf1. This ‘lumenal dock’ buries around 424 Å^2^ of surface area (366 Å^2^ with Emc10, 58 Å^2^ with Emc7) (Fig. 2b,c). Formation of this interface requires the EMC and Spf1 to approach each other at an angle, inducing a 25° bend in the detergent micelle—a feature that was also visible in the reconstruction of the endogenous complex (Fig. 2b,c and Extended Data Fig. 3b).

A well-studied function of the EMC is the insertion of terminal transmembrane helices into the lipid bilayer^12,13,41^. This function has largely been attributed to an intramembrane hydrophilic vestibule enclosed by the subunits Emc3, Emc4 and Emc6, often referred to as the ‘gated cavity’ (refs. ^15,16,18,20^). Spf1, in contrast, has been shown to bind substrate helices within its intramembrane cleft before extraction^32,36^. In the EMC:Spf1 complex structure, these two experimentally validated sites for substrate engagement are positioned directly opposite of each other within the membrane, placing the insertase cavity of the EMC and the dislocase cleft of Spf1 in close spatial proximity (Fig. 2d and Extended Data Fig. 4c–e). This arrangement creates a large intramembrane space between the two proteins that resembles a funnel spanning the whole transmembrane region, with a width of 25 Å at the ER lumenal and 50 Å at the cytoplasmic sides (Extended Data Fig. 4d). The intermachinery space is laterally bordered by the Emc4 gate on one side, which approaches Spf1 at a 5-Å distance on the lumenal side. On the opposing side, Emc7 delimits the central vestibule, for which we resolved 12 more residues than previously reported^15,18^, yet could not detect the TMD and the subsequent C-terminal residues. Consistent with their distinct functions, the two substrate engagement sites display different electrostatic properties; while the Spf1 cleft is lined with negative charges, the hydrophilic vestibule of the EMC exhibits numerous positively charged residues (Extended Data Fig. 4e). Together, these observations indicate that EMC and Spf1 retain their individual substrate-binding sites while being arranged in a configuration that could facilitate coordinated insertion and extraction events and, because of its size, could accommodate complex clients with multiple TMDs.

### Molecular architecture of the EMC:Spf1 lumenal dock interface

**Fig. 3:**
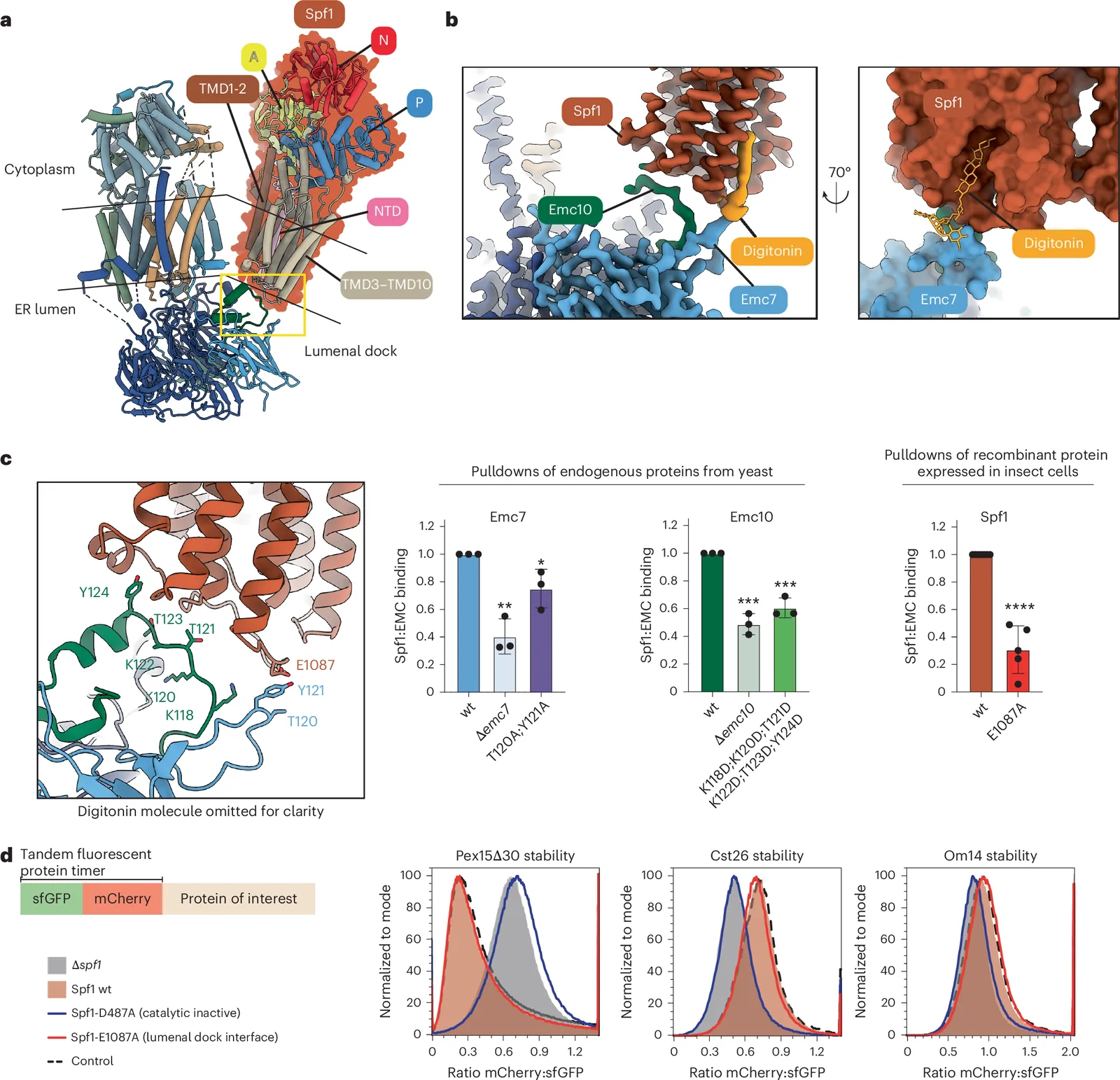
The interaction between the EMC and Spf1 is mediated by an ER lumenal interface. **a**, The EMC:Spf1 complex colored according to Spf1 domain architecture with the actuator (A) domain in yellow, the nucleotide-binding (N) domain in red, the phosphorylation (P) domain in blue, the N-terminal domain (NTD) in pink and the membrane domain in two shades of brown (Extended Data Fig. 4b). The substrate-binding site lies between TMD1–TMD2 (brown) and TMD3–TMD10 (sand). The lumenal dock region is marked with a yellow box. **b**, Left, cryo-EM density of the lumenal dock colored according to protein density. A digitonin molecule (orange) is bound to the back of Spf1, adjacent to its interface with Emc7. Right, detailed view of the bound detergent molecule lodged into a lateral pocket of Spf1. **c**, Mutational dissection of the lumenal dock. Deletion of *EMC7* or *EMC10* and mutations of the contact sites with Spf1 disrupt the complex in an endogenous setting in yeast. Similarly, introducing a single-point substitution in Spf1 strongly destabilizes the EMC:Spf1 complex when coexpressed in insect cells. The digitonin molecule was omitted in the structural representation for clarity. Binding was assessed in three independent replicates (five for Spf1-E1087A) and normalized to the respective wt. Data are presented as the mean ± s.d. Statistical analysis was conducted using an unpaired two-tailed Student’s *t*-test. *P < 0.05, **P < 0.01, ***P < 0.005 and ****P < 0.0001. Exact *P* values: *P* = 0.0013 for Emc7 wt versus *Δemc7*, *P* = 0.0366 for Emc7 wt versus Emc7_T120A;Y121A_, *P* = 0.0003 for Emc10 wt versus *Δemc10*, *P* = 0.0007 for Emc10 wt versus EMC10_K118D;K120D;T121D;K122D;T123D;Y124D_, *P* < 0.0001 for Spf1 wt versus Spf1-E1087A). **d**, Perturbation of the EMC:Spf1 interaction at the lumenal dock does not disrupt Spf1’s dislocase activity per se, as shown by the EMC-independent reporters Pex15Δ30, Cst26 and Om14. Protein stability was assessed by flow cytometry using a tandem fluorescent protein timer, where sfGFP matures quickly, reporting on translation, and mCherry matures more slowly; therefore, the ratio of mCherry to sfGFP gives a measure of protein stability. The constructs were encoded on a centromeric plasmid that was cotransformed with the respective Spf1 variant or an empty vector into an *Spf1*-KO strain. In the control condition, the G418 resistance was integrated into the HO locus and an empty vector was cotransformed. One representative histogram is shown here and a quantitative assessment, also of *Δemc3*, is shown in Extended Data Fig. 5f. Source data

Our data reveal the main site of interaction between the EMC and Spf1 to be confined to an interface at the ER lumenal side, where the lumenal regions of both proteins form a defined docking interface. This interface involves the ER lumenal parts of Spf1’s support domain (TMD7–TMD10), a loop in Emc7 (F114–L125) and a segment of the Emc10 linker region (K118–Y124), which connects the ER lumenal β-sandwich domain of Emc10 to its structurally unresolved TMD (Fig. 3a). Both subunits, Emc7 and Emc10, are considered accessory rather than core subunits of the EMC, meaning that loss of either leads to mild phenotypes and does not compromise the overall EMC complex stability^22,37^. In fact, Emc7 was first described as a client-specific chaperone rather than an EMC subunit^22^ and its deletion is associated with impaired membrane protein folding and export from the ER^42^. The function of Emc10 has remained elusive, yet its association with human diseases points to important physiological functions^43–48^. Additionally, we resolved a digitonin molecule at the lumenal dock, with its sterol backbone lodged into a lateral hydrophobic pocket on Spf1 and its glycosidic chains in close proximity to the Emc7 loop (Fig. 3b). A previous study resolved *n*-dodecyl-β-D-maltoside detergent molecules at the same position on Spf1 (ref. ^32^).

**Extended Data Fig. 5:**
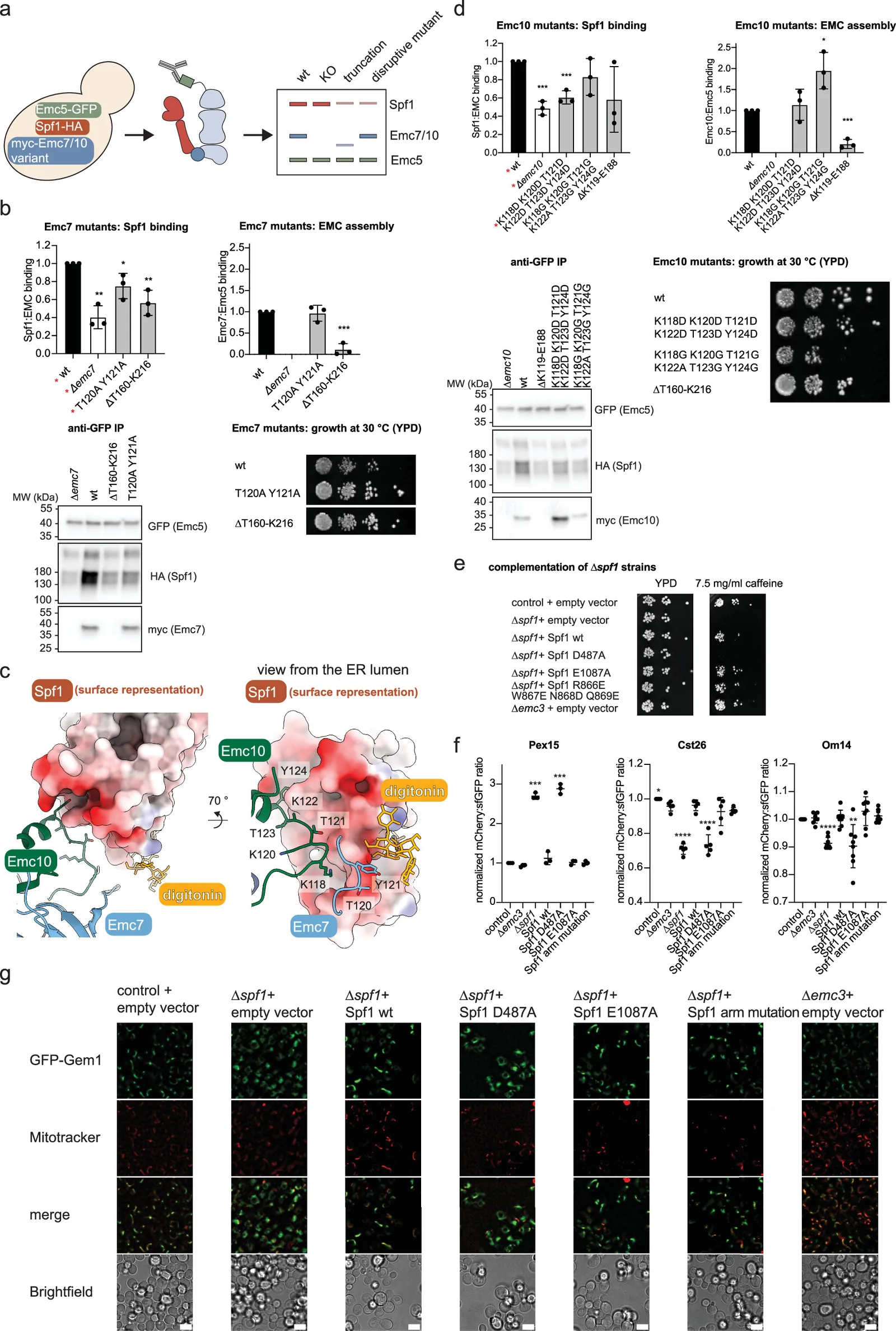
Validating the lumenal dock by mutagenesis of Emc7 and Emc10 in an endogenous setup in *S. cerevisiae*. **a**, Schematic depiction of the experimental setup to validate the lumenal interface in an endogenous yeast setting. Emc5 was tagged C-terminally with GFP, Spf1 with a triple HA-tag. *EMC7* or *EMC10* were either deleted or replaced at their endogenous locus with a version containing an N-terminal myc-tag, located after the signal sequence. The GFP was used as an affinity handle for co-IP experiments and binding of Spf1 and the Emc7/10 variant was assessed by western blot against the HA or myc tag, respectively. **b**, Overview of the effects of all mutations in Emc7 on the EMC-Spf1 interaction and overall EMC assembly. Binding was assessed in at least three independent replicates and normalized to the respective wt. Binding was assessed in three independent replicates and normalized to the respective wt. (mean ± standard deviation, unpaired two-tailed Student’s t-test * p < 0.05, ** p < 0.01, *** p < 0.005, **** p < 0.0001). A part of this data is also shown in Fig. 3c (indicated with red asterisks). Western blots of one representative replicate of anti-GFP IP are shown. A growth assay was performed to exclude effects of the Emc7 variants on yeast growth under standard conditions (YPD, 30 °C). **c**, View of the lumenal dock with Spf1 depicted as surface rendering, colored according to electrostatic potential. The view from the ER lumenal side shows the negatively charged lumenal surface of Spf1 that is contacted by Emc10 (green). Residues that were mutated in Emc10 are labeled. **d**, Overview of the effects of all mutations in Emc10 on the Emc-Spf1 interaction and overall EMC assembly as in panel **b**. **e**, The sensitivity to caffeine observed in *Δspf1* can be rescued by Spf1 variants expressed on a centromeric plasmid. Of note, the catalytically inactive D487A mutant does not rescue the phenotype, yet the wt and the two mutants that affect the Spf1:EMC interaction (Fig. 3c and Fig. 5d) do. **f**, Protein stability assay of different Spf1 clients fused to a tandem fluorescent protein timer (Fig. 3d). At least three independent biological replicates were performed and the mean mCherry:sfGFP ratio was normalized to the control condition (n = 3 for Pex15Δ30, n = 5 for Cst26, n = 8 for Om14). (mean ± standard deviation, unpaired two-tailed Student’s t-test comparing to Spf1 wt: * p < 0.05, ** p < 0.01, *** p < 0.005, **** p < 0.0001) In addition to the variants shown in Fig. 3d, we also show the effect of *Δemc3* and the Spf1 arm mutation (R866E W867E N868D Q869E) characterized in Fig. 5d. **g**, Microscopy of GFP-Gem1 in control vs. *Δspf1* strains reveals mislocalization in the absence of Spf1, whereas complementation with Spf1 wt and EMC-interface mutations rescues the phenotype. Scalebar 5 µm. The experiment was performed at least three times and one representative example is shown. Source data

To probe the key EMC:Spf1 interactions in cells, we generated yeast strains in which one of the EMC core subunits (Emc5) was C-terminally tagged with GFP, Spf1 was tagged with a C-terminal triple HA tag and variants of either Emc7 or Emc10 carried an N-terminal myc tag (Extended Data Fig. 5a). All genes were manipulated at their endogenous loci under their respective promoters to assess their effects in a setting as close to native conditions as possible. We assessed the integrity of the EMC and its interaction with Spf1 by comparing the ratios of Emc7 or Emc10 to Emc5 (as EMC integrity control) and Spf1 to Emc5 (as readout for mutational effects on insertase:dislocase association), respectively, in Emc5–GFP IPs. Deletion of Emc7 or Emc10 significantly weakened the Emc:Spf1 interaction to 40% and 48%, respectively (Fig. 3c and Extended Data Fig. 5b,d), which is in agreement with previous observations^22^. Substitution of two polar residues (T120A;Y121A) in the Spf1-contacting loop of Emc7 did not affect incorporation of this Emc7 variant into the EMC but significantly reduced Spf1 binding (Fig. 3c and Extended Data Fig. 5b), validating our structural insights. We additionally examined the traversing Emc10 linker region (K118–Y124), which contains multiple positively charged residues that could interact with the negatively charged ER lumenal surface of Spf1 (Extended Data Fig. 5c). By replacing the basic and polar residues in the respective Emc10 linker region with negatively charged aspartates, we expected to disrupt the Emc10:Spf1 interface because of charge repulsion, while not affecting the overall EMC assembly, which was confirmed by our co-IP data (Fig. 3c and Extended Data Fig. 5d).

Endogenous Spf1 was genetically less accessible than the EMC for introducing mutations in yeast; hence, we used the recombinant insect cell system to test the effects of complementary mutations in Spf1. The E1087 residue in Spf1 faces the polar Emc7 loop, suggesting that it may contribute to the EMC:Spf1 interaction. Indeed, substitution of this glutamate by alanine significantly reduced the EMC:Spf1 interaction to 30% (Fig. 3c). This strong effect may also partially reflect disruption of the detergent-binding site, where the glycosidic chains of the resolved digitonin are in close proximity (Fig. 3b).

As EMC and Spf1 form a stable complex, we assessed whether perturbing the EMC:Spf1 interface affects Spf1 function in cells. For this, we examined several established phenotypes such as caffeine sensitivity^49^ and mislocalization of the mitochondrial TA protein Gem1 (ref. ^38^). The Spf1 lumenal dock mutant (E1087A) showed a similar behavior to the strain with wild-type (wt) Spf1, in contrast to controls that eliminate Spf1 catalytic activity (D487A) or the unrescued *Spf1*-KO strain (Extended Data Fig. 5e,g). After testing for equal expression of all Spf1 variants (Supplementary Fig. 12b), we further confirmed Spf1’s functional integrity using an established client protein stability assay based on the well-characterized TA model protein Pex15Δ30. This protein usually localizes to mitochondria^27^ and was found to be stabilized and ER-mislocalized in the absence of Spf1 (ref. ^50^). In agreement with published data, our assays show that Pex15Δ30 was stabilized in the unrescued *Spf1*-KO strain or when disrupting the catalytic activity (D487A), whereas complementation with either the wt or the lumenal dock mutant could restore this phenotype to control levels (Fig. 3d and Extended Data Fig. 5f). Pex15Δ30 insertion into the ER depends on the GET pathway rather than the EMC^51^; hence, disruption of the EMC:Spf1 interface is not expected to influence this substrate, consistent with our observations (Extended Data Fig. 5f). We extended this protein stability assay to more clients: Cst26, a lipid droplet localized protein that was reported to be depleted in the absence of Spf1 (ref. ^52^), and Om14, a mitochondrial multipass TM protein that we found to interact with both the EMC and Spf1 in our proteomic analyses (Supplementary Table 1). Both proteins showed significant destabilization in *Δspf1*, which could be rescued by complementing with Spf1 wt or the lumenal dock mutant (Fig. 3d and Extended Data Fig. 5f), supporting the notion that this mutant does not affect Spf1’s structure and catalytic function per se.

Taken together, our mutational analysis indicates that disruption of the EMC:Spf1 interface weakens the association between the two machineries but leaves core functions of Spf1 intact. These findings indicate that EMC and Spf1 can assemble into a higher-order complex without compromising their individual activities, consistent with a model in which membrane protein biogenesis and quality control machineries are spatially coordinated within the ER membrane.

### EMC:Spf1 assembly is conserved in human cells

**Fig. 4:**
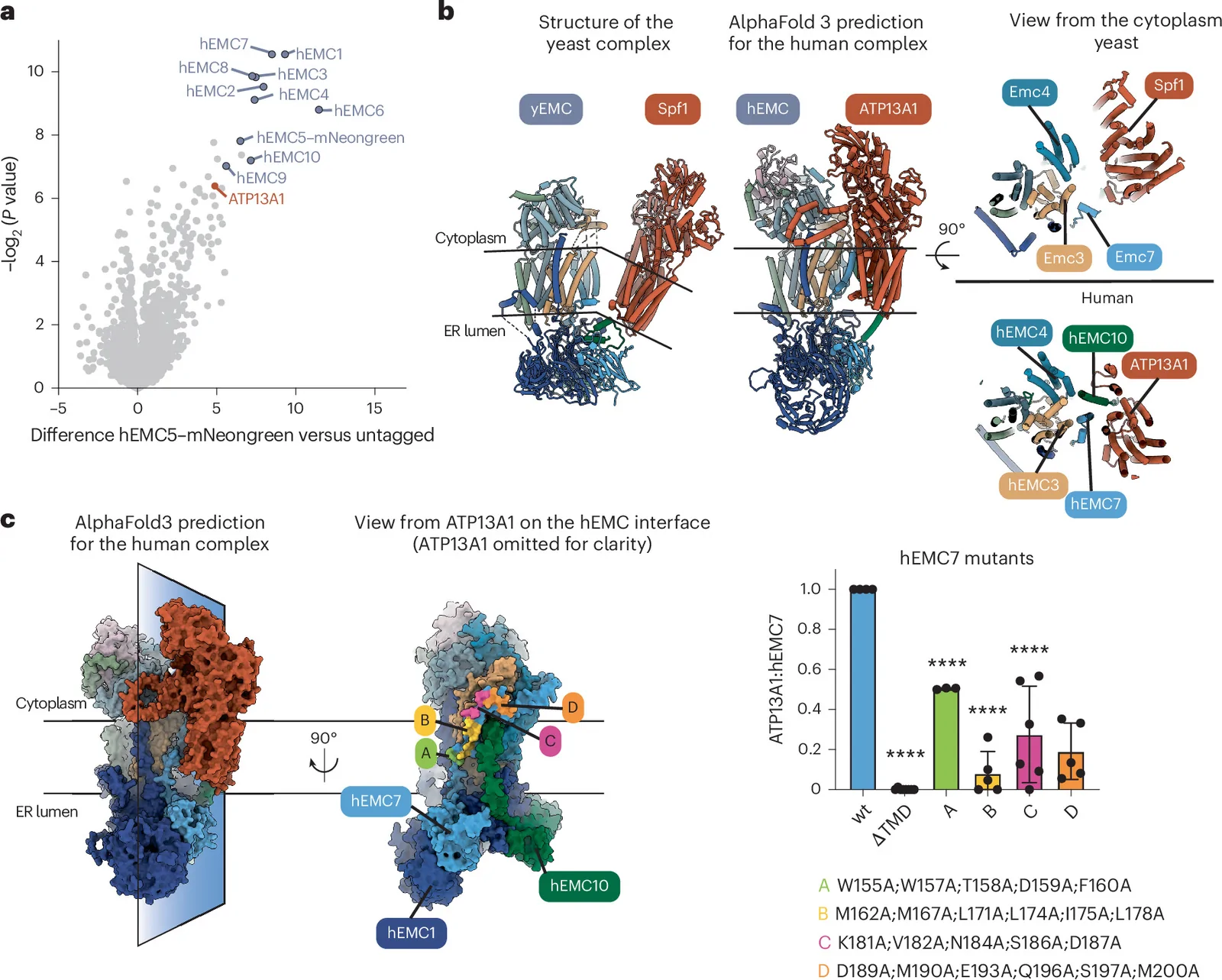
The EMC:Spf1 complex is conserved in human cells. **a**, The interactome of endogenously tagged hEMC5–mNeongreen in HEK293T cells reveals ATP13A1, the human Spf1 ortholog, as a major interaction partner. A two-tailed unpaired Student’s *t*-test was applied to compare the interactome of hEMC5 to values measured for an untagged cell line. **b**, The experimentally obtained structure of the yeast EMC:Spf1 complex (left) in comparison to a model of the hEMC:ATP13A1 complex generated by AlphaFold 3 (middle) shows architectural conservation of the complex across species. Right, a view of the complex from the cytoplasm depicts the arrangement of the TMDs in the yeast (top) and human (bottom) complex inside the lipid bilayer and shows the proximity of the TMDs of hEMC7 and hEMC10 to ATP13A1. **c**, Structural model of the hEMC:ATP13A1 complex as in **b** but shown in a space-filling representation. Left, a section through the model was taken and the side of the hEMC facing ATP13A1 is shown. Right, substitutions introduced into hEMC7 are highlighted in the structural model (A–D) and their effect on endogenous ATP13A1 binding was assessed by co-IP in HEK293T cells. Binding experiments were performed in at least three independent replicates (*n* = 8 for ΔTMD, *n* = 3 for A and *n* = 5 for B–D) and normalized to the respective wt. Data are presented as the mean ± s.d. Statistical analysis was conducted using an unpaired two-tailed Student’s *t*-test. ****P < 0.0001. Structural details and effects on hEMC assembly are shown in Extended Data Fig. 6d. Source data

Our data revealed that the EMC and Spf1 form a stable complex in yeast. To test for evolutionary conservation in human cells, we endogenously tagged hEMC5 with mNeongreen in HEK293T cells and performed an IP–MS experiment (Fig. 4a). Consistent with our findings in yeast, ATP13A1 (the human ortholog of yeast Spf1) was a main interactor of the hEMC (Fig. 4a and Supplementary Table 2).

To obtain a structural model of the hEMC:ATP13A1 supercomplex, we used AlphaFold 3 (ref. ^53^) (Supplementary Fig. 6). Of note, an AlphaFold 3 model of the yeast complex was consistent with our experimental structure, showing that the overall architecture of this complex can be predicted by AlphaFold 3 (Supplementary Fig. 7). The prediction placed the hEMC and ATP13A1 in a similar orientation toward each other as observed for the yeast complex, with the insertase and the dislocase cavities facing each other (Fig. 4b and Supplementary Fig. 6). The major interaction interface observed in the yeast complex, the lumenal dock, was partially recapitulated in the human model (Fig. 4b), with hEMC10 contacting ATP13A1 from the lumenal side in a similar fashion as in the yeast structure.

**Extended Data Fig. 6:**
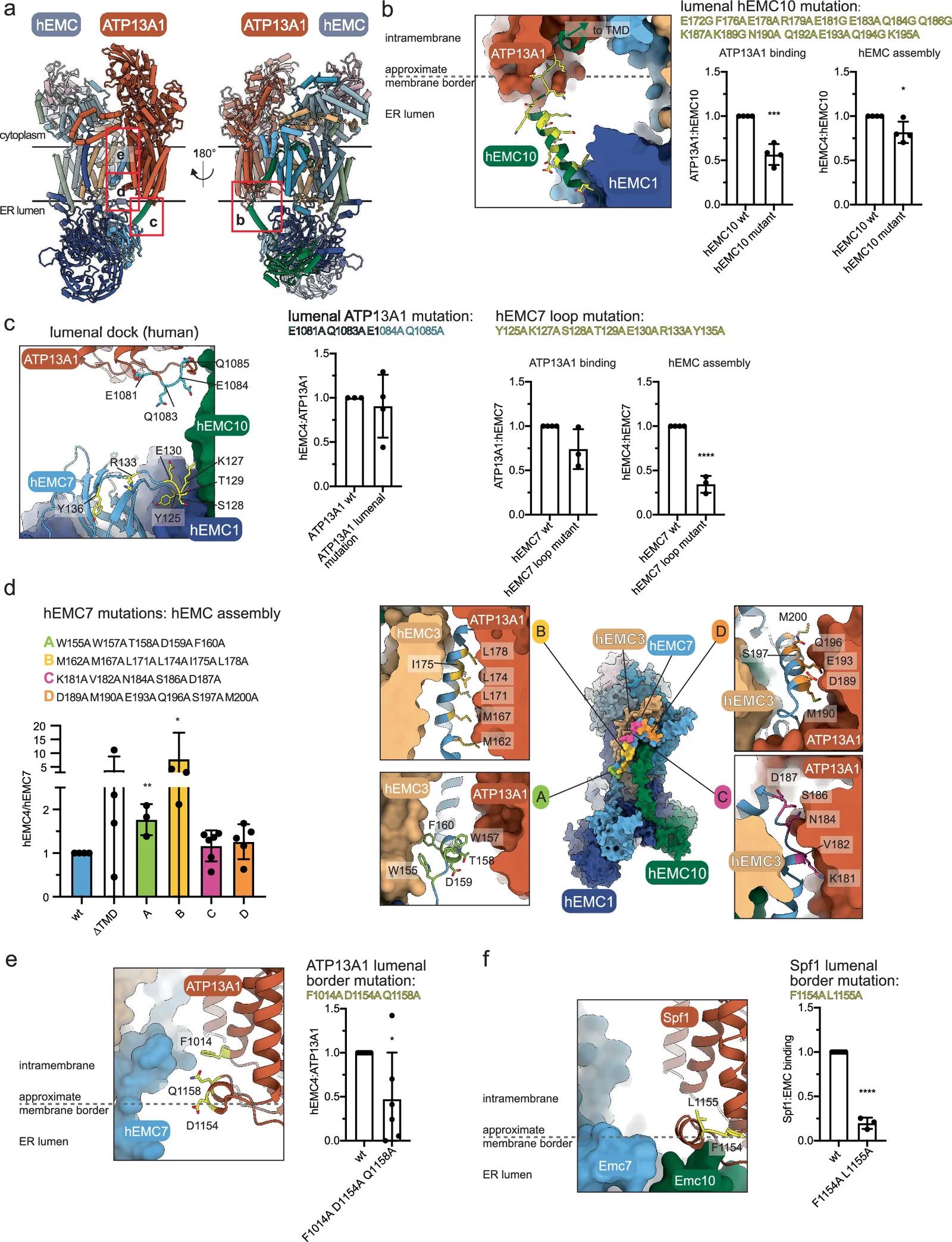
Mutational dissection of the AlphaFold 3 model of the hEMC:ATP13A1 complex. **a**, Predicted model of the hEMC:ATP13A1 complex from two views as shown in Fig. 4b. To orient the reader, regions that were mutated and are shown in detail in the following panels are marked with red frames and the letters labeling that panel are included. Approximate membrane borders are marked with black lines. **b**, Mutation of the lumenal linker and helix region in hEMC10 affects ATP13A1 binding in cells and mildly compromises hEMC assembly. Mutated residues are highlighted in yellow. Binding was assessed via co-IP on hEMC10 in four independent replicates and normalized to the wt. (mean ± standard deviation, unpaired two-tailed Student’s t-test * p < 0.05, ** p < 0.01, *** p < 0.005, **** p < 0.0001; exact p-values for ATP13A1 binding p = 0.0003 for hEMC assembly p = 0.0231). **c**, View of the lumenal dock in the human complex. Aquamarine: Acidic residues in ATP13A1 that were mutated and had no effect on hEMC binding (bar graph on the right). Yellow: Residues in the region of hEMC7, corresponding to the Emc7 loop in yeast, whose mutation resulted in reduced association with the hEMC yet had no significant effect on hEMC binding (bar graphs on the bottom). Binding was assessed via co-IP on ATP13A1 or hEMC7, respectively in three (hEMC7 mutation) or four (ATP13A1 mutation) independent replicates and normalized to the respective wt. (mean ± standard deviation, unpaired two-tailed Student’s t-test * p < 0.05, ** p < 0.01, *** p < 0.005, **** p < 0.0001; exact p-values for lumenal ATP13A1 mutant p = 0.6703, for ATP13A1 binding of hEMC7 loop mutant p = 0.0632, for hEMC assembly of hEMC7 loop mutant p < 0.0001). **d**, Left: Effect of mutations in hEMC7 shown in Fig. 4c on hEMC assembly. Deletion of the TMD or mutation of subregions thereof had a stabilizing or no effect. Binding was assessed via co-IP on hEMC7 in at least three independent replicates (n = 3 for A, B; n = 4 for ΔTMD, n = 5 for D, n = 6 for C) and normalized to the wt. (mean ± standard deviation, unpaired two-tailed Student’s t-test * p < 0.05, ** p < 0.01, *** p < 0.005, **** p < 0.0001; exact p-values for hEMC assembly p = 0.0678 for ΔTMD, p = 0.0068 for A, p = 0.0316 for B, p = 0.1504 for C, p = 0.0525 for D). Right: detailed depiction of the residues mutated in the individual segments of hEMC7. **e**, Mutation of selected residues in ATP13A1 (highlighted in yellow) at the border between the transmembrane region and ER lumen destabilizes complex formation with the hEMC. Binding was assessed via co-IP on ATP13A1 in six independent replicates and normalized to the wt. (mean ± standard deviation, unpaired two-tailed Student’s t-test * p < 0.05; exact p-value p = 0.0226). **f**, Mutation of residues in Spf1 at the border between the transmembrane region and ER lumen, corresponding to the mutation shown in e in ATP13A1. Binding was assessed via co-IP on Spf1 expressed recombinantly in insect cells in three independent replicates and normalized to the wt. (mean ± standard deviation, unpaired two-tailed Student’s t-test **** p < 0.0001). Source data

To validate the predicted lumenal interaction experimentally, we replaced the charged residues within this lumenal segment of hEMC10 (E172–K195) with neutral amino acids (Supplementary Figs. 8b and 9b,c). Consistent with the structural model, these substitutions weakened the interaction between hEMC and ATP13A1, while only mildly affecting overall EMC assembly (Extended Data Fig. 6a,b).

We next investigated the lumenal loop region of Emc7, which was critical for the yeast EMC:Spf1 interaction (Fig. 3c). This loop is shorter in hEMC7 (Supplementary Figs. 8a and 9a) and was not predicted to contact ATP13A1 in the AlphaFold model. Indeed, comprehensive mutagenesis of the loop in hEMC7 and the complementary interface in ATP13A1, corresponding to the highly effective Spf1-E1087A mutant in yeast, did not affect complex formation (Extended Data Fig. 6c).

**Extended Data Fig. 7:**
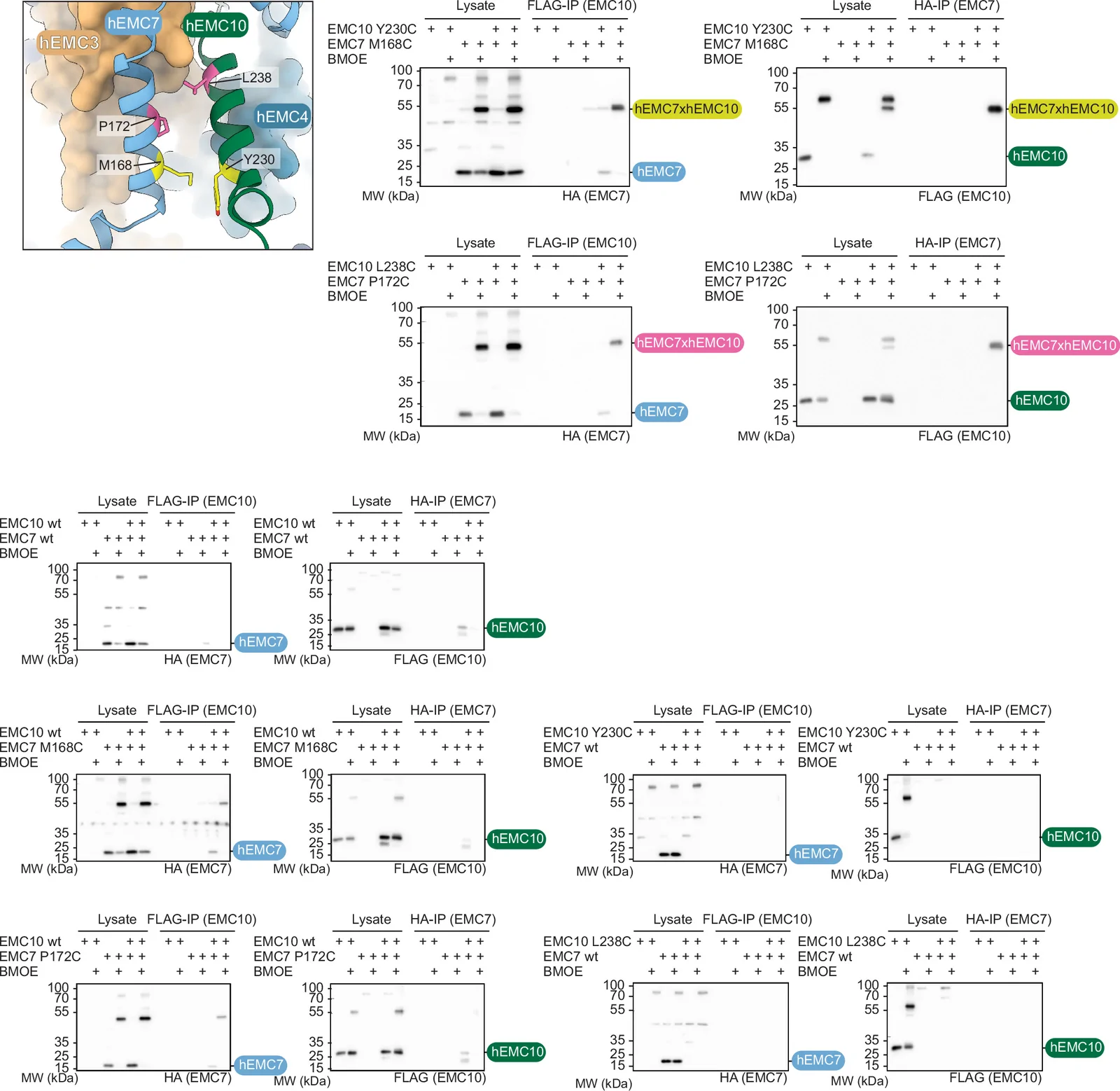
Intramembrane cysteine crosslinking between hEMC7 and hEMC10 confirms the predicted TMD interface. Single residues in hEMC7 (M168, P172) and hEMC10 (Y230, L238) were replaced with cysteines and in-cell crosslinking was performed using BMOE. Crosslinked adducts were isolated by IP and analyzed on a western blot, confirming the presence of a successful cysteine crosslink between the two tagged subunits. hEMC10 contains 4 cysteines in its wt sequence. To exclude the possibility of bands arising from crosslinking with other cysteines than the ones introduced or unspecific interaction, a combination of hEMC7 and hEMC10 without any further Cys mutation was assessed and also each Cys-variant was tested with the wt of the other protein. We note an additional crosslinked product of EMC7 M168C and P172C with an uncharacterized partner that results in a similar molecular weight to the EMC7xEMC10 adduct yet is not positive for EMC10-FLAG. The experiment was performed in independent replicates, at least three times per cysteine position, and one representative replicate is shown.

These results suggested that, while the overall EMC:ATP13A1 architecture is conserved, partially different interfaces stabilize it; the lumenal dock seems to be less important in the human context, yet the structural prediction suggests an extended intramembrane interface between the hEMC and ATP13A1 (Fig. 4b). This different interface format may also explain the lack of a predicted tilt between hEMC and ATP13A1, as opposed to the yeast structure. The predicted intramembrane interface involves regions of the hEMC that have not been resolved in experimental structures, namely the TMDs of hEMC7 and hEMC10. Thus, to first validate the predicted positioning of these TMDs experimentally, we introduced cysteine pairs into the TMDs of hEMC7 and hEMC10 and performed in-cell crosslinking with the bismaleimide-based crosslinker BMOE (Extended Data Fig. 7). The observed crosslinks were fully consistent with the predicted direct interaction between the TMDs of hEMC7 and hEMC10 and with previously reported experimental evidence for the positioning of the TMD of hEMC7 (refs. ^17,21^). Building on this validation of the hEMC model, we next analyzed the predicted hEMC:ATP13A1 intramembrane interface in more detail. This interface mainly involves the transmembrane regions of hEMC7 (Fig. 4c and Extended Data Fig. 6d). Deletion of the entire TMD and C-terminal region of hEMC7 (Δ159–242) resulted in a complete loss of interaction between the hEMC and ATP13A1 and, unexpectedly, in a slightly stronger association of this hEMC7 variant with the overall hEMC (Fig. 4c and Extended Data Fig. 6d). A deletion of the equivalent Emc7 TMD in yeast also weakened Spf1 binding but, in contrast to the human complex, negatively impacted the overall EMC assembly (Extended Data Fig. 5b). To dissect this hEMC7:ATP13A1 intramembrane interface in more detail, we introduced substitutions into the individual segments of the hEMC7 TMD and C terminus and assessed their effects on interaction with ATP13A1 and on hEMC assembly (Fig. 4c and Extended Data Fig. 6d). This analysis further supported the predicted structural model and defined multiple contact regions between hEMC7 and ATP13A1 within the transmembrane and cytoplasmic regions that contributed to complex stability (Fig. 4c and Extended Data Fig. 6d). In ATP13A1, a reciprocal substitution at the border between the membrane region and the ER lumen halved the interaction with the hEMC (Extended Data Fig. 6e). This substitution was also transferred to the yeast Spf1 and assessed in the recombinant insect cell setup, showing reduced complex formation (Extended Data Fig. 6f). Together, these findings indicate that the EMC:Spf1 complex identified in yeast is conserved in human cells as an hEMC:ATP13A1 complex. The overall organization of the two machineries appears preserved and involves the same subunits of the EMC in mediating the interaction, yet the precise interaction interfaces show species-specific adaptations.

### ATPase-driven conformational changes in Spf1 reshape the EMC:Spf1 supercomplex

**Fig. 5:**
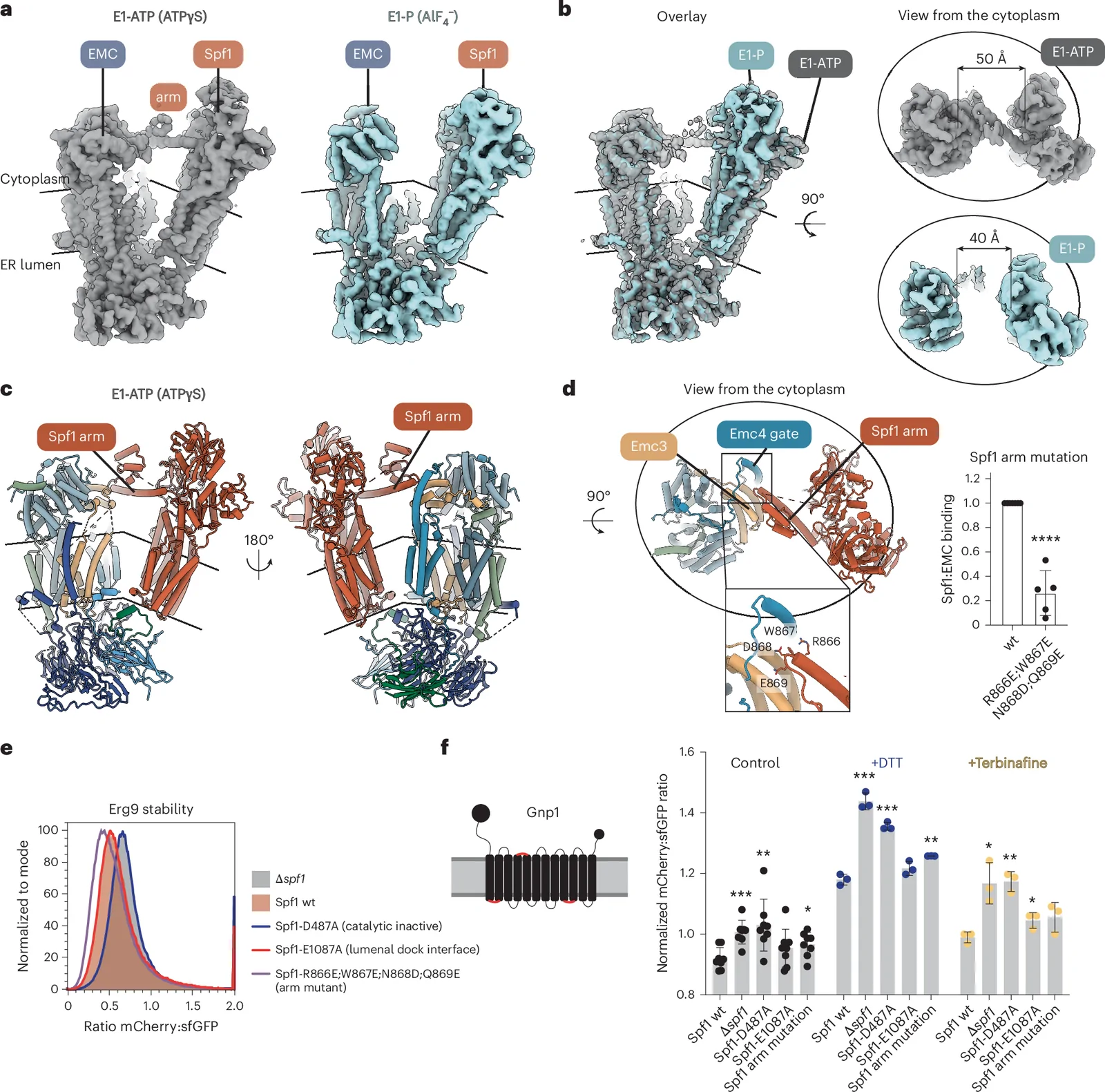
Nucleotide binding and hydrolysis by the P-type ATPase Spf1 remodels the EMC:Spf1 complex and controls cytoplasmic access to the adjacent insertase and dislocase intramembrane cavities. **a**, Cryo-EM maps of the EMC:Spf1 complex bound to the nucleotide analogs ATPγS (left; gray) and AlF_4_^−^ (right; aquamarine), mimicking the E1-ATP and the E1-P state, respectively. In the E1-ATP state, the arm domain of Spf1 is visible, extending from Spf1 through the cytoplasm to the cytoplasmic cap of the EMC. Approximate boundaries of the detergent micelle are indicated with black lines. **b**, Overlay and direct comparison of the two different nucleotide states show how the arm domain of Spf1 can function as a lid in the E1-ATP state, blocking entry from the cytoplasm toward the composite intramembrane cavity of the complex. In the E1-P state, the arm domain was not observed, presumably due to conformational heterogeneity, and the size of the entrance to the cavity is reduced by 10 Å because of repositioning of Spf1 toward the EMC within the micelle. Approximate boundaries of the detergent micelle are indicated with black lines. **c**, The EMC:Spf1^ATPγS^ structure from two different views shows the arm domain of Spf1, which contacts the EMC at the cytosolic cap between Emc3 and Emc4 and blocks access to the juxtaposed substrate-binding cavities. **d**, Top view from the cytoplasm of the arm of Spf1 (cartoon representation), contacting the EMC. The close-up view shows the tip of the arm, with substituted residues labeled. Binding of the Spf1 arm mutant to the EMC was assessed in the recombinant insect cell system in five independent replicates and normalized to the Spf1 wt. Data are presented as the mean ± s.d. Statistical analysis was conducted using an unpaired two-tailed Student’s *t*-test. ****P < 0.0001. **e**, The ER TA protein Erg9 is stabilized in the context of inactive Spf1 or an unrescued *Spf1* KO, as assessed by a fluorescent protein stability reporter experiment in flow cytometry. Disrupting the EMC:Spf1 interface at the lumenal dock does not affect Erg9 levels, yet mutating the arm seems to destabilize the client. **f**, Stability of the multipass TM protein Gnp1 under normal (control) conditions and cellular stress induced by treatment with DTT (induction of the unfolded protein response) or terbinafine (ergosterol biosynthesis inhibitor). The schematic shows the topology of Gnp1 and particularly short loops (2–4 aa) between two TMDs are highlighted in red. While Gnp1 is stabilized in the absence of Spf1, the two mutants at the EMC:Spf1 interface show incomplete rescue to wt levels under stress conditions. Exemplary histograms and the effect of *Δemc3* are shown in Extended Data Fig. 8d,e. Protein stability was assessed in at least three independent replicates (*n* = 8 for control, *n* = 3 for DTT and terbinafine) and normalized to the control condition. Data are presented as the mean ± s.d. Statistical analysis was conducted using an unpaired two-tailed Student’s *t*-test compared to Spf1 wt. *P < 0.05, **P < 0.01 and ***P < 0.001. Source data

Spf1 is a P-type ATPase that follows the Post–Albers cycle for substrate translocation, cycling between two major states: E1 and E2. In the E1 state, the substrate-binding cleft is accessible from the cytoplasm, whereas, in the E2 state, it faces the ER lumenal side^32,36^. To examine whether Spf1 can adopt its different nucleotide states while associated with EMC, we determined cryo-EM structures of the complex in the presence of the ATP analog ATPγS to a global resolution of 3.8 Å (Fig. 5a, Supplementary Fig. 10 and Table 1) and obtained a low-resolution structure in the presence of the ATP hydrolysis transition state analog AlF_4_^−^ (global resolution: 6.8 Å) (Fig. 5a, Supplementary Fig. 11 and Table 1). Biochemical trapping of the complex in the E2 state or intermediates of such using other nucleotide analogs did not yield a sample that allowed structure determination by cryo-EM.

**Extended Data Fig. 8:**
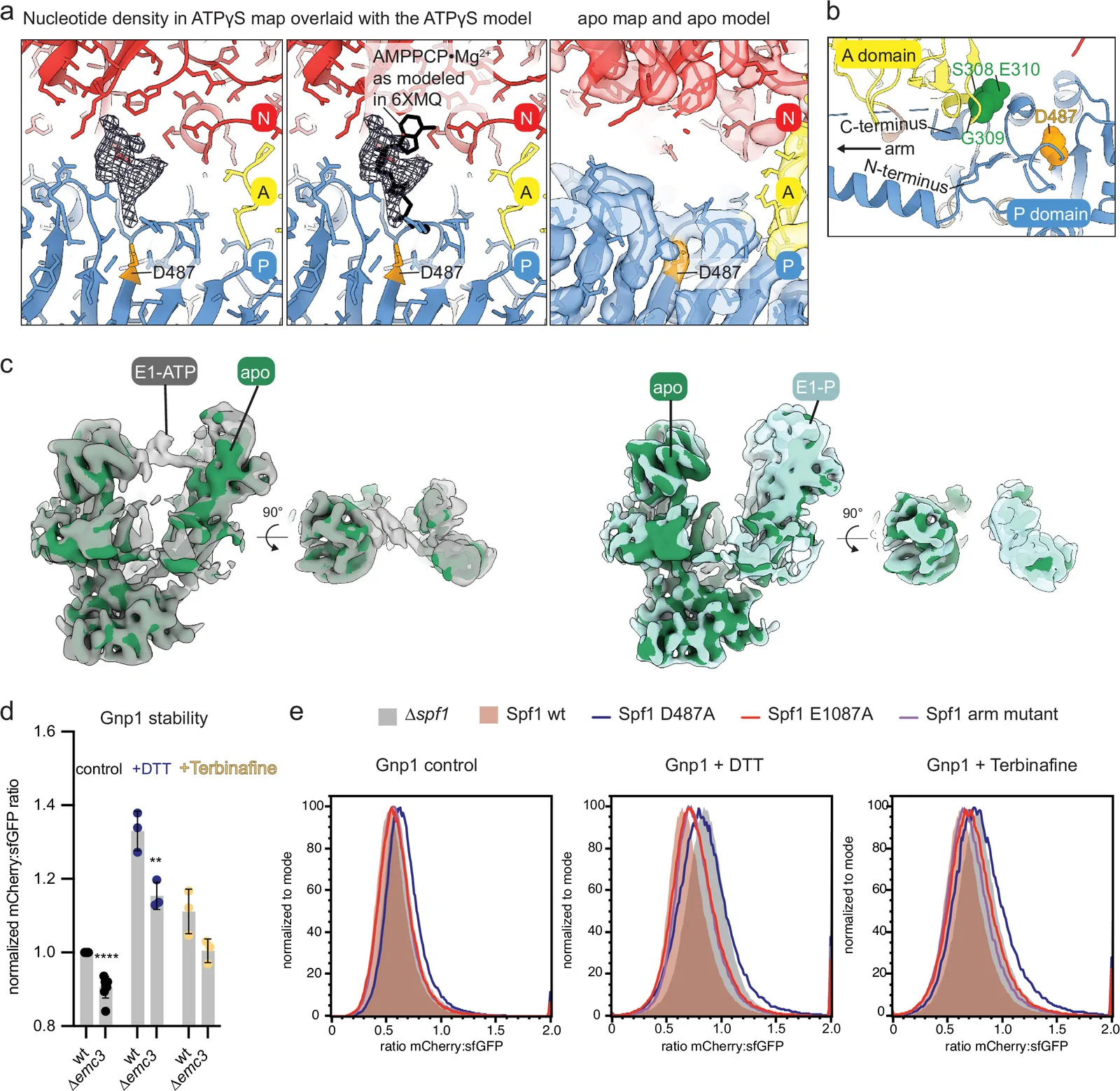
Nucleotide binding to Spf1 and its effect on EMC:Spf1 supercomplex conformation. **a**, The cryo-EM map of the EMC:Spf1 complex in the presence of ATPγS reveals density in the nucleotide binding pocket. A map was generated based on the model built for the E1-ATP complex and subtracted from the experimentally obtained map to clearly display the additional density (grey mesh). The middle panel shows the AMPPCP nucleotide as modeled in the PDB: 6XMQ, overlapping with the additional density in our map. Right: the additional putative nucleotide density is completely absent in the apo map. The catalytic aspartate is highlighted in orange, backbone colors were chosen according to domain identity as in Fig. 3 (N in red, A in yellow, P in blue). **b**, The Spf1 arm domain is positioned in closeproximity to core catalytic components of the P-type ATPase. The catalytic aspartate is highlighted in orange, the SGE dephosphorylation motif in green. Backbone colors were chosen according to domain identity as in Fig. 3 (A in yellow, P in blue). **c**, Overlay of the cryo-EM maps of the EMC:Spf1 complex bound to the nucleotide analogs ATPγS (grey) or AlF_4_^−^ (aquamarine) and the last consensus map of the apo state (green). All maps were low pass filtered to a resolution of 10 Å for more accurate comparison of domain positioning at the same resolution. Repositioning of Spf1 and the size difference in the entry to composite cavity is most pronounced in the comparison of the ATPγS- and AlF_4_^−^-bound states (as shown in Fig. 5a, b). At this resolution, the positioning of Spf1 in the apo state is largely similar to the ATPγS-bound state, with the notable difference of the arm domain being resolved in the latter. **d**, The Gnp1 reporter is depleted in *Δemc3* under control conditions and DTT stress. Protein stability was assessed in at least 3 independent replicates (n = 8 for control, n = 3 for DTT and terbinafine conditions, respectively) and normalized to the control condition. (unpaired two-tailed Student’s t-test ** p < 0.01, **** p < 0.0001; exact p-values for control p < 0.0001, for DTT p = 0.0095, for Terbinafine p = 0.0539). **e**, Representative histograms of the Gnp1 reporter in *Δspf1* cells rescued with different Spf1 variants. The quantitative assessment is shown in Fig. 5f. Source data

The EMC:Spf1^ATPγS^ complex adopts an E1-ATP-like conformation that closely resembles a previously reported structure of Spf1 in this state (r.m.s.d. to PDB 6XMQ: 0.926 Å). Consistent with nucleotide binding, we detected density within the nucleotide-binding pocket (Extended Data Fig. 8a), albeit at a resolution that was not sufficient for precise model building of the nucleotide analog. The presence of AlF_4_^−^ likely mimicked the E1-P state, as reported previously^32^, although this cannot be confirmed at this resolution. While the overall architecture is conserved across different states, the different nucleotide states also show distinct structural characteristics. In the EMC:Spf1^ATPγS^ state, we observed well-defined density for nearly the entire sequence of the Spf1 arm (Supplementary Figs. 5b and 8c)—an insertion in the P-domain (L834–G956) exclusive to P5A-ATPases. The SGE dephosphorylation motif of the A-domain was nestled between the N-terminal and C-terminal segments of this arm domain and the catalytic D487 is within ~15 Å of the arm (Extended Data Fig. 8b). In contrast, density for this arm, as well as the cytoplasmic helices of Emc3 that are contacting the arm in the EMC:Spf1^ATPγS^ complex, was completely absent in the EMC:Spf1^AlF4−^ complex. Comparison of the nucleotide states revealed substantial repositioning of Spf1 relative to the EMC, with the transmembrane core and cytoplasmic domains of Spf1 shifting by approximately 10 Å toward the EMC after ATP hydrolysis (Fig. 5b). Of note, the relative conformation of Spf1 toward the EMC in the apo state closely resembled the structure of the ATPγS-bound state (Extended Data Fig. 8c).

**Extended Data Fig. 9:**
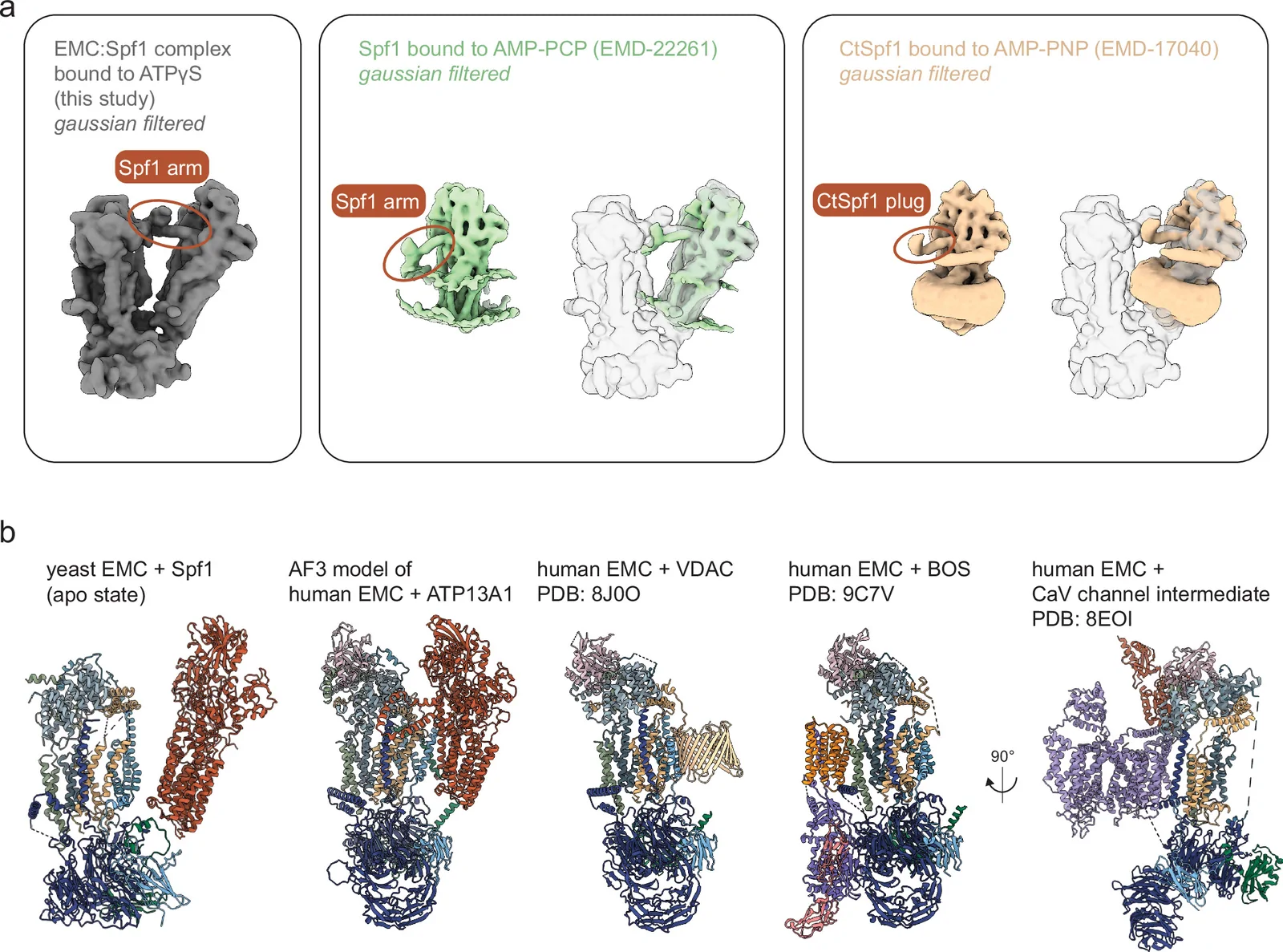
Comparison of the EMC:Spf1 complex to previously reported structures of Spf1 and of different EMC supercomplexes. **a**. The Spf1 arm domain is stabilized in the E1-ATP state in the EMC:Spf1 complex, allowing structural characterization and modeling. A comparison to lower resolution reports of the Spf1 arm^33^ or equivalent CtSpf1 “plug” domain^37^ shows consistent positioning in the respective E1-ATP state. A Gaussian filter was applied to all maps to allow comparison at a similar resolution level. **b**, Comparison of the EMC:Spf1 supercomplex from yeast and the predicted hEMC:ATP13A1 supercomplex to other EMC supercomplexes that have been structurally characterized^10,23,57^.

The Spf1 arm forms an extended helical protrusion with a globular domain at its tip. In our EMC:Spf1^ATPγS^ structure, the arm spans the cytoplasmic space between Spf1 and the EMC, contacting Emc3 at the cytoplasmic cap and the Emc4 gate (Fig. 5c,d and Supplementary Fig. 5b). Previous structures of Spf1 in the E1-ATP state resolved the arm (or plug) domain at a low resolution^32,36^ (Extended Data Fig. 9a). In those structures, the arm domain points toward the detergent micelle, whereas, in our map, the arm is well resolved and forms a nucleotide-dependent contact with the EMC. Notably, we observed substantially more density for Emc3 and Emc4, contacting the arm, in the presence of ATPγS in comparison to the apo map.

To corroborate this nucleotide-dependent interaction interface, we introduced substitutions at the tip of the Spf1 arm to disrupt interactions with Emc3 and Emc4. Emc3 contains a negatively charged patch at the interaction site and introduction of negative charges into the tip of the Spf1 arm domain led to significant destabilization of the complex, potentially because of electrostatic repulsion (Fig. 5d). Despite the reduced association, the arm mutant performed comparably to wt Spf1 in the assays described above that assess core Spf1 functions that are EMC independent (Fig. 3d and Extended Data Fig. 5e–g).

We next examined the impact of mutations of the arm and the lumenal dock on EMC client proteins. Erg9, the yeast ortholog of human squalene synthase (SQS/FDFT1), is a well-characterized EMC TA client in humans and also interacts with yeast EMC^22^. Interestingly, Erg9 was destabilized when the EMC:arm interface was mutated and stabilized in the context of inactive Spf1 (D487A) or an unrescued *Spf1*-KO strain (Fig. 5e). We also investigated the multipass EMC client Gnp1 (refs. ^15,54^) and confirmed destabilization of the reporter construct in *Δemc3* cells and stabilization in *Δspf1 cells* (Extended Data Fig. 8d and Fig. 5f). In contrast to the effects observed for Erg9, there was a slight (5%) increase in Gnp1 stability in the strain with the arm mutation compared to Spf1 wt, indicating that this mutant does not fully recapitulate the wt Spf1 rescue effects of the *Spf1*-KO phenotype (Fig. 5f and Extended Data Fig. 8e). This effect became more pronounced when subjecting the cells to ER stress induced by DTT. Similarly, the lumenal dock substitution (E1087A) led to stabilization of Gnp1 when ergosterol biosynthesis was inhibited by terbinafine. These findings indicate that the EMC:Spf1 interaction may be particularly important under conditions that challenge membrane protein homeostasis.

Together, our data show that the EMC:Spf1 assembly can accommodate multiple nucleotide-dependent conformations of Spf1 during its ATPase cycle. The Spf1 arm establishes a nucleotide-sensitive contact with EMC, without being required for Spf1’s catalytic activity. These observations suggest that dynamic interactions between the insertase EMC and dislocase Spf1 may contribute to the coordination of insertion and extraction at the ER membrane, which may be needed because both molecular machines in the supercomplex have opposing activities.

## Discussion

**Fig. 6:**
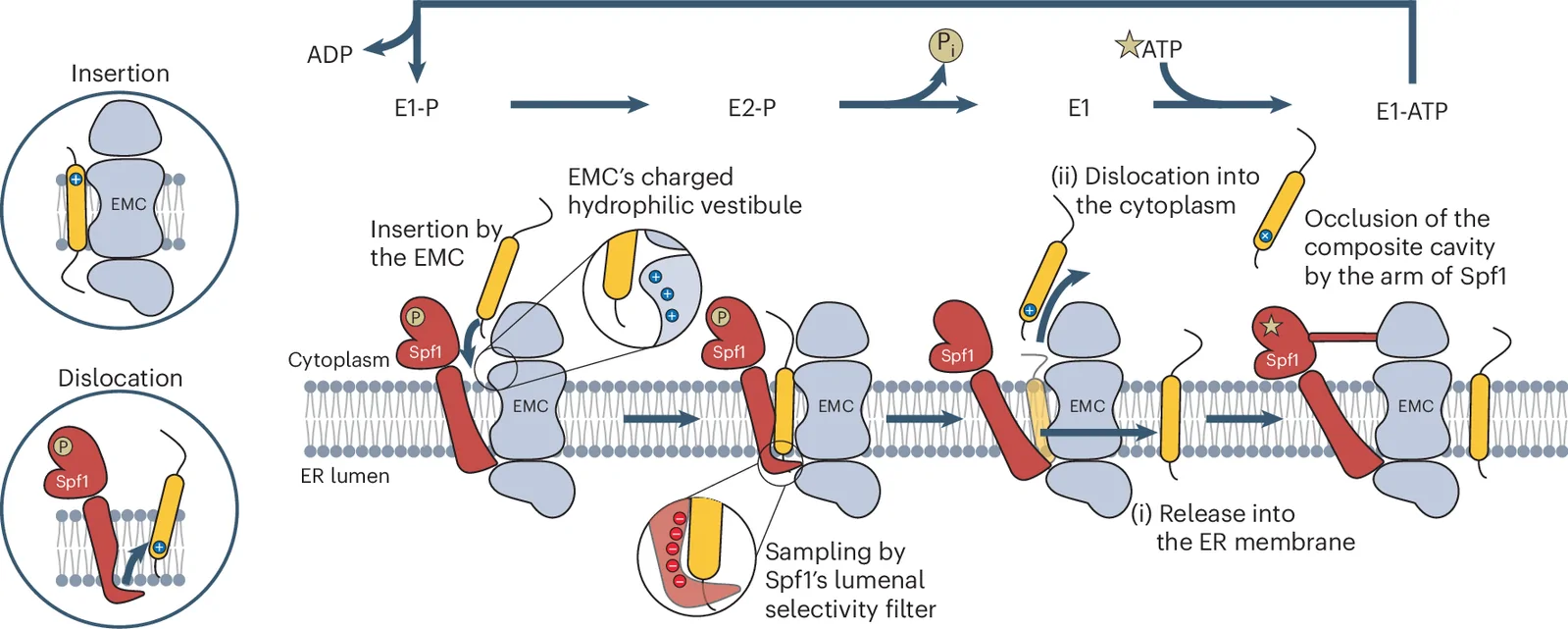
Possible conceptual model for an ATP-driven substrate translocation cycle by the EMC:Spf1 complex. The composite transmembrane cavity of the EMC:Spf1 complex in its E1-P conformation is well accessible from the cytoplasmic side and allows the EMC to engage substrates, probe them with its selectivity filter inside the hydrophilic vestibule^21^ and insert them into the lipid bilayer. In the E2-P state, Spf1’s substrate-binding site is facing the ER lumen. This intramembrane cleft is rich in negative charges that interact with the lumen-proximal end of the substrate TMD. These features, in combination with our structural analyses, allow us to propose a hypothetical model for functions of the EMC:Spf1 supercomplex: If no binding to Spf1 occurs, the substrate may be released into the ER membrane (**i**). If the lumen-proximal part of the substrate is positively charged, it may bind to Spf1 and induce a conformational shift back into the E1 state with the substrate-binding site facing the cytosol and expelling the substrate and a phosphate (**ii**). Subsequent ATP binding stabilizes the Spf1 arm domain in a conformation that restricts the apparent cytoplasmic access path to the intermachinery space and, hence, prevents immediate reinsertion. Alternatively, it may modulate insertion efficiency and inhibit unwanted egress of TMDs (not shown). A single TMD is shown for simplicity, yet the intramembrane space between the EMC and Spf1 is large enough to also accommodate multiple TMDs, which may suggest an action on clients beyond individual TMDs. Our data show that both the EMC and Spf1 retain their activity (shown on the left) in the supercomplex.

Our study shows that the ER of eukaryotic cells contains a previously uncharacterized, high-abundance assembly between two major membrane proteostasis machineries: the EMC, a multifunctional molecular machine hosting a TMD insertase^12,13,21,41^, and Spf1, an ATP-dependent transmembrane dislocase^32,55^. This assembly is present in an apparent 1:1 stoichiometry when isolated at endogenous levels and remains stable over multiple purification steps, indicating a robust interaction. We determined cryo-EM structures of endogenous and recombinant EMC:Spf1 complexes, which together reveal key features of this integrated organization of two proteostasis machineries. Firstly, the TMD insertase cavity of the EMC and the dislocase cleft of Spf1 are juxtaposed in the membrane, creating a spatial arrangement that could facilitate coordination of insertion and extraction events. Secondly, Emc7 and Emc10 together act as a recruitment platform for Spf1, providing a biochemical function for ill-characterized Emc10. Thirdly, nucleotide-state-dependent conformational cycling of Spf1 remodels the interface between the two machineries while preserving their individual structural integrity. Lastly, experimentally validated structural modeling shows that this assembly and its architecture is conserved from yeast to humans. Together, our study suggests a structural framework for how membrane protein insertion and extraction machineries can be spatially coordinated in the ER membrane to support eukaryotic membrane protein insertion and topogenesis (Fig. 6).

### Juxtaposed insertase and dislocase cavities in the EMC:Spf1 assembly

Our structural analyses reveal two adjacent intramembrane substrate engagement sites contributed by EMC and Spf1, which suggests that the biochemical functions of TMD insertion and extraction could be spatially coordinated. Rather than forming a single catalytic cavity, the EMC insertase vestibule and the Spf1 dislocase cleft remain structurally distinct while facing each other within the membrane. This arrangement provides a structural framework for understanding how TMD insertion, quality control and potential proofreading could occur within a shared membrane environment. Both EMC and Spf1 use selectivity filters based on charged residues (Extended Data Fig. 4e). Insertion by EMC is gated at the cytosolic cavity entrance, where positive residues act as a charge-selective barrier, excluding misoriented or mismatched TMDs^21^. Conversely, Spf1 recognizes mislocalized or misoriented substrates that contain similar positively charged motifs exposed to the ER lumen^32^. This shared logic of charge-based discrimination may enable synergistic proofreading on both sides of the ER membrane during membrane protein biogenesis. The close spatial arrangement of EMC and Spf1 may, therefore, increase the efficiency of membrane protein quality control, allowing newly inserted or misassembled TMDs to be rapidly sampled and, if necessary, extracted. Our data suggest that this close spatial coordination may become particularly relevant under conditions that challenge ER proteostasis, such as cellular stress.

An additional notable feature of the EMC:Spf1 complex in yeast is that it appears to induce local bending of the detergent micelle, which is potentially transferrable to a membrane environment. Such membrane deformations may be functionally relevant, as curvature may create a more fluid lipid microenvironment enriched in conical lipids, induce local distortions in the lipid bilayer and better accommodate hydrophobic mismatch. Together, these effects can lower energetic barriers for membrane protein insertion or extraction.

### Spf1 as an ATPase-driven molecular machine in the EMC:Spf1 supercomplex

An important mechanistic implication from our structural investigations of the EMC:Spf1 complex is the dynamic interplay between the ATPase cycle of Spf1 and its interface with the EMC. This provides insights into a key question in the field: Are membrane protein insertases energy-driven and, if so, how is ATP hydrolysis functionally coupled to insertion? Our structures show pronounced conformational changes in the EMC:Spf1 complex that correlate with the ATPase cycle of Spf1 and potentially regulate access to the EMC insertase cavity. Specifically, the unique Spf1 arm domain directly interacts with two cytoplasmic helices of Emc3 and the extension of the first helix of the Emc4 gate in the ATP-bound state. In this conformation, the arm occludes the entrance to the EMC insertase vestibule and the Spf1 dislocase cleft from the cytoplasm. A previous study on *Chaetomium* *thermophilum* Spf1 speculated about a similar function for the arm domain and could resolve dislocation of the arm from the cavity entrance upon ATP hydrolysis, thus functioning as a dynamic plug^36^. On the basis of these collective findings, we propose that this ATP-regulated shielding may function as a gating mechanism for the overall EMC:Spf1 complex and may be important for avoiding reintegration of freshly extracted TMDs and/or support the insertase function of the EMC. How this is coordinated mechanistically and its functional consequences will be important to study in future work.

A particularly intriguing question is what drives ATP hydrolysis and progression in the Post–Albers cycle. Earlier biochemical studies have shown that, in addition to Mg^2+^, certain lipids such as PI4P, ergosterol and ceramide can stimulate ATP turnover^39^; however, what drives the progression from E1-P to E2-P is not well understood^56^.

ATP hydrolysis is a hallmark of high-fidelity molecular machines, often used to provide directionality or proofread critical steps. Our data now show that such an energy-coupled regulation could also be used for controlling protein insertion by the EMC, similar to other energy-dependent insertases such as the GET pathway.

### The EMC is a modular membrane protein quality control hub

In recent years, three distinct complexes of the hEMC have been structurally characterized (Extended Data Fig. 9b), revealing key aspects of its functions in membrane protein biogenesis: (1) the structure of a bound assembly intermediate of a voltage-gated anion channel^23^ provided strong evidence for a holdase function of the EMC^24^; (2) the EMC:BOS holo-insertase complex introduced the concept of the EMC as a central hub in the ER that orchestrates membrane protein biogenesis^10^; and (3) a structure of the EMC bound to endogenous VDAC^57^ raised new questions on further roles of the EMC in interorganelle communication. The EMC:Spf1 structure now adds another distinct mode of interaction in comparison to these hEMC complexes, mediated by a so far largely uncharacterized interaction interface involving the subunits Emc7 and Emc10, which would in principle allow for simultaneous interactions with other partners through other interfaces. This greatly extends the view of the EMC as a central hub for membrane protein quality control.

Protein targeting and insertion mechanisms are promiscuous to accommodate a wide range of clients but this ultimately makes them error-prone, making corrective activities essential. Hence, protein localization is a dynamic process, influenced by a steadily growing network of unique factors that constantly insert (for example, Sec61, GET, EMC, TMCO1, MTCH1/2 and TOM70) or extract (such as Spf1 and Msp1) client proteins from the intracellular membranes. Importantly, these pathways operate within a highly redundant and buffered network, in which multiple machineries can compensate for each other to ensure robustness of membrane protein biogenesis. As a consequence, perturbations of individual components often result in relatively mild phenotypes, as clients can be rerouted or handled by alternative pathways (Supplementary Fig. 1c). We are only beginning to understand the dynamic equilibrium between these distinct molecular machines and manipulation of one player may lead to unexpected outcomes because of client rerouting, compensated or uncompensated imbalances or premature degradation. It will be important to systematically characterize and dissect the interplay between these systems in future work.

Taken together, our results identify the EMC:Spf1 assembly as an evolutionarily conserved interaction between membrane protein insertase and dislocase machines in the ER of eukaryotic cells. It juxtaposes structurally distinct insertion and extraction machineries within a stable assembly while preserving their individual activities. Furthermore, our study reveals that the EMC engages clients and interaction partners through distinct interfaces. It will now be important to analyze how engagement of the different sites is regulated to ultimately govern the fate of diverse membrane protein clients in different organisms.

## Methods

### DNA constructs and molecular biology

DNA for all yeast genes (*Emc1*, *Emc2*, *Emc3*, *Emc4*, *Emc5*, *Emc6*, *Emc7/Sop4*, *Emc10*, *Spf1*, *Om14*, *Cst26*, *Erg9* and *Gnp1*) was isolated from genomic DNA of *S*. *cerevisiae* BY4741 and epitope tags (FLAG, TSII) were introduced by PCR, before cloning into a pLIB vector and ultimately combining all EMC subunits into one expression vector using the biGBac multigene cloning method^58^. DNA for human ATP13A1, hEMC7 and hEMC10 was obtained from the National Institutes of Health Mammalian Gene Collection cDNA library and cloned into respective vectors for expression in mammalian cells (pEG, pCDNA3.4). The pSpCas9(BB)-2A-Puro (PX459) vector was obtained from Addgene (48139). The Pex15Δ30 reporter construct^50^ and the gene for mNeongreen were kind gifts from M. Lemberg (University of Cologne). The rescue vectors for expression in yeast were based on a YCplac33 vector (American Type Culture Collection, 97586) placing the *Spf1* gene between an ADH1 promoter and an ADH1 terminator. All constructs were derived from these using standard molecular biology procedures and Gibson assembly and verified by DNA sequencing. Tables describing all vectors (Supplementary Table 3) and all oligonucleotides (purchased from Sigma-Aldrich; Supplementary Table 4) are included.

### Yeast strains

All strains were based on the BY4741 strain (Euroscarf, Y00000). Standard techniques^59,60^ and the lithium-acetate-based transformation protocol^61^ were used to C-terminally tag Emc5 and Spf1 in *S*. *cerevisiae* for initial IP–MS and structural studies and to N-terminally tag Gem1 and knock out *Emc3* and *Spf1*. Strains were verified by sequencing. For introducing *Emc7* and *Emc10* variants into their endogenous loci, YMS7564 and YMS7565 were used, respectively. After transformation with PCR products encoding the required variants and a Hygromycin (Hygro) cassette, cells were plated on solid agar media containing 5-FOA (5-fluoro-orotic acid monohydrate) for counterselection and Hygro for positive selection. Strains were validated by PCR, followed by genomic sequencing of either the *Emc7* or *Emc10* locus, to ensure that the inserted variants were with their endogenous 5′ UTR regions. A list of all yeast strains used in this study is included in Supplementary Table 5 and all oligonucleotides used for these genetic manipulations are provided in Supplementary Table 4.

### Human cell lines

HEK293T cells (Sigma-Aldrich, 12022001) were cultured in high-glucose DMEM supplemented with 10% (v/v) FBS (Gibco) and 1% (v/v) antibiotic–antimycotic supplement (Gibco; final concentration 100 U ml^−1^ penicillin, 0.1 mg ml^−1^ streptomycin and 2.5 µg ml^−1^ amphotericin B) at 37 °C with 5% CO_2_.

### Detergents

Digitonin (Sigma-Aldrich) was further purified as described previously^62^. In brief, 30 g of digitonin was dissolved in 500 ml of boiling double-distilled water and kept at 4 °C for 36 h before the precipitate was removed by centrifugation at 20,000*g* for 15 min. Then, 10 mM Tris-HCl was added to adjust the pH to 8.0. The solution was finally passed sequentially through Q-Sepharose and S-Sepharose resins by gravity flow and stored as a 5% stock solution at −20 °C. GDN (Anatrace) was dissolved in double-distilled water at 5% (w/v) and stored at −20 °C.

### Isolation of endogenous EMC and Spf1 from *S*. *cerevisiae*

Microsome preparation and IP were performed as described previously^22^. *S*. *cerevisiae* were grown in YPD until late log phase, isolated and resuspended in 1 ml of lysis buffer (50 mM HEPES–KOH pH 7.5, 150 mM potassium acetate, 2 mM magnesium acetate, 1 mM CaCl_2_, 0.2 M sorbitol and 2× complete EDTA-free protease inhibitor (Roche)) per 6-g pellet and frozen dropwise in liquid nitrogen. Cell walls were disrupted by cryomilling using a Spex SamplePrep freezer/mill. The powder was thawed and, after the addition of 1 ml of lysis buffer per g of pellet, nutated at 4 °C for 45 min and dounced in a dounce homogenizer to dissolve in lysis buffer. The lysate was spun down twice at 1,000*g* for 10 min to remove unbroken cells and large fragments before the supernatant was transferred to an ultracentrifuge tube and centrifuged at 50,000*g* for 20 min to pellet membranes. The microsomes were resuspended in a small volume of lysis buffer and flash-frozen.

Next, 15 ml of IP buffer (50 mM HEPES–KOH pH 7.5, 150 mM potassium acetate, 2 mM magnesium acetate, 1 mM CaCl_2_, 15% (v/v) glycerol and 1× complete EDTA-free protease inhibitor) containing 2% (w/v) digitonin was added and the microsomes were nutated for at least 1 h at 4 °C to solubilize. Insoluble material was removed by centrifugation at 50,000*g* and the supernatant was added to FLAG M2 agarose beads (EMC) or StrepTactin agarose (IBA) for affinity isolation for 90 min. The resin was washed extensively before elution with 0.15 mg ml^−1^ 3×FLAG peptide or 5 mM D-desthiobiotin. For interactomics, bound protein was not eluted but directly digested on the beads, as detailed later.

### Purification of endogenous EMC for cryo-EM

The large-scale purification of endogenously tagged EMC was performed analogously to the procedure described above. To obtain a high biomass, a yeast strain containing Emc5–3×FLAG and Spf1–TSII was grown in a fermenter to high cell density before harvesting, dropwise freezing and cryomilling. Then, 700 g of milled powder was used as input and a FLAG pulldown was performed as described above. Elution fractions were combined and concentrated before injection onto a Superose 6 column and SEC in SEC buffer (20 mM HEPES pH 7.5, 150 mM potassium acetate and 0.03% (w/v) digitonin). Fractions were analyzed by SDS–PAGE, pooled and concentrated before plunging.

### Expression and purification of recombinant EMC:Spf1 from insect cells

Bacmids were produced in DH10EMBacY *Escherichia* *coli* for subsequent transfection into Sf9 insect cells (Thermo Fisher, B85502) to generate baculovirus. Hi5 insect cells (BTI-TN-5B1-4; Thermo Fisher, 11496016) were coinfected with respective baculoviruses and protein expression was carried out for 3 days. Cells were harvested, washed in ice-cold PBS and lysed in hypotonic lysis buffer (10 mM HEPES pH 7.5 and 1 mM MgCl_2_) supplemented with complete EDTA-free protease inhibitor (Roche) and 0.001 mg ml^−1^ Benzonase. The membrane fraction was pelleted by centrifugation at 50,000*g* for 30 min and resuspended by dounce homogenization in IP buffer (50 mM HEPES–KOH pH 7.5, 150 mM potassium acetate, 2 mM magnesium acetate, 1 mM CaCl_2_ and 15% (v/v) glycerol). Membranes were solubilized by adding purified digitonin to a final concentration of 1.5% (w/v) and rotated for 3 h. Insoluble material was removed by centrifugation at 50,000*g* for 30 min and the supernatant was incubated under agitation with StrepTactin agarose resin (IBA Lifesciences) to capture the Emc5–TSII complex. After washing with a total of 20 column volumes of IP buffer with 0.1% (w/v) digitonin, bound protein was eluted with IP buffer containing 5 mM D-desthiobiotin and 0.1% (w/v) digitonin. Elution fractions were analyzed by SDS–PAGE for protein content, then pooled and incubated with anti-FLAG M2 resin (Sigma) overnight. Beads were washed with 20 column volumes of IP buffer with 0.1% (w/v) digitonin and bound EMC:Spf1 complexes were eluted with IP buffer supplemented with 0.15 mg ml^−1^ 3×FLAG peptide and 0.1% (w/v) digitonin. Protein containing elution fractions were pooled and concentrated to 10 mg ml^−1^ and subjected to SEC on a Superose 6 column in SEC buffer (20 mM HEPES pH 7.5, 150 mM potassium acetate and 0.03% (w/v) digitonin). All fractions were analyzed by SDS–PAGE and respective fractions were pooled and concentrated by centrifugation.

### Nanodisc reconstitution

EMC:Spf1 from insect cells following Strep and FLAG affinity purification was mixed with MSP2N2 (obtained from the MPIB biochemistry core facility) and yeast polar lipid extract (Avanti Polar Lipids) at a concentration of 1 µM for EMC:Spf1 and indicated molar ratios for the nanodisc scaffold and lipids and incubated for 2 h on ice. Nanodisc reconstitution was achieved by adding BioBeads SM-2 (Bio-Rad) for 16 h at 4 °C under constant agitation. The samples were removed from the BioBeads, filtered and separated on a Superose 6 5/150 GL column at a flow rate of 0.1 ml min^−1^ in a buffer containing 20 mM Tris pH 7.5, 100 mM NaCl and 1 mM MgCl_2_. Indicated fractions were analyzed by SDS–PAGE.

### MS

#### Experimental design

All samples were prepared in triplicate. IPs from yeast were performed as described above for the isolation of the endogenous EMC for cryo-EM. IPs from mammalian cells were performed as described. To exclude clonal effects, triplicates of three different endogenously tagged HEK293T clones were compared to untagged HEK293T cells.

#### Sample preparation

The beads were washed 1× with PBS, the buffer was removed and the beads were incubated for 20 min at 37 °C with 100 µl of SDC buffer (1% sodium deoxycholate (Sigma-Aldrich), 40 mM 2-chloroacetamide (Sigma-Aldrich) and 10 mM TCEP (Thermo Fisher Scientific) in 100 mM Tris, pH 8.0). Then, the samples were diluted with 100 µl of milliQ water and the proteins were digested overnight at 37 °C by addition of 0.5 µg of trypsin (Promega). The supernatant was collected with the help of a magnetic rack and was acidified with trifluoroacetic acid (Merck) to a final concentration of 1%. Precipitated SDC was removed by centrifugation and the peptide mixture was desalted using SCX StageTips. After elution, the samples were vacuum-dried and dissolved in 15 µl of buffer A (0.1% formic acid).

#### LC–MS/MS data acquisition

LC–MS/MS data acquisition was performed using an Easy nanoLC coupled to a Q Exactive HF MS instrument (Thermo Fisher) or a timsTOF Pro MS instrument (Bruker Daltonic). Peptides (3 µl, corresponding to 500 or 200 ng) were loaded onto a 30-cm column (inner diameter: 75 μm; packed in-house with ReproSil-Pur C18-AQ 1.9-μm beads, Dr. Maisch) using the autosampler of the Thermo Easy-nLC 1200 (Thermo Fisher Scientific). The column was heated to 60 °C. Peptides were separated with a flow rate of 250 nl min^−1^ by a gradient of buffer B (80% acetonitrile, 0.1% formic acid) from 2% to 30% B and then to 60% B over 10 min, to 95% B over the next 5 min and maintained at 95% B for another 5 min.

The Q Exactive HF MS instrument was operated in a data-dependent mode with MS1 scans from 300 to 1,750 *m*/*z* (resolution of 60,000 at *m*/*z* 200) and up to 15 of the top precursors were selected for fragmentation using higher-energy collisional dissociation (normalized collision energy = 28). The MS2 spectra were recorded at a resolution of 15,000 (at *m*/*z* 200). The automatic gain control target for MS1 and MS2 scans were set to 3 × 10^6^ and 1 × 10^5^, respectively, within a maximum injection time of 100 and 25 ms for MS1 and MS2 scans, respectively.

Data acquisition on the timsTOF Pro was performed using OtofControl 6.0. The MS instrument was operated in data-dependent parallel accumulation–serial accumulation (PASEF) mode with one survey TIMS-MS and ten PASEF MS/MS scans per acquisition cycle. Analysis was performed in a mass scan range from 100–1,700 *m*/*z* and an ion mobility range from 1/*K*_0_ = 1.6 V s cm^−2^ to 0.6 V s cm^−2^ using equal ion accumulation and a ramp time in the dual TIMS analyzer of 100 ms each at a spectrum rate of 9.52 Hz. Suitable precursor ions for MS/MS analysis were isolated in a window of 2 Th for *m*/*z* < 700 and 3 Th for *m*/*z* > 700 by rapidly switching the quadrupole position in sync with the elution of precursors from the TIMS device. The collision energy was lowered stepwise as a function of increasing ion mobility, starting from 52 eV for 0–19% of the TIMS ramp time, 47 eV from 19% to 38%, 42 eV from 38% to 57%, 37 eV from 57% to 76% and 32 eV to the end. We made use of the *m*/*z* and ion mobility information to exclude singly charged precursor ions with a polygon filter mask and further used ‘dynamic exclusion’ to avoid resequencing of precursors that reached a ‘target value’ of 20,000 a.u. The ion mobility dimension was calibrated linearly using three ions from the Agilent ESI LC–MS tuning mix (1/*K*_0_: *m*/*z* 622.0289, 0.9848 V s cm^−2^; *m*/*z* 922.0097, 1.1895 V s cm^−2^; *m*/*z* 1,221.9906, 1.3820 V s cm^−2^).

#### Data analysis

Raw data were processed using the MaxQuant computational platform (version 2.0.1.0)^63^ with standard settings applied. Shortly, the peak list was searched against the UniProt database of *S*. *cerevisiae* or *Homo* *sapiens* and the bait protein. Cysteine carbamidomethylation was set as static modification and methionine oxidation and N-terminal acetylation as variable modifications. The match-between-run option was enabled and proteins were quantified across samples using the label-free quantification algorithm in MaxQuant generating label-free quantification (LFQ) intensities.

MaxQuant outputs were imported into Perseus (version 1.6.15.0)^64^. Potential contaminants, reverse-identified hits and proteins only identified by site were removed by filtering before LFQ intensities were log_2_-transformed, rows with fewer than three valid values in at least one group were removed before missing values were imputed from a downshifted normal distribution. Samples were compared by two sample Student’s *t*-tests. Classifications shown in Supplementary Fig. 1a,b were derived from the available UniProt annotations and Gene Ontology terms.

### Single-particle cryo-EM

#### Sample preparation

The EMC:Spf1 apo sample was concentrated to 5.0 mg ml^−1^, the endogenous yeast EMC sample was concentrated to 1.4 mg ml^−1^. For the EMC:Spf1^ATPγS^ sample, the purified protein was concentrated to 4.5 mg ml^−1^ and preincubated with 2 mM ATPγS (Jena Bioscience) for 30 min on ice. For the EMC:Spf1^AlF4−^ sample, the purified protein was concentrated to 4.5 mg ml^−1^ and preincubated with 1.7 mM ADP and 10 mM NaF for 10 min on ice, before adding 2 mM of AlF_3_ and incubating for 1 h on ice. All samples were passed through a centrifugal filter to remove potential aggregates.

Next, 3.6 µl of freshly purified, concentrated sample was applied on glow-discharged R1.2/1.3 300-mesh holey carbon grids (Quantifoil) and plunge-frozen in liquid ethane on a Vitrobot Mark IV (Thermo Fisher; blot force of 4, blot time of 3 s, 4 °C and 100% humidity).

#### Data collection

The endogenous yeast EMC grids were screened and data were collected using SerialEM (version 3.8)^65^ on a Glacios cryo-TEM instrument (FEI) operated at 200 kV and equipped with a K2 Summit direct electron detector (Gatan). A total of 3,690 micrographs were collected at ×22,000 magnification, corresponding to a pixel size of 1.885 Å, in a defocus range of −1.0 to −2.6 µm, with a total dose of 60 e^−^ per Å^2^ fractionated over 40 frames.

All grids of the recombinant EMC:Spf1 sample were screened using SerialEM (version 4.1)^65^ on a Talos Arctica cryo-TEM instrument (FEI) operated at 200 kV and equipped with a K3 direct electron detector (Gatan). Screening datasets and the final dataset for EMC:Spf1^AlF4−^ were collected at ×22,000 magnification, corresponding to a pixel size of 1.841 Å, in a defocus range of −1.0 to −2.6 µm, with a total dose of 60 e^−^ per Å^2^ fractionated over 40 frames.

High-resolution datasets of the recombinant EMC:Spf1 in apo state and after incubation with ATPγS were collected using SerialEM (version 4.1)^65^ on a Titan Krios cryo-TEM instrument (FEI) operated at 300 kV, equipped with a K3 direct electron detector (Gatan) in counting mode and a Bio Quantum-LS postcolumn energy filter (Gatan; 10 eV). The datasets were recorded at ×105,000 magnification, corresponding to a pixel size of 0.8222 Å, in a defocus range of −0.7 to −2.2 µm, with a total dose of 65.1 e^−^ per Å^2^ (apo) and 62.62 e^−^ per Å^2^ (ATPγS).

#### Data processing

##### Recombinant EMC:Spf1 complex (Supplementary Fig. 2)

Following import into RELION (version 5.0)^66,67^, the micrographs underwent motion correction and dose-weighting, leveraging RELION’s built-in MotionCor2 implementation. Contrast transfer function (CTF) estimation was then performed with CTFFIND (version 4.1.9)^68^.

For the apo dataset, we initially picked 3,817,633 particles using Gautomatch (http://www.mrc-lmb.cam.ac.uk/kzhang/). These were then subjected to three-dimensional (3D) classification on a binned dataset (pixel size: 3.05 Å) with a regularization parameter of *T* = 16, yielding 924,465 particles for further 3D classification. After multiple rounds of 3D classification and orientation refinements, the particle count was narrowed to 645,091. These particles were then extracted to full pixel size for additional 3D refinements. We used RELION’s multibody refinement implementation to resolve the EMC and Spf1 components. Separate postprocessing of these two bodies resulted in resolutions of 3.8 Å for EMC and 4.2 Å for Spf1. Following 3D refinement, the final map was post-processed in RELION, and the pixel size was recalibrated to 0.8222 Å pixel^−1^ to ensure the correct physical scale of the reconstructed map. We used DeepEMhancer^69^ to sharpen the maps and a composite map was constructed using PHENIX^70^ with the DeepEMhancer-sharpened EMC and Spf1 maps as input.

##### Endogenous yeast EMC (Supplementary Fig. 3)

Raw videos were processed in cryoSPARC (version 4.7.1)^71^ using motion correction and patch CTF estimation and manually curated, excluding 429 exposures. Templates for particle picking were generated on the basis of manual picking and initial 3D model generation and resulted in 814,288 particles that were extracted at a binned size (pixel size: 6.7 Å). These were subjected to one round of two-dimensional (2D) classification and one round of heterogeneous refinement based on the initial 3D models. The handedness of the best class was flipped before subjecting it to one round of nonuniform refinement. The particles were extracted at full pixel size (1.885 Å) and underwent further refinement before being used to train a 3D Flex model^72^ with four latent modes. A total of 80,000 particles were used to reconstruct the flexible volume, which was further refined after reference-based motion correction, resulting in a consensus map of 8.7-Å resolution. A mask around the EMC allowed to locally refine the EMC to 8.6-Å resolution, resolving all its transmembrane helices.

##### EMC:Spf1^ATPγS^ (Supplementary Fig. 10)

Following import into RELION (version 5.0), the micrographs underwent motion correction and dose-weighting, using RELION’s built-in MotionCor2 implementation. CTF estimation was then performed with CTFFIND (version 4.1.9).

For the ATPγS dataset, 2,275,267 particles were initially picked using Gautomatch. These were subjected to 3D classification on a binned dataset (pixel size: 3.05 Å) with a regularization parameter of *T* = 16, yielding 514,084 particles. Multiple rounds of classification and refinement were carried out to discard poor-quality particles, resulting in a final subset of 33,661 high-quality particles. These particles were reextracted at full pixel size for subsequent refinement and postprocessing, ultimately yielding a 3.8-Å reconstruction. Following 3D refinement, the final map was post-processed in RELION, and the pixel size was recalibrated to 0.8222 Å pixel^−1^ to ensure the correct physical scale of the reconstructed map.

##### EMC:Spf1^AlF4−^ (Supplementary Fig. 11)

Raw videos were processed in cryoSPARC (version 4.7.1)^71^ using motion correction and patch CTF estimation. Particles were first picked using the blob picker tool to create an initial model for 2D template generation and template-based picking of 2,059,914 particles. After two rounds of 2D classification, 228,528 particles were used to generate initial 3D models, which served as input models for two rounds of heterogeneous refinement of the full particle set. The best class was refined once, extracted at full pixel size (1.885 Å) and subjected to two further rounds of refinement. After an additional round of 3D classification, the best class, consisting of 103,225 particles, was refined and postprocessed, resulting in a final global resolution of 6.8 Å. DeepEMhancer^69^ was used to further sharpen the final map.

#### Model building

The 3.8-Å reconstruction map of the ATPγS-bound complex revealed well-defined main-chain and side-chain densities, enabling the fitting of a previously determined structure for EMC (PDB 7KRA) and a model for Spf1 predicted by AlphaFold^73,74^ (AF-P39986-F1). Initial docking was performed using UCSF Chimera^75^, allowing each domain to move independently during rigid-body refinement in PHENIX^76^. Model building and real-space refinement were carried out iteratively to optimize the fit. Manual adjustments were made in Coot^77^ and real-space refinements were conducted using phenix.real_space_refine.

Models for the 3.8-Å EMC and the 4.2-Å Spf1 apo structure were built using the same approach. Because of limited resolution in the nucleotide-binding domains, side-chain positioning was often ambiguous; in such cases, rotamers from previously published structures were retained when appropriate. Density for a nucleotide analog was observed at the respective sites (Extended Data Fig. 8a); however, it was not sufficient for precise modeling of such. DeepEMhancer-sharpened and consensus refinement maps were used to guide manual model building in Coot. Real-space refinements in PHENIX were conducted against postprocessed maps from RELION.

#### Figure preparation

All structural figures were prepared using ChimeraX (version 1.9)^78^. Schematics were drawn using Adobe Illustrator.

### AlphaFold structure prediction

The structural model for the hEMC–ATP13A1 complex was predicted using AlphaFold 3 (ref. ^53^) on the AlphaFold Server. Protein sequences for hEMC subunits 1, 2, 3, 4, 5, 6, 7, 8 and 10 and ATP13A1 were derived from UniProt using the most common isoform; if applicable, the signal peptide was removed and the following post-translational modifications were added, as annotated in the UniProt database: hEMC1:N348 *N*-acetyl-β-D-glucosamine, hEMC1:N796 *N*-acetyl-β-D-glucosamine, hEMC1:N891 *N*-acetyl-β-D-glucosamine, hEMC2:K254 *N*^6^-acetyl-L-lysine, hEMC4:T1 phosphothreonine, hEMC10:N157 *N*-acetyl-β-D-glucosamine, ATP13A1:N286 *N*-acetyl-β-D-glucosamine, ATP13A1:N419 N-acetyl-β-D-glucosamine, ATP13A1:S898 phosphoserine and ATP13A1:S904 phosphoserine.

The AlphaFold model for the yeast EMC:Spf1 complex was predicted using the UniProt protein sequences for the EMC subunits and Spf1. If applicable, the signal peptide was removed and glycosylations were added as observed in our structure.

### Co-IPs from *S*. *cerevisiae*

All yeast strains were grown on agar plates containing the respective selection markers. Single colonies were used to inoculate overnight cultures in selection medium. Cultures were diluted to an optical density at 600 nm (OD_600_) of 0.2 in YPD medium and grown until mid-log phase was reached. Then, 80 OD_600_ units were collected by centrifugation and cells were washed with H_2_O before flash-freezing. The pellet was resuspended in 1× IP buffer with 1% (w/v) purified digitonin and complete EDTA-free protease inhibitor (Roche) and cell lysis was carried out in a FastPrep-24 benchtop homogenizer system (MP Biomedicals) using the lysing matrix C (1-mm silica spheres). The supernatant was collected after centrifugation at 16,000*g* for 10 min and incubated with anti-GFP magnetic agarose (Chromotek) for 2 h under agitation. Beads were washed with IP buffer with 0.1% (w/v) digitonin and bound protein was eluted by addition of 2× Lämmli buffer with 10% (v/v) β-mercaptoethanol and incubation at 60 °C for 10 min.

### Co-IPs from insect cells

Spf1 mutants and wt yeast EMC were coexpressed in Hi5 insect cells by baculovirus coinfection as described above. Cells were harvested 3 days after infection; pellets were washed with ice-cold PBS and lysed in 1× IP buffer supplemented with 1% (w/v) digitonin and 20 µg ml^−1^ aprotinin, 10 µg ml^−1^ leupeptin, 10 µg ml^−1^ pepstatin and 1 µg ml^−1^ Benzonase. Membranes were solubilized for 90 min before removing insoluble material by centrifugation at 20,000*g* for 30 min. The soluble material was incubated with magnetic StrepTactin resin (PureCube HiCap StrepTactin MagBeads, Cube Biotech) for 90 min under constant agitation. The beads were washed five times with 1× IP buffer supplemented with 0.01% (w/v) digitonin. Co-IP proteins were eluted by heating the beads in 2× Lämmli buffer with 10% (v/v) β-mercaptoethanol at 60 °C for 10 min.

### Flow cytometry and data analysis

Yeast strains were cotransformed with one plasmid containing an Spf1 variant or empty vector and one plasmid encoding for the protein of interest fused to the mCherry–sfGFP timer, as described previously^50^. Transformants were plated on a plate containing the respective selection markers and positive clones were isolated and grown in selection medium overnight. The cultures were diluted to an OD_600_ of 0.2 in SDC medium and grown until mid-log phase before analysis on an AttuneNxt Flow Cytometer (Thermo Fisher). To induce the unfolded protein response, cells were treated with 2 mM DTT for 3 h after reaching mid-log phase. For terbinafine treatment, overnight cultures were directly diluted into SDC medium containing 5 µg ml^−1^ terbinafine hydrochloride.

The acquired data were analyzed in FlowJo10 (BD Life Science). The gating strategy (visualized in Supplementary Fig. 12a) involved selecting single cells according to behavior in forward and sideward scatter, excluding duplicate and autofluorescent events and gating for sfGFP-positive cells. The mCherry-to-sfGFP ratio was derived from the respective signal heights. The mean mCherry-to-sfGFP ratio was calculated and normalized to the untreated control condition (*ho::G418* transformed with an empty vector containing the Ura3 selection marker and the respective reporter construct). Statistical analyses were performed using GraphPad Prism 10.

### Microscopy

Cells were grown in selection medium overnight, diluted to an OD_600_ of 0.2 in YPD and grown until mid-log phase. Cells were washed once and stained with 250 nM Mitotracker Red CMXRos for 30 min at 30 °C, washed twice and resuspended in YPD. Subsequently, cells were adhered to an eight-well glass bottom µ-slide coated with concanavalin A (ibidi) for imaging.

Images were obtained using a Leica DMi8 inverted widefield microscope with a DFC9000GTC camera (Leica) controlled by LasX Software. An HCX PL APO ×100/1.47 oil objective was used together with a QUAD-P-T dichroic mirror using the range for GFP (excitation: 484–496 nm, emission: 502–538 nm) and mCherry (excitation: 557–567 nm, emission: 584–616 nm) and a 488-nm (152 mW) or a 561-nm (124 mW) laser, respectively. All images were acquired as *z* stacks with regular spacing and deconvoluted using the Leica THUNDER small volume deconvolution module. Further processing was performed in ImageJ Fiji^79^ by selecting a single focal plane from the z-stack, performing background correction and adjusting brightness to the same values for each image.

### Endogenous tagging of MMGT1 (hEMC5) with mNeongreen by CRISPR–Cas9-mediated genome editing

#### Guide design and construction of the homology-directed repair (HDR) template

Two single guide (sg)RNAs were designed against the C terminus of the *MMGT1* gene, targeting the sense and antisense DNA strand of exon 4 before and after the endogenous stop codon: guide 1, CACCGTTTTTACAAATTATAATAAT; guide 2, CACCGTAACTTCAATGATGTGTTAG.

The guides were cloned individually into the pSpCas9(BB) vector as described previously^80^ and a dual expression cassette vector was constructed by linearization by restriction digest with XbaI and insertion of the second sgRNA expression cassette by Gibson assembly. The HDR template was constructed by amplifying 500-bp 3′ and 600-bp 5′ homology arms from the endogenous *MMGT1* locus adjacent to the guide cutting sites. The protospacer-adjacent motifs were mutated to prevent recognition by the sgRNA. A 15-aa linker and the gene for mNeongreen were inserted between the two flanking regions. The construct was amplified by PCR, analyzed on an agarose gel and purified using a silica-membrane-based spin column (QIAquick PCR purification kit, Qiagen).

#### Transfection, antibiotic selection and fluorescence-activated cell sorting of single-cell clones

HEK293T cells were seeded 24 h before transfection with the plasmid containing the dual guide and Cas9 and a fourfold molar excess of the purified HDR template using Lipofectamine 2000 (Thermo Fisher) as the transfection reagent according to the manufacturer’s instructions. Selection for transfected cells with 1 µg ml^−1^ puromycin (Gibco) was carried out 48 h after transfection for 96 h, replacing the antibiotic-containing medium every 24 h. Selected cells were allowed to recover and proliferate for 5 days before the top 0.9% mNeongreen-positive single-cell clones were isolated by fluorescence-activated cell sorting on a CytoFLEX SRT instrument (Beckman Coulter).

#### Clonal validation

Individual clones were expanded and analyzed for the presence of the genomic insertion on both alleles by PCR-based screening and for the expression of the EMC5–mNeongreen fusion protein by western blot. Positive clones were further validated by deep sequencing of the insert and flanking genomic regions.

### Transient transfections

For transient transfections, 600,000 HEK293T cells were seeded in a p35 well 24 h before transfection. Next, 1 µg of DNA per construct was transfected using Metafectene Pro (Biontex) according to the manufacturer’s instructions. Protein expression was analyzed 24 h after transfection.

### In-cell cysteine crosslinking

Cells for this experiment were cultivated in poly(D-lysine)-coated dishes. Before crosslinking, cells were washed briefly with PBS before adding 250 µM BMOE (Thermo Fisher, dissolved in anhydrous DMSO to 20 mM) in PBS with 5 mM EDTA. Crosslinking was performed at room temperature in the dark for 1 h before quenching of the reaction with 20 mM DTT in PBS for 10 min. All subsequent lysis and IP steps were performed on ice as described below, except that 1× RIPA buffer (50 mM Tris-HCl pH 7.5, 150 mM NaCl, 1% (v/v) NP-40, 0.5% (w/v) deoxycholate and 0.1% (w/v) SDS) was used for lysis and washing.

### Co-IPs from HEK293T cells

Cells were briefly washed twice with ice-cold PBS before adding lysis buffer (50 mM Tris-HCl pH 7.5, 150 mM NaCl, 1× complete protease inhibitor EDTA-free (Roche) and 0.5% (w/v) GDN). Membranes were solubilized for 20 min on ice before centrifugation at 15,000*g* for 15 min to remove insoluble material. A small fraction of the lysate was kept as input sample and supplemented with 5× Lämmli buffer with 10% (v/v) β-mercaptoethanol to a final concentration of 1× and denatured at 60 °C for 10 min. The remaining lysate was added to precoupled magnetic beads (anti-FLAG M2 magnetic beads, Millipore; Pierce anti-HA magnetic beads, Thermo Fisher; MagStrep StrepTactin XT beads, IBA) and incubated for 90 min. Unbound material was removed in three subsequent wash steps using wash buffer (50 mM Tris-HCl pH 7.5, 150 mM NaCl and 0.01% (w/v) GDN). For western blot analysis, immunoprecipitated protein was eluted by denaturation using 2× Lämmli buffer supplemented with 10% (v/v) β-mercaptoethanol and heating of samples to 60 °C for 10 min.

### SDS–PAGE, western blots and quantifications

Protein samples were separated on self-made 4–22% or 12% Tris–glycine SDS–PAGE gels. For total protein analysis, gels were stained with Coomassie brilliant blue stain (homemade) or Oriole fluorescent gel stain (Bio-Rad). SDS–PAGE gel band intensities were quantified using the Fiji distribution of ImageJ^79^. In brief, the intensity profile of the gel line was plotted after performing background correction. Peaks were identified using the first derivative of the intensity signal and their intensity was quantified by integrating the area under the curve.

For western blotting, samples were transferred onto a PVDF membrane (Bio-Rad) after SDS–PAGE. Membranes were blocked in 5% (w/v) skim milk powder in Tris-buffered saline with 0.05% (v/v) Tween-20 before probing with primary antibodies diluted in blocking buffer overnight. Membranes were washed, incubated with secondary horseradish peroxidase (HRP)-coupled antibodies for 1 h at room temperature and washed before probing with Amersham ECL Prime detection reagent (Cytiva) and chemiluminescence detection on a Fusion FX Edge (Vilber-Lourmat) or Amersham 600 (GE) imager. Densitometric quantification of western blots was performed with ImageJ Fiji using standard settings and statistical analyses were performed using GraphPad Prism version 10.

Antibodies used in this study were as follows: ATP13A1 (Proteintech, 16244-1-AP), EMC1 (Novus Biologicals, NBP2-59097 and NBP3-18427), EMC4 (Abcam, ab184544), EMC7 (Proteintech, 27550-1-AP), HA.11 (Biolegend, Poly9023), c-myc (Proteintech, 16286-1-AP), FLAG (Proteintech, 20543-1-AP), NWSHPQFEK 5A9F9 (Genscript, A01732), GFP (Proteintech, 50430-2-AP), Hsc70 (Santa Cruz Biotechnology, sc-7298), GAPDH–HRP (Proteintech, HRP-60004), anti-rabbit IgG–HRP (Santa Cruz Biotechnology, sc-2357) and m-IgGκ BP–HRP, sc-516102).

### Statistics and reproducibility

No statistical method was used to predetermine sample size; sample sizes were selected to ensure clear and reliable interpretation of the results. The experiments were not randomized and the investigators were not blinded to allocation during experiments and outcome assessment. No data were excluded from the analyses. Biochemical and cell biological experiments were repeated at least three times in true replicates with similar results; the exact number of replicates (*n*) is specified in the figure legends. A two-tailed Student’s *t*-test was used for pairwise comparison. Whenever possible, multiple clones for the same genetic manipulation of a yeast strain or human cell line were assessed for similar results. Cryo-EM data collection and processing followed standard practices and representative micrographs of each dataset are shown.

### Reporting summary

Further information on research design is available in the Nature Portfolio Reporting Summary linked to this article.

## Online content

Any methods, additional references, Nature Portfolio reporting summaries, source data, extended data, supplementary information, acknowledgements, peer review information; details of author contributions and competing interests; and statements of data and code availability are available at https://doi.org/10.1038/s41594-026-01883-8.

## Supplementary information

Supplementary Information Supplementary Tables 1–5, Figs. 1–12 and uncropped scans of gels and western blots shown in the supplementary figures.

Reporting Summary

Peer Review File

Supplementary Table 1 Interactomes of the EMC (Emc5–3×FLAG) and Spf1 (Spf1–TSII) from *S*. *cerevisiae*.

Supplementary Table 2 Interactome of the hEMC (hEMC5–mNeongreen) in HEK293T cells.

Supplementary Table 4 Oligonucleotides used in this study.

## Source data

Source Data Figs. 1 and 2 and Extended Data Figs. 1, 2 and 5 Unprocessed gels.

Source Data Fig. 3 Numerical source data.

Source Data Fig. 4 Numerical source data.

Source Data Fig. 5 Numerical source data.

Source Data Extended Data Fig. 1 Numerical source data.

Source Data Extended Data Fig. 5 Numerical source data.

Source Data Extended Data Fig. 6 Numerical source data.

Source Data Extended Data Fig. 8 Numerical source data.

## Data Availability

Structural data are available from the EM Data Bank and Protein Data Bank under the following identifiers: composite map of the EMC:Spf1 complex, EMD-59745 and PDB 34BQ (consensus map: EMD-59738, Spf1 component: EMD-59736, EMC component: EMD-59737); EMC:Spf1^ATPγS^, EMD-59746 and PDB 34BR; endogenous EMC:Spf1, EMD-54652 (locally refined endogenous EMC: EMD-54648); EMC:Spf1^AlF4−^, EMD-54635. Proteomics data were deposited to the ProteomeXchange repository under the project accession number PXD067089. Source data are provided with this paper.
