# Supplementary material for: Structural basis of an endoplasmic reticulum EMC:Spf1 insertase–dislocase complex
Source: Nat Struct Mol Biol. 2026 Oct 13;33(10):1548–60. doi: 10.1038/s41594-026-01883-8 (PMC13672079; doi:10.1038/s41594-026-01883-8)

nature structural & molecular biology

Supplementary information https://doi.org/10.1038/s41594-026-01883-8

Structural basis of an endoplasmic reticulum EMC:Spf1 insertase–dislocase complex

In the format provided by the authors and unedited

Supplementary Information

Table of Contents

Supplementary Tables 2

Supplementary Table 3. Plasmids used in this study 3
Supplementary Table 5. Yeast strains used in this study 5

Supplementary Figures 6

SI Fig. 1. Classification of endogenous EMC and Spf1 interactors as well as common interaction partners 7
SI Fig. 2. Cryo-EM data processing of the recombinant EMC:Spf1^{apo} complex 8
SI Fig. 3. Cryo-EM data processing of the endogenous EMC from *S. cerevisiae* in complex with Spf1 9
SI Fig. 4. Reconstitution of recombinant EMC:Spf1 into MSP2N2 lipid nanodisc 10
SI Fig. 5. Fit of the model to the cryo-EM maps 11
SI Fig. 6. AlphaFold 3 prediction of the hEMC:ATP13A1 complex 12
SI Fig. 7. AlphaFold 3 prediction of the yeast EMC:Spf1 complex 14
SI Fig. 8. Sequence alignments of the human and yeast orthologs for Emc7, Emc10 and ATP13A1/ Spf1 15
SI Fig. 9. Structural comparison of the human and yeast models for the proteins Emc7, Emc10 and ATP13A1/ Spf1 17
SI Fig. 10. Cryo-EM data processing of the EMC:Spf1$^{ATP\gamma S}$ complex 18
SI Fig. 11. Cryo-EM data processing of the EMC:Spf1^{AlF4-} complex 19
SI Fig. 12. Representative dot plots and histograms to exemplify the gating strategy applied to all flow cytometry data 20

Uncropped scans of gels and western blots from the SI 21

Supplementary Tables

Supplementary Table 3. Plasmids used in this study

Identifier	description
BBV-534	pLIB-yEMC1
BBV-535	pLIB-yEMC2
BBV-536	pLIB-yEMC3
BBV-537	pLIB-yEMC4
BBV-538	pLIB-yEMC6
BBV-539	pLIB-Sop4
BBV-540	pLIB-yEMC10
BBV-544	pLIB-yEMC5-TSII
BBV-855	pLIB-Spf1-FLAG
pCK-425	pLIB-SPF1-FLAG-R866E W867A N868D Q869E
pCK-426	pLIB-SPF1-FLAG-E1087A
pCK-436	pLIB-SPF1-FLAG-F1154A L1155A
pCK-467	Yplac33-ADH1promoter-Spf1-Flag-ADH1terminator
pCK-470	Yplac33-ADH1promoter-Spf1-Flag-R866E W867A N868D Q869E-ADH1terminator
pCK-471	Yplac33-ADH1promoter-Spf1-Flag-E1087A-ADH1terminator
pCK-472	Yplac33-ADH1promoter-Spf1-Flag-D487A-ADH1terminator
pCK-473	Yplac33-ADH1promoter-empty-ADH1terminator
pVD-48	p415-TEFpr-sfGFP-mCherry-Pex15Δ30
pCK-480	p415-TEFpr-sfGFP-mCherry-Om14
pCK-481	p415-TEFpr-sfGFP-mCherry-Erg9
pCK-484	p415-TEFpr-sfGFP-mCherry-Gnp1
pCK-504	p415-TEFpr-sfGFP-mCherry-Cst26
pCK-176	HDR template for EMC5-15aalinker-mNeongreen endogenous tagging
pCK-168	pX459-dualguide-EMC5-Cterminus
pCK-200	pEG-kozak-SS-FLAG-EMC10
pCK-201	pEG-kozak-SS-FLAG-EMC10 Y230C
pCK-203	pEG-kozak-SS-FLAG-EMC10 L238C
pIB-12b	pEG-kozak-SS-FLAG-EMC10 E172G F176A E178A R179A E181G E183A Q184G Q186G K187A K189G N190A Q192A E193A Q194G K195A
pCK-195	pEG-kozak-SS-HA-EMC7
pCK-196	pEG-kozak-SS-HA-EMC7 M168C
pCK-197	pEG-kozak-SS-HA-EMC7 P172C
pIB-10	pEG-kozak-SS-HA-EMC7-Y125A K127A T128A S129A E130A R133A Y135A
pIB-25	pEG-kozak-SS-HA-EMC7ΔTMD
pIB-11	pEG-kozak-SS-HA-EMC7-W155A W157A T158A D159A F160A
pIB-59	pEG-kozak-SS-HA-EMC7-M162A M167A L171A L174A I175A L178A
pIB-60	pEG-kozak-SS-HA-EMC7-K181A V182A N184A S186A D187A
pIB-61	pEG-kozak-SS-HA-EMC7-D189A M190A E193A Q196A S197A M200A
pIB-21	pCDNA3.4-ATP13A1-TSII
pIB-14	pCDNA3.4-ATP13A1-TSII-E1081A Q1083A E1084A Q1085A
pIB-70	pCDNA3.4-ATP13A1-TSII-F1014A D1154A Q1158A
pMS-249	pYM-NATR-TEFpr-GFP
pMS-555	pYM URA3-NOP1pr-GFP
pMS-1217	pFA6 G418R-MX6
pMS-1695	Myc-Emc7(WT) in pMS108 backbone -HygroR
pMS-1696	Myc-Emc10(WT) in pMS108 backbone -HygroR
pMS-1699	Myc-Emc7-ΔC(I160-K216) in pMS108 backbone -HygroR
pMS-1700	Myc-Emc7(T120A, Y121A) in pMS108 backbone -HygroR

pMS-1703	Myc-Emc10-ΔC(K119-E188) in pMS108 backbone -HygroR
pMS-1704	Myc-Emc10(K118G K119K K120G T121G K122G T123G Y124G) in pMS108 backbone -HygroR
pMS-1705	Myc-Emc10(K118D K119K K120D T121D K122D T123D Y124D) in pMS108 backbone -HygroR

Supplementary Table 5. Yeast strains used in this study

Strain	Genotype	reference
BY4741	*MATa his3Δ1 leu2Δ0 met15Δ0 ura3Δ0*	Euroscarf (Cat#Y00000)
CRLY669	*MATa his3Δ1 leu2Δ0 met15Δ0 ura3Δ0 emc5::Emc5-3xFLAG-HPHNT1*	this study
CRLY638	*MATa his3Δ1 leu2Δ0 met15Δ0 ura3Δ0 spf1::Spf1-2xStrep-HISMX*	this study
CRLY670	*MATa his3Δ1 leu2Δ0 met15Δ0 ura3Δ0 spf1::Spf1-2xStrep-HISMX, emc5::Emc5-3xFLAG-HPHNT1*	this study
YMS7564	*MATa his3Δ1 leu2Δ0 met15Δ0 ura3Δ0, EMC5-GFP::His, SPF1-HA::G418, EMC7::Ura3*	this study
YMS7565	*MATa his3Δ1 leu2Δ0 met15Δ0 ura3Δ0, EMC5-GFP::His, SPF1-HA::G418, EMC10::Ura3*	this study
YMS7656	*MATa his3Δ1 leu2Δ0 met15Δ0 ura3Δ0, EMC5-GFP::His, SPF1-HA::G418, SP-myc-EMC7(WT)::Hygro*	this study
YMS7657	*MATa his3Δ1 leu2Δ0 met15Δ0 ura3Δ0, EMC5-GFP::His, SPF1-HA::G418, SP-myc-EMC7-ΔI160-K216::Hygro*	this study
YMS7658	*MATa his3Δ1 leu2Δ0 met15Δ0 ura3Δ0, EMC5-GFP::His, SPF1-HA::G418, SP-myc-EMC7-T120A/Y121A::Hygro*	this study
YMS7661	*MATa his3Δ1 leu2Δ0 met15Δ0 ura3Δ0, EMC5-GFP::His, SPF1-HA::G418, SP-myc-EMC10(WT)::Hygro*	this study
YMS7662	*MATa his3Δ1 leu2Δ0 met15Δ0 ura3Δ0, EMC5-GFP::His, SPF1-HA::G418, SP-myc-EMC10-ΔK119-E188::Hygro*	this study
YMS7663	*MATa his3Δ1 leu2Δ0 met15Δ0 ura3Δ0, EMC5-GFP::His, SPF1-HA::G418, SP-myc-EMC10-K118G K119K K120G T121G K122G T123G Y124G::Hygro*	this study
YMS7664	*MATa his3Δ1 leu2Δ0 met15Δ0 ura3Δ0, EMC5-GFP::His, SPF1-HA::G418, SP-myc-EMC10-K118D K119K K120D T121D K122D T123D Y124D::Hygro*	this study
YMS6456	*MATa his3Δ1 leu2Δ0 met15Δ0 ura3Δ0 HO::G418*	Schuldiner strain collection
YEF0100	*MATa his3Δ1 leu2Δ0 met15Δ0 ura3Δ0 Δemc3::G418*	this study
YEF0101	*MATa his3Δ1 leu2Δ0 met15Δ0 ura3Δ0 Δspf1::G418*	this study
YEF0159	*MATa his3Δ1 leu2Δ0 met15Δ0 ura3Δ0 HO::G418, NAT::TEFpr-GFP-GEM1*	this study
YEF0160	*MATa his3Δ1 leu2Δ0 met15Δ0 ura3Δ0 Δemc3::G418, NAT::TEFpr-GFP-GEM1*	this study
YEF0161	*MATa his3Δ1 leu2Δ0 met15Δ0 ura3Δ0 Δspf1::G418, NAT::TEFpr-GFP-GEM1*	this study

Supplementary Figures

Supplementary Figure 1

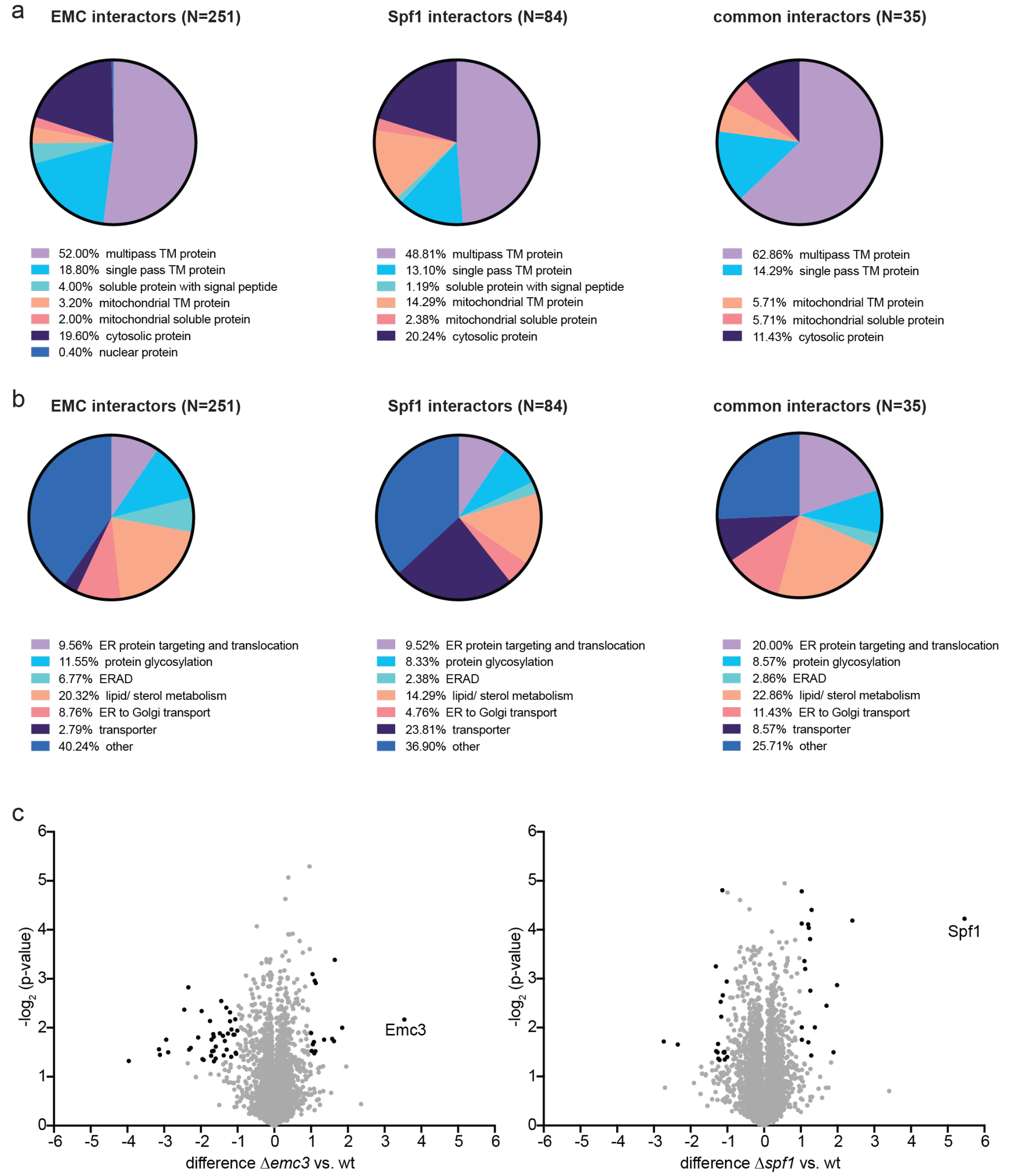

SI Fig. 1. Classification of endogenous EMC and Spf1 interactors as well as common interaction partners

a. Classification of endogenous EMC and Spf1 interactors as well as common interactors identified by MS according to their subcellular location (secretory pathway, mitochondria, cytosol, nucleus), distinguishing between soluble and transmembrane proteins.
b. Classification of endogenous EMC and Spf1 interactors as well as common interactors identified by MS according to their functional annotation.
c. The overall proteome of Δemc3 (left) and Δspf1 (right) cells displays only modest changes compared to wt cells. Three samples for each condition were measured and an unpaired two-tailed Student's t-test was applied. Significant hits (>1 log_2-fold change; p-value <0.05) are labeled in black.

Supplementary Figure 2

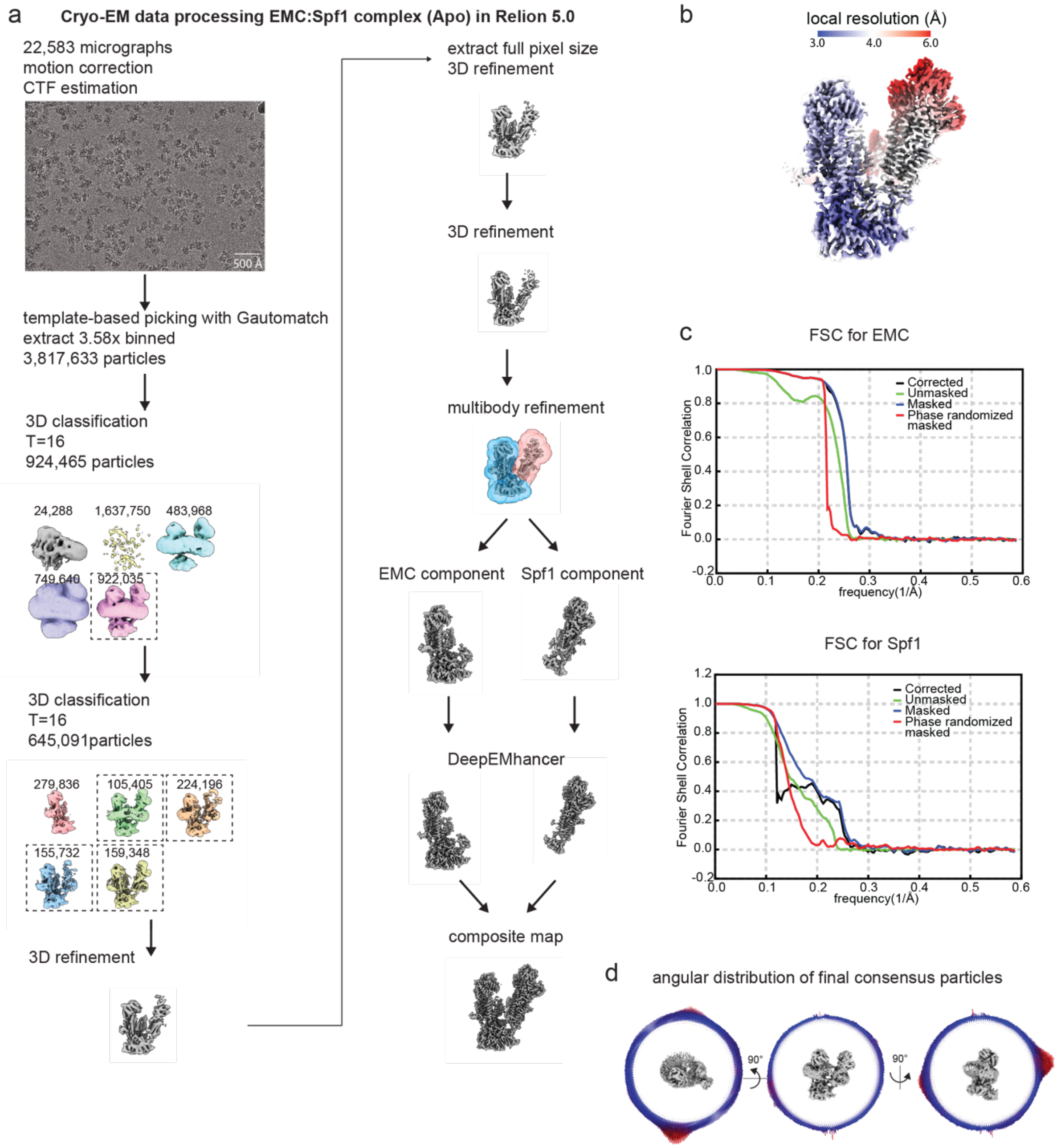

SI Fig. 2. Cryo-EM data processing of the recombinant EMC:Spf1^{apo} complex

a. Processing scheme for the EMC:Spf1^{apo} complex in Relion. Scalebar on the micrograph corresponds to 500 Å.
b. Local resolution estimation projected onto the composite map.
c. The final 3D reconstructions yielded resolutions of 3.8 Å for the EMC component and 4.2 Å for the Spf1 component by the Fourier shell correlation of 0.143, respectively. Gold-standard Fourier shell correlation plots are shown for each component.
d. Angular distribution plot of the particles for the final consensus map before multibody refinement was performed.

Supplementary Figure 3

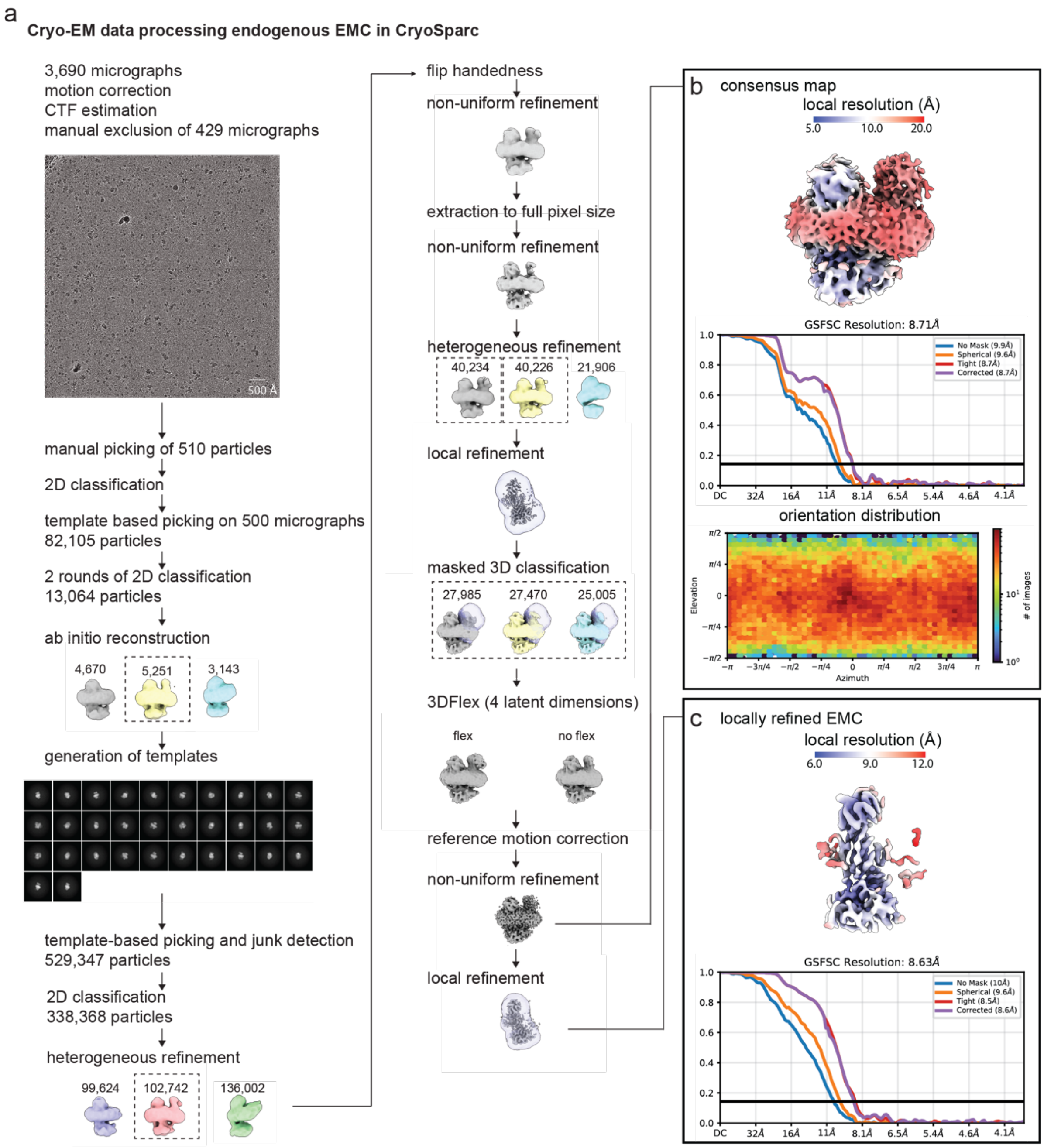

SI Fig. 3. Cryo-EM data processing of the endogenous EMC from *S. cerevisiae* in complex with Spf1

a. Processing scheme for the endogenous EMC from yeast in Cryosparc. Scalebar on the micrograph corresponds to 500 Å.
b. Local resolution estimation projected onto the consensus map. The final 3D reconstruction yielded a resolution of 8.71 Å by the Fourier shell correlation of 0.143. The gold-standard Fourier shell correlation plot as well as the orientation distribution plot of the particles for the final reconstruction is shown.
c. Local resolution estimation projected onto the map of the locally refined EMC. The final 3D reconstruction yielded a resolution of 8.63 Å by the Fourier shell correlation of 0.143. The gold-standard Fourier shell correlation plot is shown.

Supplementary Figure 4

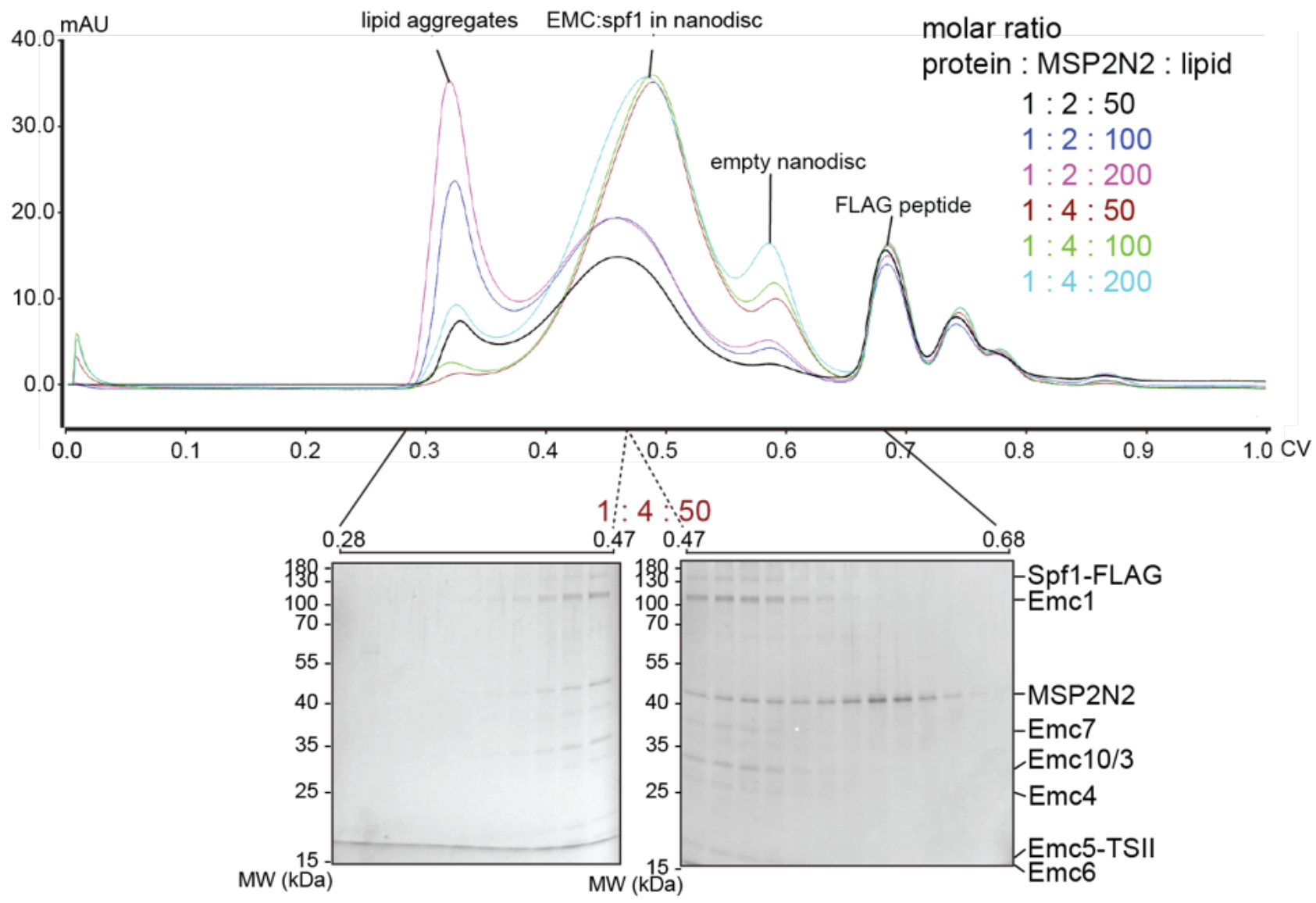

SI Fig. 4. Reconstitution of recombinant EMC:Spf1 into MSP2N2 lipid nanodisc
Different ratios for EMC:Spf1, nanodisc scaffold (MSP2N2) and lipid were tested to identify conditions for the highest reconstitution yield. The EMC:Spf1 concentration was kept constant at 1 µM. SEC showed best yields with lowest amounts of excess lipids or empty nanodisc for the 1:4:50 molar ratio (brown trace). Indicated fractions of this sample were analyzed by SDS-PAGE for protein content to confirm the co-elution.

Supplementary Figure 5

Example fits of model to cryo-EM map over key interactions

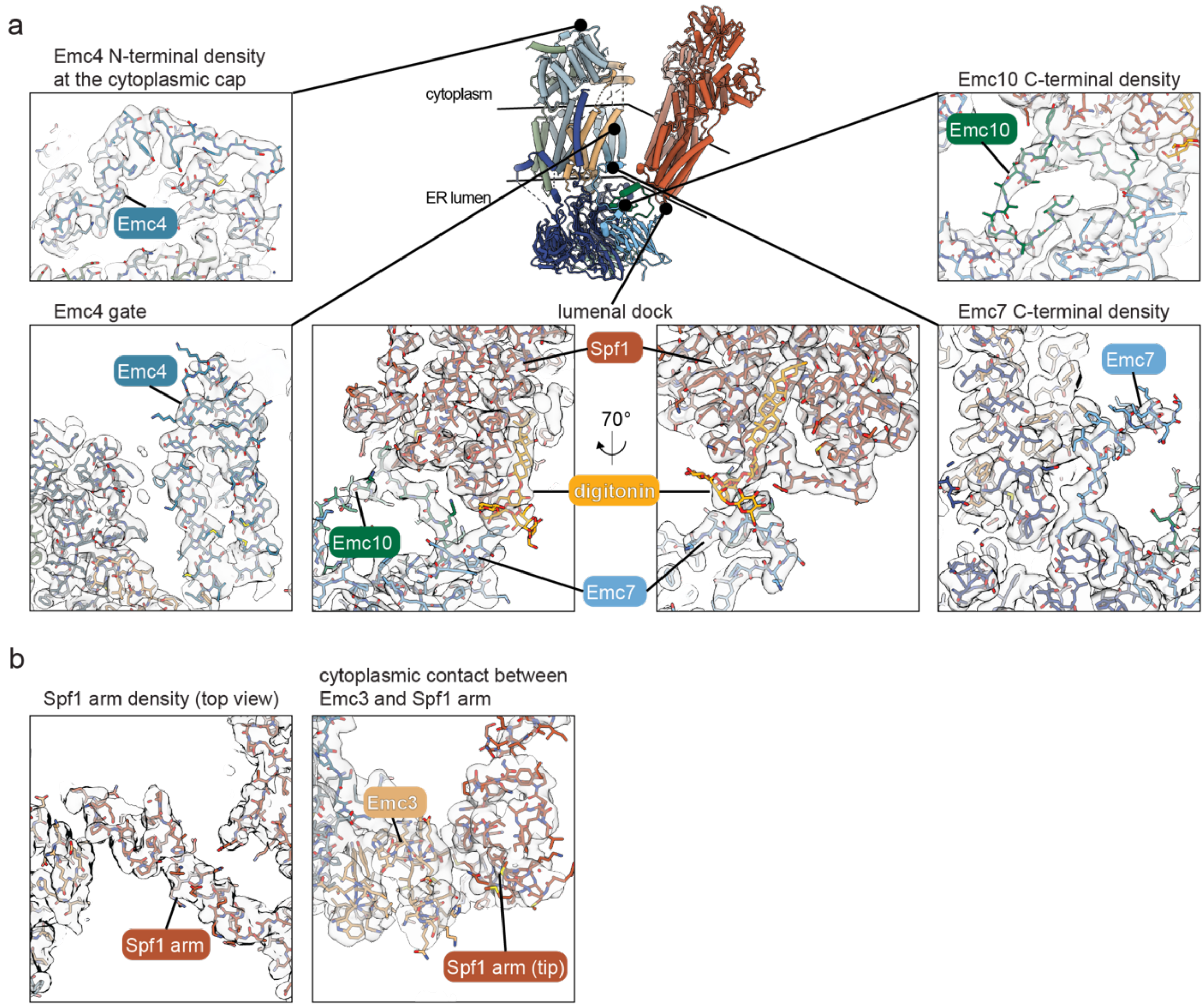

SI Fig. 5. Fit of the model to the cryo-EM maps

a. The model of the EMC:Spf1^{apo} complex is depicted in the center and where indicated, details are shown for regions of relevance for this study and for regions which have been resolved better than in previous studies. These include the N-terminus of Emc4, the gate helices of Emc4, the lumenal dock between Emc7, Emc10 and Spf1, as well as residues close to the C-termini of Emc7 and Emc10.
b. Model-to-map fit for the arm of Spf1, contacting Emc3 in the cytoplasm in the EMC:Spf1A$^{TP\gamma S}$ map.

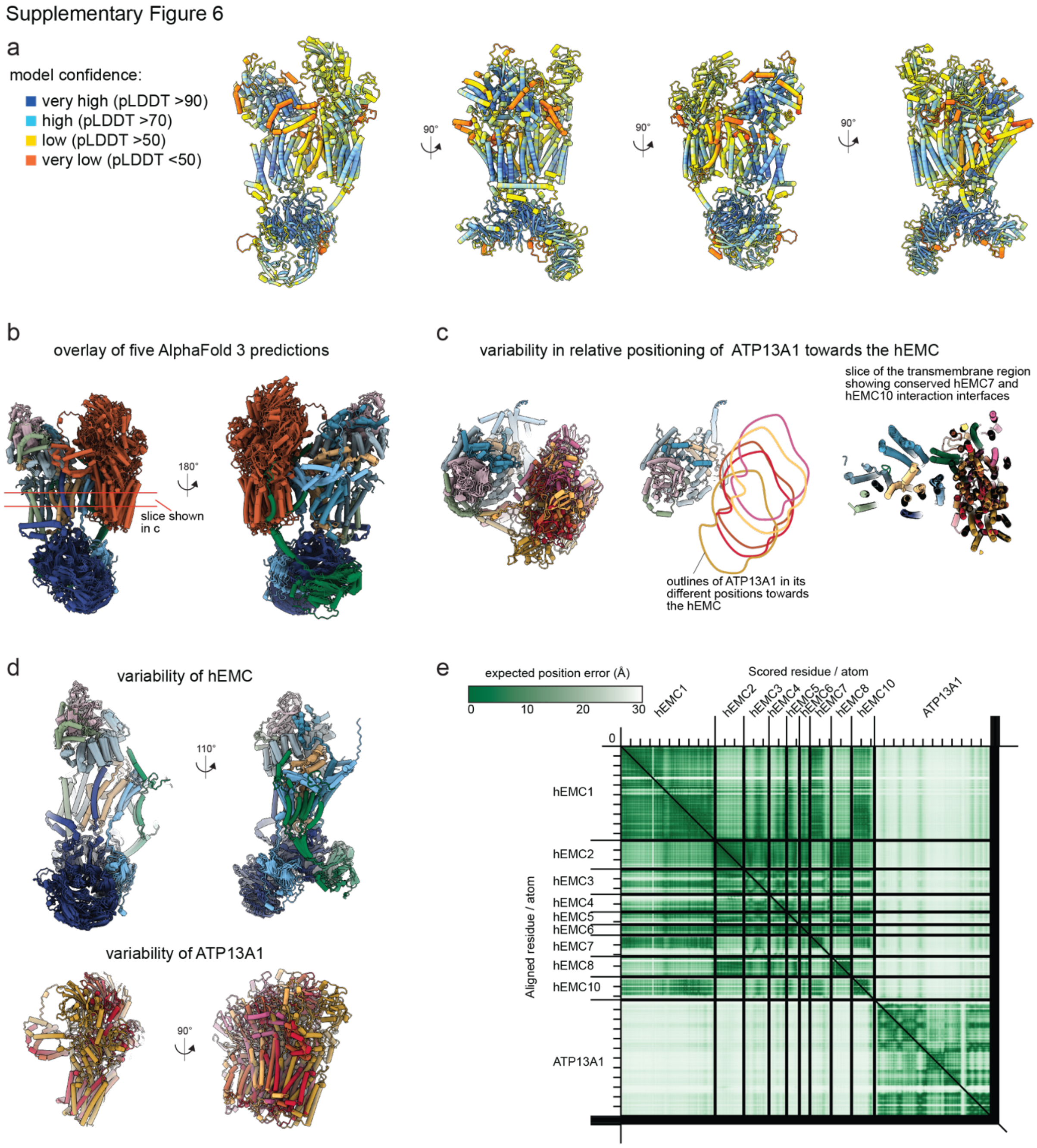

SI Fig. 6. AlphaFold 3 prediction of the hEMC:ATP13A1 complex

a. The predicted local-distance difference test (pLDDT) values were plotted on a representative model from the five predicted structural models as a confidence measure for the per-residue accuracy.
b. Five predicted models of the hEMC:ATP13A1 complex. The models were aligned on the hEMC.
c. Top view of the aligned five predicted models of the hEMC:ATP13A1 complex shows variability between the models, particularly with respect to the positioning of ATP13A1 relative to the hEMC. The models were aligned on the hEMC, which remains constant throughout the predictions, hence only one model of the EMC is shown for clarity. ATP13A1 was colored in shades of yellow, red, orange and pick to distinguish between different predictions.

d. Details on the variability between the AlphaFold models. The same alignment as in c is shown, for better visibility the EMC (top) and ATP13A1 (bottom) are displayed separately.
e. The predicted alignment error (PAE) plot for one of the five predicted structural models as a measure for the reliability of relative positioning and orientation of different domains.

Supplementary Figure 7

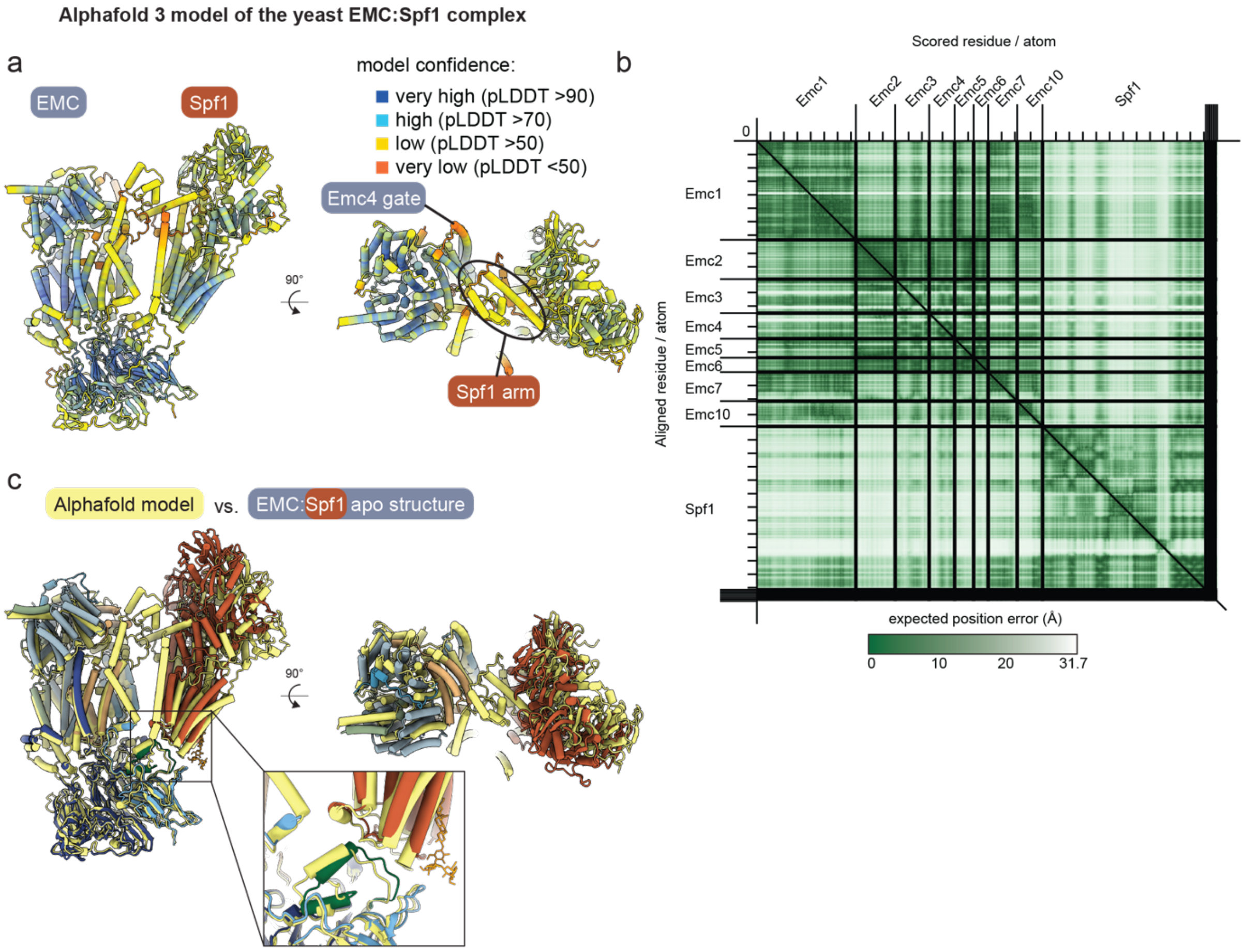

SI Fig. 7. AlphaFold 3 prediction of the yeast EMC:Spf1 complex

a. One of the five predicted models for the EMC:Spf1 complex colored according to the predicted local-distance difference test (pLDDT) values as a confidence measure for the per-residue accuracy. Please note that the model closest to our experimental structure was selected for display, yet the observed overall variability between the predictions was low.
b. The predicted alignment error (PAE) plot for one of the five predicted structural models as a measure for the reliability of relative positioning and orientation of different domains.
c. Alignment of the AlphaFold-generated structural model (yellow) and our experimentally derived structural model of the EMC:Spf1^{apo} complex. The relative positioning of both partners is overall predicted correctly. The lumenal dock is predicted very accurately.

Supplementary Figure 8

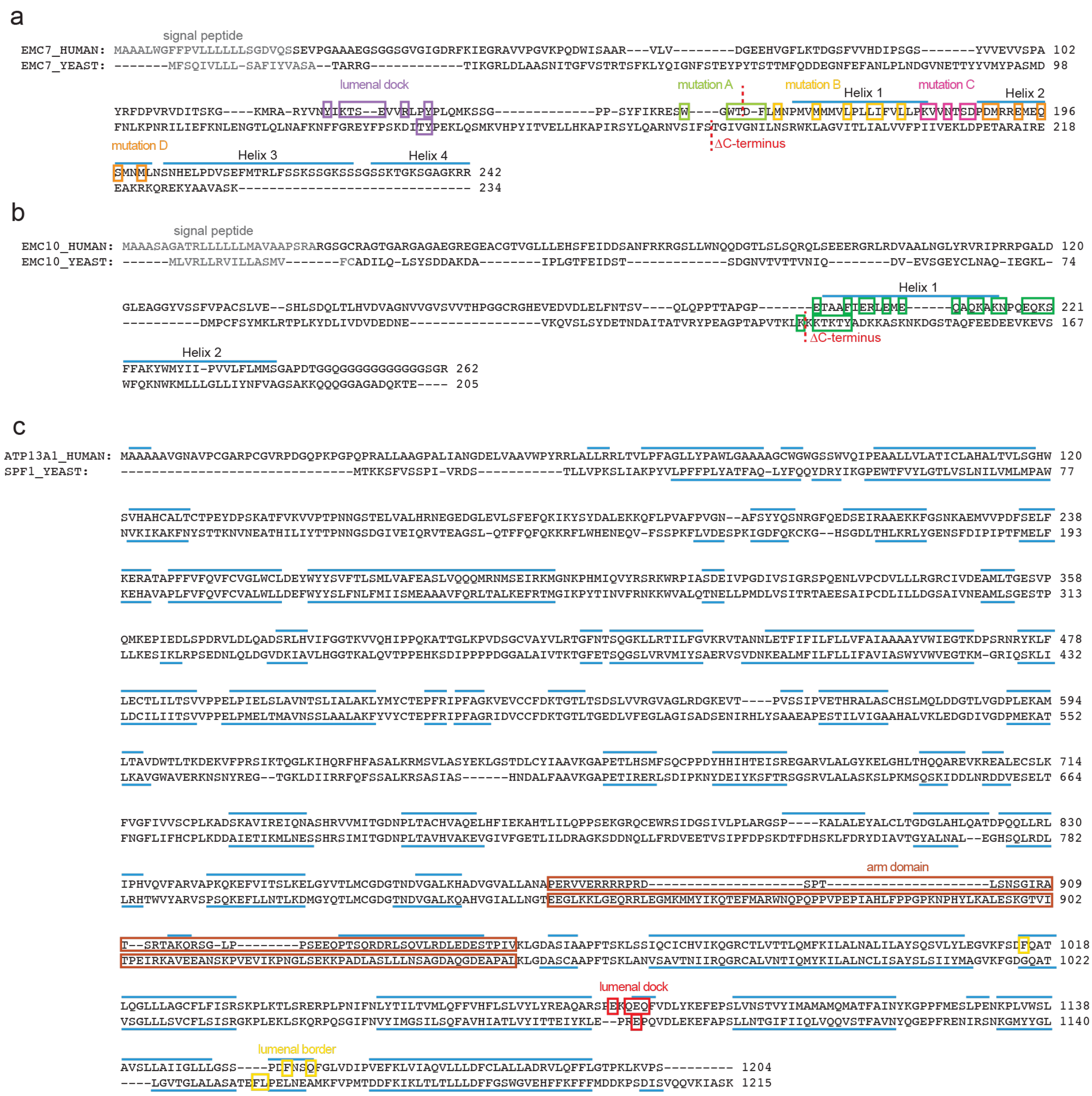

SI Fig. 8. Sequence alignments of the human and yeast orthologs for Emc7, Emc10 and ATP13A1/ Spf1

a. Sequence alignment of hEMC7/Emc7 from *H. sapiens* and *S. cerevisiae*, respectively. Features highlighted: signal sequence (grey), α-helices in the human sequence according to the AlphaFold model (blue). Mutations included in this manuscript are color coded: yeast Emc7 loop at the lumenal dock (T120A Y121A) in purple and the corresponding loop mutation in hEMC. hEMC7 "mutation A" at the lumenal membrane border in green, "mutation B" in helix 1 in yellow,"mutation C" in the linker between helix 1 and 2 in pink, "mutation D" in helix 2 in orange. Sites for C-terminal truncation are marked with a dashed red line.

b. Sequence alignment of hEMC10/Emc10 from *H. sapiens* and *S. cerevisiae*, respectively. Features highlighted: signal sequence (grey), α-helices in the human sequence according to the AlphaFold model (blue). Mutations included in this manuscript are highlighted in green and the site for C-terminal truncation of the yeast subunit is marked with a dashed red line.

c. Sequence alignment of ATP13A1 from *H. sapiens* and Spf1 from *S. cerevisiae.* α-helical segments are labeled with blue bars as modeled in our apo structure of the Emc:Spf1 complex or as predicted in the AlphaFold model (ATP13A1). The arm domain is highlighted in copper. Mutations included in this manuscript are color coded: mutations at the lumenal dock in yeast and the corresponding sequence in human in red, mutations at the border between membrane region and ER lumen, which showed to be highly effective, in yellow.

Supplementary Figure 9

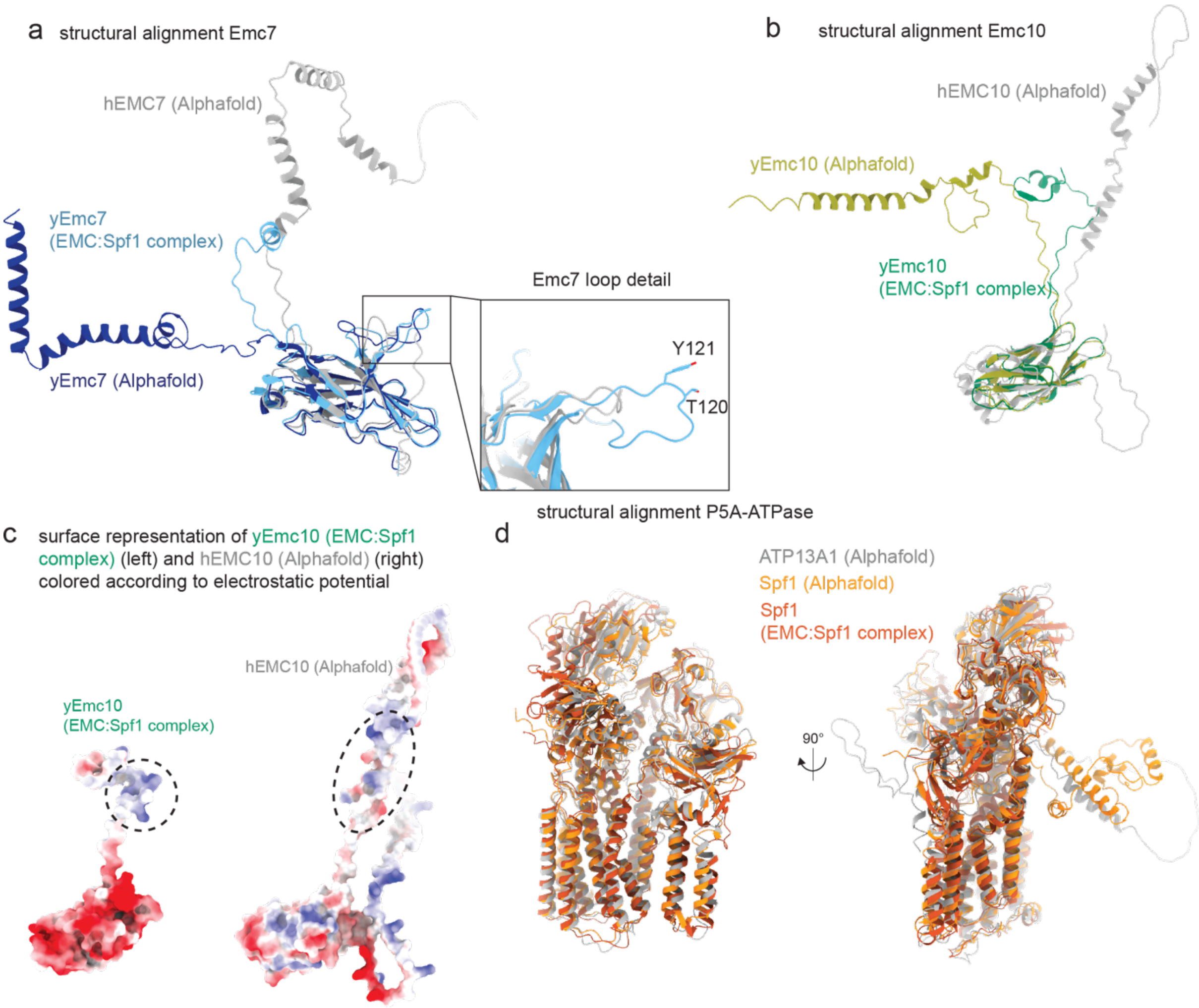

SI Fig. 9. Structural comparison of the human and yeast models for the proteins Emc7, Emc10 and ATP13A1/ Spf1

a. Structural alignment of yeast and human Emc7/hEMC7, aligned on the β-sandwich domain. The subunit as modeled in the EMC:Spf1 complex is colored in light blue, the AlphaFold model for the full sequence of the yeast subunit in dark blue and for the human subunit in grey. The detail shows the Emc7 loop involved in the lumenal dock interface in the EMC:Spf1 complex and the two residues mutated in this study. This loop region is shortened and predicted to be in a different conformation in the hEMC7.
b. Structural alignment of yeast and human Emc10/hEMC10, aligned on the β-sandwich domain. The subunit as modeled in the EMC:Spf1 complex is colored in dark green, the AlphaFold model for the full sequence of the yeast subunit in light green and for the human subunit in grey.
c. Surface representation of yeast Emc10 as modeled in the EMC:Spf1 complex and of the hEMC10 AlphaFold model colored according to electrostatic potential. The patch of positively charged residues that was mutated in Fig. 3c is indicated with a dashed line.
d. Structural alignment of yeast Spf1 and human ATP13A1. Spf1 as modeled in the EMC:Spf1 complex is colored in orange, the AlphaFold model for the full sequence of the yeast subunit in yellow and for the human ATP13A1 in grey.

Supplementary Figure 10

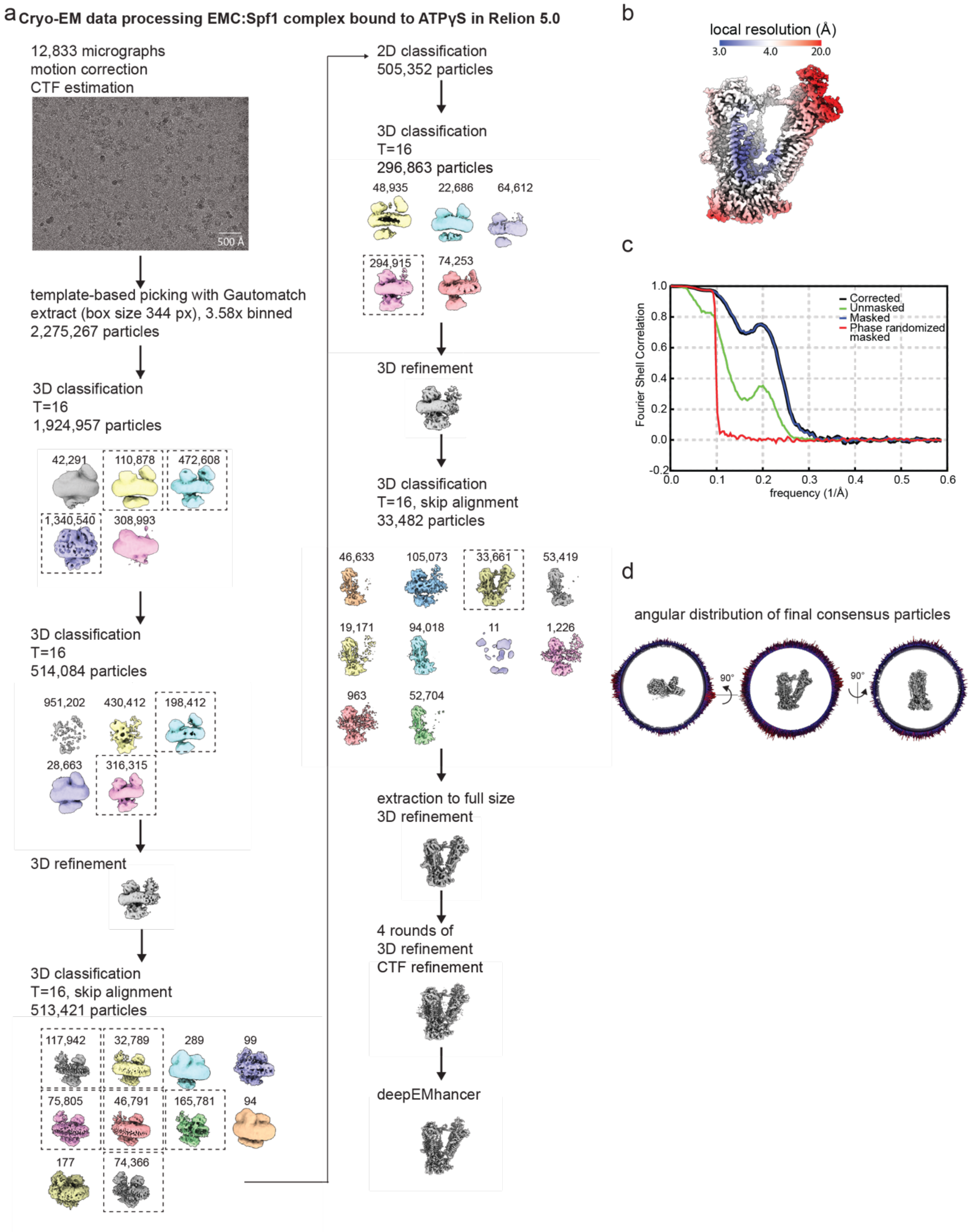

SI Fig. 10. Cryo-EM data processing of the EMC:Spf1$^{ATP\gamma S}$ complex

a. Processing scheme for the EMC:Spf1$^{ATP\gamma S}$ complex in Relion. Scalebar on the micrograph corresponds to 500 Å.
b. Local resolution estimation projected onto the deepEMhancer sharpened map.
c. The final 3D reconstruction yielded a resolution of 3.8 Å by the Fourier shell correlation of 0.143 as shown on the gold-standard Fourier shell correlation plot.
d. Angular distribution plot of the particles for the final reconstruction.

Supplementary Figure 11

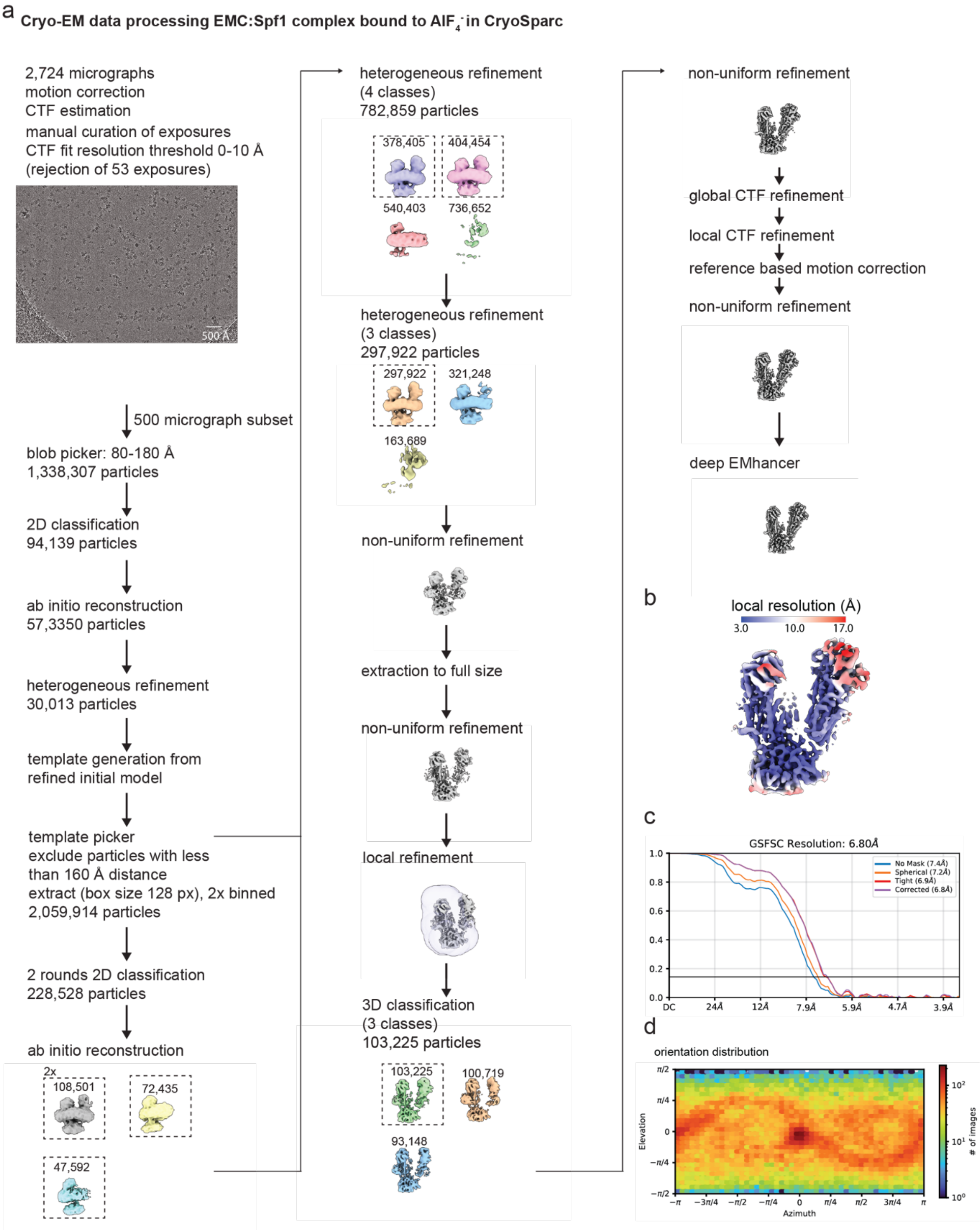

SI Fig. 11. Cryo-EM data processing of the EMC:Spf1^{AlF4-} complex

a. Processing scheme for the EMC:Spf1^{AlF4-} complex in CryoSparc. Scalebar on the micrograph corresponds to 500 Å.
b. Local resolution estimation projected onto the deepEMhancer sharpened map.
c. The final 3D reconstruction yielded a resolution of 6.8 Å by the Fourier shell correlation of 0.143 as shown on the gold-standard Fourier shell correlation plot.
d. Angular distribution plot of the particles for the final reconstruction.

Supplementary Figure 12

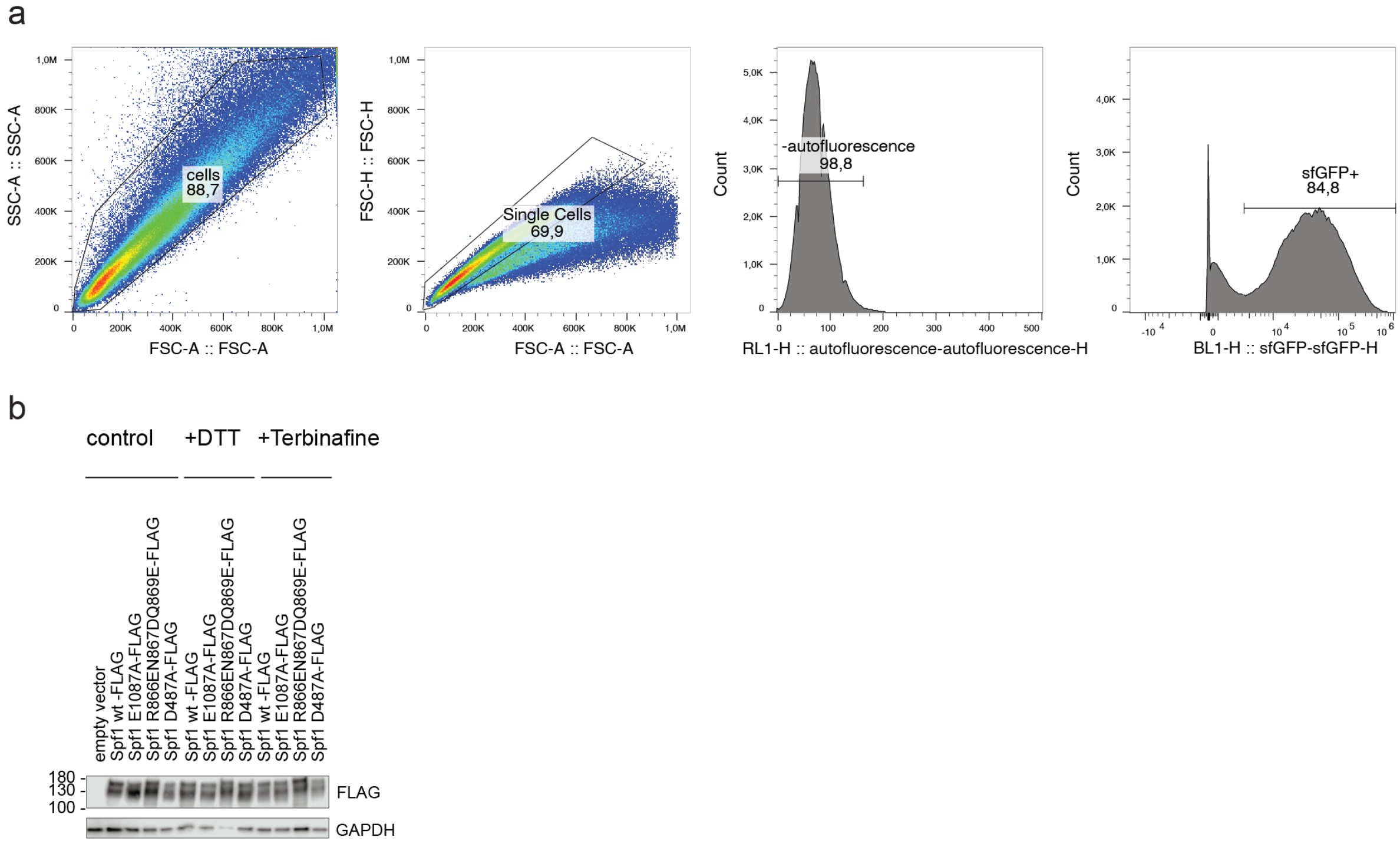

SI Fig. 12. Representative dot plots and histograms to exemplify the gating strategy applied to all flow cytometry data

a. From left to right: yeast cells were selected based on their behavior in forward (FSC) and side (SSC) scatter. Duplicates were excluded from singlets comparing area and signal height for the forward scatter. Next, cells that show high autofluorescent properties when illuminated with a 637 nm laser were excluded. The population expressing the fluorescent protein timer construct was selected based on the sfGFP signal.

b. Expression levels of Spf1-FLAG rescue constructs expressed in the *Δspf1* strain under control and stress conditions as assessed by western blot. One representative replicate of three replicates is shown.

Uncropped scans of gels and western blots from the SI

Supplementary Figure 4

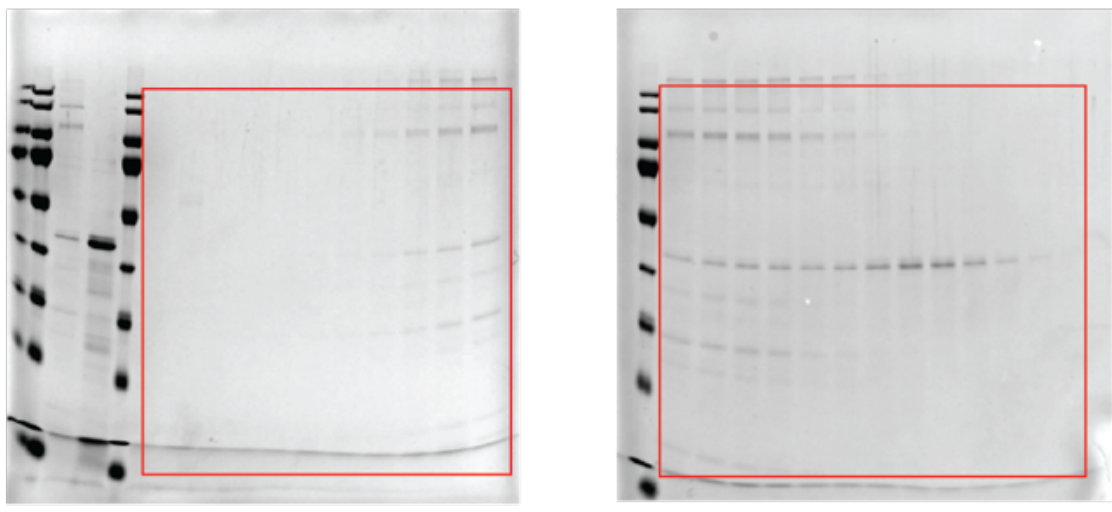

Supplementary Figure 12b

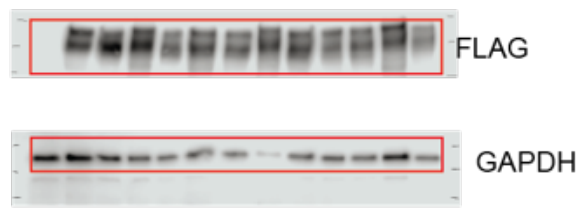

Supplement: Supplementary file 1 [file 41594_2026_1883_MOESM1_ESM.pdf]
