# Supplementary material for: Structural basis of an endoplasmic reticulum EMC:Spf1 insertase–dislocase complex
Source: Nat Struct Mol Biol. 2026 Oct 13;33(10):1548–60. doi: 10.1038/s41594-026-01883-8 (PMC13672079; doi:10.1038/s41594-026-01883-8)
Supplement: Supplementary file 2 [file 41594_2026_1883_MOESM2_ESM.pdf]

nature portfolio

Corresponding author(s): Matthias J Feige, Brenda A Schulman, Carolin J Klose

Last updated by author(s): Oct 6, 2026

Reporting Summary

Nature Portfolio wishes to improve the reproducibility of the work that we publish. This form provides structure for consistency and transparency in reporting. For further information on Nature Portfolio policies, see our Editorial Policies and the Editorial Policy Checklist.

Statistics

For all statistical analyses, confirm that the following items are present in the figure legend, table legend, main text, or Methods section.

n/a	Confirmed	
☐	☒	The exact sample size (*n*) for each experimental group/condition, given as a discrete number and unit of measurement
☐	☒	A statement on whether measurements were taken from distinct samples or whether the same sample was measured repeatedly
☐	☒	The statistical test(s) used AND whether they are one- or two-sided<br>*Only common tests should be described solely by name; describe more complex techniques in the Methods section.*
☒	☐	A description of all covariates tested
☐	☒	A description of any assumptions or corrections, such as tests of normality and adjustment for multiple comparisons
☐	☒	A full description of the statistical parameters including central tendency (e.g. means) or other basic estimates (e.g. regression coefficient) AND variation (e.g. standard deviation) or associated estimates of uncertainty (e.g. confidence intervals)
☐	☒	For null hypothesis testing, the test statistic (e.g. *F*, *t*, *r*) with confidence intervals, effect sizes, degrees of freedom and *P* value noted<br>*Give P values as exact values whenever suitable.*
☒	☐	For Bayesian analysis, information on the choice of priors and Markov chain Monte Carlo settings
☒	☐	For hierarchical and complex designs, identification of the appropriate level for tests and full reporting of outcomes
☒	☐	Estimates of effect sizes (e.g. Cohen's *d*, Pearson's *r*), indicating how they were calculated

Our web collection on statistics for biologists contains articles on many of the points above.

Software and code

Policy information about availability of computer code

Data collection	mass spectrometry: otofControl 6.0, cryo-EM data: SerialEM v.3.8 and v.4.1, gels and western blots: Amersham Imager 600 and Vilber Lourmat Fusion FX Edge, microscopy: LasX, flow cytometry: Attune NxT Cytometric Software
Data analysis	Cryo-EM data processing: CryoSparc v4.7.1, RELION v5.0 beta, Gautomatch v0.56, CTFFIND v4.1; Structure Analysis and Visualization: ChimeraX v1.9, DeepEMhancer version 2020.09.07 (https://github.com/rsanchezgarc/deepEMhancer); Model Building & Refinement: COOT v0.9.6, Phenix.refine v1.21.1, AlphaFold2, AlphaFold3; Sequence alignment: ClustalOmega; Biochemical Data Analysis: ImageJ Fiji 2.14.0/1.54f, GraphPad Prism 10; Mass spectrometry data analysis: MaxQuant v2.0.1.0, Perseus 1.6.15.0; Microscopy data analysis: LasX, ImageJ Fiji 2.14.0/1.54f; Flow cytometry data analysis: FlowJo v. 10; Figure Generation: Adobe Illustrator 2025<br>All software used was either available commercially (CryoSPARC, GraphPad Prism, FlowJo, Adobe Illustrator) or as open source (others). No custom code was generated or used for analyses in this study.

For manuscripts utilizing custom algorithms or software that are central to the research but not yet described in published literature, software must be made available to editors and reviewers. We strongly encourage code deposition in a community repository (e.g. GitHub). See the Nature Portfolio guidelines for submitting code & software for further information.

Data

Policy information about availability of data

All manuscripts must include a data availability statement. This statement should provide the following information, where applicable:

- Accession codes, unique identifiers, or web links for publicly available datasets
- A description of any restrictions on data availability
- For clinical datasets or third party data, please ensure that the statement adheres to our policy

Structural data will be available from EMDB and RCSB upon publication under the following identifiers:
- composite map of the EMC:Spf1 complex EMD-59745 and PDB 34BQ (consensus map: EMD-59738, Spf1 component: EMD-59736, EMC component: EMD-59737);
- EMC:Spf1ATP�S EMD-59746 PDB: 34BR
- endogenous EMC:Spf1 EMD-54652 (locally refined endogenous EMC: EMD-54648)
- EMC:Spf1AlF4- EMD-54635.

Proteomics data have been deposited at the ProteomeXchange repository under the project accession number PXD067089.

Research involving human participants, their data, or biological material

Policy information about studies with human participants or human data. See also policy information about sex, gender (identity/presentation), and sexual orientation and race, ethnicity and racism.

Reporting on sex and gender	N/A
Reporting on race, ethnicity, or other socially relevant groupings	N/A
Population characteristics	N/A
Recruitment	N/A
Ethics oversight	N/A

Note that full information on the approval of the study protocol must also be provided in the manuscript.

Field-specific reporting

Please select the one below that is the best fit for your research. If you are not sure, read the appropriate sections before making your selection.

☒ Life sciences ☐ Behavioural & social sciences ☐ Ecological, evolutionary & environmental sciences

For a reference copy of the document with all sections, see nature.com/documents/nr-reporting-summary-flat.pdf

Life sciences study design

All studies must disclose on these points even when the disclosure is negative.

Sample size	Sample size calculations were not performed. Selected sample sizes were designed to ensure clear and reliable interpretation of the results. Biochemical experiments were performed in true replicates (different passages of cells/ different clones, different days) to ensure reproducibility. All biochemical experiments were repeated at least three times to allow for statistical evaluation.
Data exclusions	No data were excluded.
Replication	Reproducibility and reliability of the data has been ensured in multiple ways. All biochemical experiments have been performed at least in independent triplicates on different occasions yielding the same result.
Randomization	Sample randomization is not applicable to our biochemical and cell biological assays and hence was not performed.
Blinding	No blinding was performed.

Reporting for specific materials, systems and methods

We require information from authors about some types of materials, experimental systems and methods used in many studies. Here, indicate whether each material, system or method listed is relevant to your study. If you are not sure if a list item applies to your research, read the appropriate section before selecting a response.

Materials & experimental systems

n/a	Involved in the study
☐	☒ Antibodies
☐	☒ Eukaryotic cell lines
☒	☐ Palaeontology and archaeology
☒	☐ Animals and other organisms
☒	☐ Clinical data
☒	☐ Dual use research of concern
☒	☐ Plants

Methods

n/a	Involved in the study
☒	☐ ChIP-seq
☐	☒ Flow cytometry
☒	☐ MRI-based neuroimaging

Antibodies

Antibodies used

ATP13A1 (Proteintech, 16244-1-AP, 1:1,000), EMC1 (Novus Biologicals, NBP2-59097 and NBP3-18427, 1:500), EMC4 (Abcam, ab184544, 1:2,000), EMC7 (Proteintech, 27550-1-AP, 1:1,000), HA.11 (Biolegend, Poly9023, 1:1,000), c-myc (Proteintech, 16286-1-AP, 1:1,000), FLAG (Proteintech, 20543-1-AP, 1:1,000), NWSHPQFEK 5A9F9 (Genscript, A01732, 1:1,000), GFP (Proteintech, 50430-2-AP, 1:1,000), Hsc70 (Santa Cruz Biotechnology, sc-7298, 1:1,000), GAPDH-HRP (Proteintech, HRP-60004, 1:5,000), anti-rabbit IgG-HRP (Santa Cruz Biotechnology, sc-2357, 1:5,000), m-IgGk BP-HRP, sc-516102, 1:5,000)

Validation

ATP13A1: validation by knockout, knockdown and overexpression experiments (not shown), widely used in the field e.g. McKenna et al. 2020
EMC4: validation by siRNA knockdown (not shown), widely used in the field e.g. Miller-Vedam et al. 2020, Tian et al. 2019, Coelho et al. 2019
EMC1: validation by siRNA knockdown and overexpression of the target protein and analysis on a western blot in Klose et al. 2025
HA.11: validation on supplier site, in different other studies and by overexpression of HA-tagged proteins vs. untagged
c-myc: validation on supplier site, in different other studies and by overexpression of myc-tagged proteins vs. untagged
FLAG: validation on supplier site, in different other studies and by overexpression of FLAG-tagged proteins vs. untagged
NWSHPQFEK 5A9F9: validation on supplier site, in different other studies and by overexpression of twinStrep-tagged proteins vs. untagged
GFP: validation on supplier site
Hsc70: validation on supplier site
GAPDH-HRP: validation on supplier site

Eukaryotic cell lines

Policy information about cell lines and Sex and Gender in Research

Cell line source(s)

293T cells (Sigma-Aldrich, 12022001, human, female)
BY4741 (Euroscarf cat#Y00000)
Sf9 insect cells (Thermo Fisher B85502)
Hi5 insect cells (BTI-TN-5B1-4; Thermo Fisher 11496016)

Authentication

Cell lines were obtained from indicated sources and not further authenticated

Mycoplasma contamination

All cells were regularly tested negative for mycoplasma contamination

Commonly misidentified lines
(See ICLAC register)

No commonly misidentified cell lines were used in this study.

Plants

Seed stocks

Report on the source of all seed stocks or other plant material used. If applicable, state the seed stock centre and catalogue number. If plant specimens were collected from the field, describe the collection location, date and sampling procedures.

Novel plant genotypes

Describe the methods by which all novel plant genotypes were produced. This includes those generated by transgenic approaches, gene editing, chemical/radiation-based mutagenesis and hybridization. For transgenic lines, describe the transformation method, the number of independent lines analyzed and the generation upon which experiments were performed. For gene-edited lines, describe the editor used, the endogenous sequence targeted for editing, the targeting guide RNA sequence (if applicable) and how the editor was applied.

Authentication

Describe any authentication procedures for each seed stock used or novel genotype generated. Describe any experiments used to assess the effect of a mutation and, where applicable, how potential secondary effects (e.g. second site T-DNA insertions, mosiacism, off-target gene editing) were examined.

Flow Cytometry

Plots

Confirm that:

☒ The axis labels state the marker and fluorochrome used (e.g. CD4-FITC).

☒ The axis scales are clearly visible. Include numbers along axes only for bottom left plot of group (a 'group' is an analysis of identical markers).

☒ All plots are contour plots with outliers or pseudocolor plots.

☒ A numerical value for number of cells or percentage (with statistics) is provided.

Methodology

Sample preparation	Yeast strains were co-transformed with one plasmid containing an Spf1 variant or empty vector and one plasmid encoding for the protein of interest fused to the mCherry-EGFP timer as described previously. Transformants were plated on a plate containing the respective selection markers, positive clones were isolated and grown in selection medium overnight. The cultures were diluted to an OD600 of 0.2 in SDC medium and grown until mid-log phase before analysis on an AttuneNxt Flow Cytometer (Thermo Fisher). To induce the unfolded protein response, cells were treated with 2 mM DTT for 3 h after reaching mid-log phase. For terbinafine treatment, overnight cultures were directly diluted into SDC medium containing 5 µg/ml Terbinafine hydrochloride.
Instrument	Attune NxT Flow Cytometer (Thermo Fisher)
Software	Data was acquired using the Attune NxT Cytometric Software (Thermo Fisher) and analyzed in FlowJo v.10 (BD)
Cell population abundance	Per replicate, at least 50,000 cells in the final gate were analyzed. After selection for single cells, a small fraction (<1%) was excluded due to high autofluorescence and of this population usually 80-100 % were positive for sfGFP and hence subject to further analysis.
Gating strategy	The gating strategy is visualized in SI Fig. 12a: Single cells were selected according to behavior in forward and sideward scatter, excluding duplicate and autofluorescent events and gating for sfGFP-positive cells. The mCherry:sfGFP ratio for the final population was derived from the respective signal heights.

☒ Tick this box to confirm that a figure exemplifying the gating strategy is provided in the Supplementary Information.
