# Supplementary material for: Structural basis of an endoplasmic reticulum EMC:Spf1 insertase–dislocase complex
Source: Nat Struct Mol Biol. 2026 Oct 13;33(10):1548–60. doi: 10.1038/s41594-026-01883-8 (PMC13672079; doi:10.1038/s41594-026-01883-8)
Supplement: Supplementary file 3 [file 41594_2026_1883_MOESM3_ESM.pdf]

nature portfolio

Peer Review File

Structural basis of an endoplasmic reticulum EMC:Spf1 insertase-dislocase complex

Corresponding Author: Professor Matthias Feige

A version of this paper was originally rejected for publication by Nature Structural & Molecular Biology, however that decision was reconsidered after appeal by the authors.

Version 0:

Decision Letter:

11th Aug 2025

Dear Dr. Feige,

Thank you again for submitting your manuscript "Structural basis of an EMC:Spf1 insertase-dislocase complex in the eukaryotic endoplasmic reticulum". I apologize for the delay in sharing our decision. I'm writing to let you know that we have decided to send your manuscript for peer review, but several points require your attention before we can proceed with peer review.

I am re-opening the manuscript submission system for you to resubmit your manuscript with all associated files needed for peer review directly, within 2-3 business days if possible. Please follow the link at the bottom of this email to upload the documents listed below. If you have any issues, please reach out to us before completing the submission.

1- We require official *final*(wwPDB validation reports for newly described atomic structures, as per journal policy. We also request that authors provide cryo-EM maps, half-maps and models, as well as maps and models obtained from subtomogram averaging if it applies to the work, to help the reviewers in assessing the work. We recommend the use of figshare in our system, which allows for provision of anonymous access links for the referees (https://www.springernature.com/gp/authors/research-data/figshare-integration). Alternatively, please upload .zip folders directly with the submission. To ensure the ease of reviewer access to the data, please specify in the Data Availability section where the files can be found (i.e., provide a figshare link or direct the reader to the manuscript files).

2- We want to ensure that the methods and statistics reporting in our papers are of the highest quality. To that end, we ask authors to fill out a Reporting Summary that collects information on experimental design and reagents. If your paper includes ChIP-seq, flow cytometry or MRI data, we ask you take special care to complete those sections of the Reporting Summary as this data will aid greatly in the review of your manuscript. This document can be found by following the link below:

Reporting Summary:
https://www.nature.com/documents/nr-reporting-summary.pdf

3- In order for us to proceed with peer review, please provide accession numbers and reviewer tokens to access sequencing or proteomics datasets if any unpublished datasets are part of your study. Please add this information to your manuscript file.

4- Lastly, I would like to kindly request that you provide the code used to analyse the data to the reviewers, if newly developed (unpublished) code was used in the work. For the reviewers to evaluate the work adequately, they must be able to test the software/review the code themselves. If you have not yet provided the software, we therefore request that you provide a single compressed zip file containing the software with a readme.txt file or other user manual containing complete instructions for installing and running the software. If appropriate, please provide example data and expected output. Sufficient material should be provided for referees to directly test the performance of the software/algorithm. If the software and materials are small enough to fit in a single compressed zip file under 6MB in size, you may email this file directly to me. If the zip file is between 6 MB and 200 MB, you may upload it to our file transfer site. If necessary, a second zip file up to 200 MB in size can be used to supply the example data. Please let me know if you need to use this option and I’ll send you

further details. Alternatively, you can upload the code to GitHub and provide us with the link.

Please fill out and return to me the code and software submission checklist that will be made available to editors and reviewers during manuscript assessment. Please note that this form is a dynamic 'smart pdf' and must therefore be downloaded and completed in Adobe Reader, instead of opening it in a web browser.
https://www.nature.com/documents/nr-software-policy.pdf

Please use the link below to submit the files. **Please also remember to move forward all other files associated with this version of the paper.**

Link Redacted

Note: This URL links to your confidential home page and associated information about manuscripts you may have submitted, or that you are reviewing for us. If you wish to forward this email to co-authors, please delete the link to your homepage.

We look forward to seeing the revised manuscript and thank you for the opportunity to review your work.

Sincerely,
Kat

Katarzyna Ciazynska, PhD
(she/her)
Senior Editor
Nature Structural & Molecular Biology
https://orcid.org/0000-0002-9899-2428

Version 1:

Decision Letter:

5th Sep 2025

Dear Dr. Feige,

Thank you again for submitting your manuscript "Structural basis of an EMC:Spf1 insertase-dislocase complex in the eukaryotic endoplasmic reticulum". I apologize for the delay in responding, which resulted from the difficulty in obtaining suitable referee reports. Nevertheless, we now have comments (below) from the 3 reviewers who evaluated your paper. In light of those reports, we remain interested in your study and would like to see your response to the comments of the referees, in the form of a revised manuscript.

You will see that while reviewers appreciate the results, they raise several concerns which will need to be addressed in a revision. Specifically, while reviewers #1 and #3 are largely positive, reviewer #2 remarks on the unclear functional relevance of the proposed mechanism. You will see they propose specific experiments to probe this aspect further, and strengthen the manuscript. While we editorially agree that this point will need to be experimentally addressed for further consideration at NSMB, we will understand if you want to explore other experimental approaches to provide support for the physiological relevance of the main findings. Please also ensure to address all other comments of the reviewers, and adequately expand the discussion, as suggested.

Please be sure to address/respond to all concerns of the referees in full in a point-by-point response and highlight all changes in the revised manuscript text file. If you have comments that are intended for editors only, please include those in a separate cover letter.

We are committed to providing a fair and constructive peer-review process. Do not hesitate to contact us if there are specific requests from the reviewers that you believe are technically impossible or unlikely to yield a meaningful outcome.

We expect to see your revised manuscript within 2-3 months. If you cannot send it within this time, please contact us to discuss an extension; we would still consider your revision, provided that no similar work has been accepted for publication at NSMB or published elsewhere.

As you already know, we put great emphasis on ensuring that the methods and statistics reported in our papers are correct and accurate. As such, if there are any changes that should be reported, please submit an updated version of the Reporting Summary along with your revision.

Please follow the links below to download these files:

Reporting Summary:
https://www.nature.com/documents/nr-reporting-summary.pdf

Please note that the form is a dynamic 'smart pdf' and must therefore be downloaded and completed in Adobe Reader.

When submitting the revised version of your manuscript, please pay close attention to our href="https://www.nature.com/nature-portfolio/editorial-policies/image-integrity">Digital Image Integrity Guidelines. and to the following points below:

-- that unprocessed scans are clearly labelled and match the gels and western blots presented in figures. Please note that all key data shown in the main figures as cropped gels or blots should be presented in uncropped form, with molecular weight markers. While these data can be displayed in a relatively informal style, they must refer back to the relevant figures. These data should be submitted as source data with the last revision, prior to acceptance.
-- that control panels for gels and western blots are appropriately described as loading on sample processing controls
-- all images in the paper are checked for duplication of panels and for splicing of gel lanes.
-- For any revision that includes light microscopy data, we ask our authors to please include a completed light microscopy reporting table [https://www.nature.com/documents/Light_microscopy_reporting_table.xlsx] to ensure the methods are described thoroughly. The table will be available to reviewers and ultimately published should the manuscript be accepted at the journal.

EXTENDED DATA FIGURES

When re-submitting your manuscript, please ensure that any supplementary figures and tables that are crucial to the manuscript's conclusions are converted into Extended Data figures and tables to increase visibility of these data. Extended Data figures and tables are online-only (present in the online PDF and full-text HTML versions of the paper), peer-reviewed display items that provide essential background to the article but are not included in the main article due to space constraints. A maximum of ten Extended Data display items (figures and tables) is permitted.

Finally, please ensure that you retain unprocessed data and metadata files after publication, ideally archiving data in perpetuity, as these may be requested during the peer review and production process or after publication if any issues arise.

If there are additional or modified structures presented in the final revision, please submit the corresponding PDB validation reports.

SOURCE DATA: we urge authors to provide, in tabular form, the data underlying the graphical representations used in figures. This is to further increase transparency in data reporting, as detailed in this editorial (http://www.nature.com/nsmb/journal/v22/n10/full/nsmb.3110.html). Spreadsheets can be submitted in excel format. Only one (1) file per figure is permitted; thus, for multi-paneled figures, the source data for each panel should be clearly labeled in the Excel file; alternately the data can be provided as multiple, clearly labeled sheets in an Excel file. When submitting files, the title field should indicate which figure the source data pertains to. We encourage our authors to provide source data at the revision stage, so that they are part of the peer-review process.

Data availability: this journal strongly supports public availability of data. All data used in accepted papers should be available via a public data repository, or alternatively, as Supplementary Information. If data can only be shared on request, please explain why in your Data Availability Statement, and also in the correspondence with your editor. Please note that for some data types, deposition in a public repository is mandatory - more information on our data deposition policies and available repositories can be found below:
https://www.nature.com/nature-research/editorial-policies/reporting-standards#availability-of-data

We require deposition of coordinates (and, in the case of crystal structures, structure factors) into the Protein Data Bank with the designation of immediate release upon publication (HPUB). Electron microscopy-derived density maps and coordinate data must be deposited in EMDB and released upon publication. Deposition and immediate release of NMR chemical shift assignments are highly encouraged. Deposition of deep sequencing and microarray data is mandatory, and the datasets must be released prior to or upon publication. To avoid delays in publication, dataset accession numbers must be supplied with the final accepted manuscript and appropriate release dates must be indicated at the galley proof stage.

While we encourage the use of color in preparing figures, please note that this will incur a charge to partially defray the cost of printing. Information about color charges can be found at http://www.nature.com/nsmb/authors/submit/index.html#costs

Nature Structural & Molecular Biology is committed to improving transparency in authorship. As part of our efforts in this direction, we are now requesting that all authors identified as 'corresponding author' on published papers create and link their Open Researcher and Contributor Identifier (ORCID) with their account on the Manuscript Tracking System (MTS), prior to acceptance. This applies to primary research papers only. ORCID helps the scientific community achieve unambiguous attribution of all scholarly contributions. You can create and link your ORCID from the home page of the MTS by clicking on 'Modify my Springer Nature account'. For more information please visit please visit <a

href="http://www.springernature.com/orcid">www.springernature.com/orcid.

Please use the link below to submit your revised manuscript and related files:

Link Redacted

Note: This URL links to your confidential home page and associated information about manuscripts you may have submitted, or that you are reviewing for us. If you wish to forward this email to co-authors, please delete the link to your homepage.

We look forward to seeing the revised manuscript and thank you for the opportunity to review your work.

Sincerely,

Katarzyna Ciazynska, PhD
(she/her)
Senior Editor
Nature Structural & Molecular Biology
https://orcid.org/0000-0002-9899-2428

Referee expertise:

Referee #1: membrane protein biogenesis and QC, cryo-EM

Referee #2: membrane protein biogenesis and QC, cryo-EM

Referee #3: membrane protein biogenesis and QC, cryo-EM

Reviewers' Comments:

Reviewer #1 (Remarks to the Author):

The manuscript by Klose et al. presents a break-through structural study of the yeast EMC in a previously unanticipated complex with the Spf1 P5A-ATPase. Using a combination of co-immunoprecipitation (co-IP) and mass spectrometry, the authors discover that EMC/Spf1 forms a stable complex at endogenous levels, and that the analogous EMC/ATP13A1 complex is conserved in human cells. They then solve a cryo-EM structure of the recombinant EMC/Spf1 complex at high-resolution, and verify that a low-resolution structure of the endogenous yeast complex has the same organisation. This apo structure reveals a juxtaposition of the EMC insertase and Spf1 dislocase sites, a pronounced kink in the putative membrane plane and an interaction interface formed by the luminal domains, the importance of which is verified by co-IPs of mutagenised subunits. An AlphaFold3 model for the EMC/ATP13A1 complex suggests a similar mode of interaction, which is characterised further by the same co-IP approach. Lastly, the authors solve cryo-EM structures of the EMC/Spf1 complex with ATPgS and AlF4- to provide models for the E1-ATP and E1-P state of Spf1 respectively. They obtain additional density for the Spf1 arm in the E1-ATP state, which provides an additional stabilising interface with the cytosolic domains of the EMC. Overall, the results are of high interest, providing pioneering evidence that TMD insertase and dislocase activity can be combined into single supercomplex. The experimental data are compelling and the manuscript is well-presented, so my enthusiasm for publication is high, pending a few points to be addressed:

1. The quality of the cryo-EM maps is high (and I appreciate the ChimeraX session for reviewers), nevertheless I think there should be a figure showing snapshots of model fit to density, particularly for the relevant luminal dock and Spf1 arm interfaces, and regions that are resolved additionally or significantly better than in previous structures.

2. Page 6: “the cytoplasmic cap…was tilted and displaced” - it’s not clear whether the authors think this is due to the Spf1 (arm) interaction?

3. Page 6: “and it is also implicated in the insertion of TMDs separated by only short hydrophilic loops.” I would remove this statement as there is no experimental evidence, rather a suggestion from the cited review based on the absence of the GEL complex in yeast.

4. From the panels in Fig. 2 and Extended Data Fig. 4, it is hard to see that the “functional sites for substrate engagement directly face each other”. I would suggest to show a panel where the substrate binding sites are highlighted on the model/map in the same colour.

5. Even in the apo map, it seems like the Emc4 gate is better resolved than in previous structures, suggesting it is stabilised in the observed conformation. The position in the E1-ATP map is not much different despite having better resolved cytosolic features. Is the Emc3 hydrophilic groove accessible for substrate insertion in this conformation? Do the authors still envisage the gate is dynamic in the context of the complex with Spf1? It would be worth to add these points to the text.

6. On page 10: although the authors say "the structural prediction suggests an extended intramembrane interface", they do not directly comment on the more planar orientation of the modelled human complex compared to the yeast complex. What I assume is the ATP13A1 arm is also quite prominent in the AlphaFold3 model shown in Fig. 4b and Extended Fig. 6a. Does it have a sensible conformation/interface with EMC? Would you expect the arm/EMC interaction to be conserved in humans and would the human complex have to orient differently to form this interaction?

7. Extended Data Fig. 7: what are the cross-linked species formed between EMC10 wt and EMC7 M168C or P172C? They appear at the same molecular weight as the cross-link with EMC10 Y230C and L238C – does this not suggest cross-linking with the naturally occurring cysteines?

8. Page 10: "we observed substantial conformational heterogeneity of Spf1 in our map of the endogenous EMC:Spf1 complex". Was this heterogeneity not observed for the recombinant complex? Are the complexes bound to the nucleotide analogues more homogeneous? The processing strategies and pipelines are quite different for the various structures and could lead to different conclusions about heterogeneity; what was the rationale for this?

9. On a similar note, how do the two conformations shown in Fig. 5a compare to the apo complex? Does this complex essentially have the same conformation as the E1-P state or are there differences?

10. Page 10: currently there is no mention of whether the nucleotide analogues are resolved in the maps, the position of their binding site with respect to the Spf1 arm and how changes at the nucleotide binding site might be correlated with the stabilisation of the arm domain. These points would make a valuable addition to the text.

Reviewer #2 (Remarks to the Author):

In this manuscript, Klose et al. report a cryo-EM structure of the yeast EMC:Spf1 complex. The EMC is a conserved ER-membrane complex that mediates the insertion of certain transmembrane helices, particularly those located near the N- and C-termini. Spf1 (ATP13A1 in mammals) is a P-type ATPase that has been shown to function as a dislocase for mis-inserted transmembrane helices at the ER. In this sense, EMC and Spf1 act in the same general pathway but in opposite directions. The authors present a structure suggesting direct complex formation between the two, raising the interesting possibility that Spf1 serves as a proofreading factor for EMC-mediated membrane insertion. Using AlphaFold modeling, the authors further propose that the human EMC and ATP13A1 may form a similar complex although the interaction surfaces are not conserved. Mutagenesis analysis indicates that mutations in these sites reduce co-immunoprecipitation efficiency in both yeast and human complexes.
While the model is interesting and, in principle, plausible, its physiological significance remains uncertain. The manuscript lacks functional experiments beyond binding assays, leaving the proposed mechanism largely speculative. Recent reports of EMC interactions with BOS (ref. 12), CaV (ref. 17), and VDAC (ref. 55)—the latter likely an artifact given VDAC's mitochondrial localization, raise concerns about the "stickiness" of EMC, whether this may reflect a chaperone-like role or experimental artifact from the use of detergent. In this regard, Spf1 could simply represent another EMC client, rather than a proofreading factor. Without addressing these issues, the model remains speculative, and new insights are limited.

1. Biological significance of EMC:Spf1 formation: Do interaction-disrupting mutants affect the function of Spf1? There are several assays established for Spf1, for example, mistargeting mitochondrial proteins (e.g., Pex15Δ30 and Gem1) to the ER. Do the mutants fail to rescue mis-localization phenotypes?
2. Did the authors test AlphaFold predictions for the yeast complex? Does it produce consistent results that agree with the cryo-EM structure?
3. AlphaFold was used to generate a model for the human EMC:ATP13A1 complex. The authors should show the PAE matrix and report structural variability across multiple predicted models, particularly in the interaction regions.

Minor point:
This term "shared cavity" is misleading. EMC and Spf1 each have distinct cavities. In the complex, there is a sizeable gap between the two cavities, across which the substrate may diffuse.

Reviewer #3 (Remarks to the Author):

Summary:

In this study, the authors demonstrate that the EMC and Spf1/ATP13A1 form a stable, evolutionarily conserved supercomplex in the eukaryotic ER that physically links membrane protein insertase and dislocase activities. Their cryo-EM structures show EMC and Spf1 form a composite intramembrane cavity, and the authors suggest that this cavity enables coordinated substrate engagement, topogenesis, and error correction. They also suggest that ATP-driven conformational changes of Spf1 control cavity access for protein insertion or extraction. Key interactions between the two proteins are mediated by accessory subunits Emc7 and Emc10, confirmed by mutagenesis, with structural conservation across yeast and human validated by modeling using AlphaFold3. Together, the findings establish a new paradigm for membrane protein quality control in eukaryotes, integrating opposing activities (protein insertion and removal) through a unified molecular machine.

General Comments:

This is a well-written and clearly presented study supported by strong experimental evidence. The work addresses an important and interesting question regarding the interaction between the insertase EMC and the dislocase Spf1. The structure determination of this complex in multiple conformations is a significant and exciting achievement, as it provides new insights such as the identification of shared substrate-binding cavities between the two proteins and the observation of nucleotide-dependent conformational changes.
That said, some parts of the manuscript require clearer explanation and improved figures, especially the description of the shared cavity, which represents one of the major findings in this study. The introduction and discussion could also be expanded to better situate the authors' findings within the broader context.
Below are specific comments organized in their order of appearance in the manuscript.

Introduction:

• Introduction is generally well written, but the discussion of what is known about EMC and Spf1 is rather brief. A concise overview of the current state of knowledge from prior structural and biochemical studies would strengthen the introduction (without going into lots of detail, which is always a challenge, of course). Although some of this background is mentioned later in the results and discussion, presenting it here would help readers better appreciate the context of the work. For example, a brief description of EMC's subunit composition, the roles of individual units, and the current model of its mechanism would be valuable. A similar point applies to Spf1. Summarizing the current understanding of Spf1's substrate recognition and dislocation mechanism would add valuable context.

•"EMC…serves as a central hub in membrane protein biogenesis and quality control by interacting with multiple further quality control factors" – Please provide some examples.

Results:

•Across all structures determined, it is not clear whether the nucleotide density is well resolved or whether nucleotides were modeled (the provided models suggest they are not). For example, did the authors observe clear density for ATPγS? If not, it should be explicitly stated, and how they determined that the reconstructed class corresponds to a given nucleotide state rather than another.

•The composite intramembrane cavity shared by EMC and Spf1 needs to be more clearly described both in text and figures. In the figures, the approximate locations of the substrate-binding sites for EMC and Spf1 are indicated, but the shape and properties of these sites are difficult to discern. It may help to represent the full structure in cartoon mode but highlight the substrate-binding sites in surface representation (or another visualization strategy) to make them more apparent. From the provided figures, it is also difficult to see where the positively charged selective filters are located in both proteins. It would also be informative to show an electrostatic map and/or the residues that function as the selective filters.

•The conformational change of the Spf1 arm in the ATPγS-bound state is particularly interesting. Can the authors provide structural insights into the basis of this conformational change, specifically, how ATP binding drives the movement of the arm? In addition, it is not entirely clear from the figures where the nucleotide-binding site is located.

•Several structural changes are noted, such as the tilted cytoplasmic cap and bent membranes, but their structural or functional implications (observed or predicted) are not discussed. For example, does the tilted cytoplasmic cap seem to alter access to the cavity? Since EMC has been proposed to catalyze membrane protein insertion by inducing membrane thinning, could the observed membrane bending have a related effect? Do the authors observe membrane thinning around EMC in their structures?

Discussion:

•Since EMC is known to interact with multiple other factors, do the authors view the EMC–Spf1 interaction as transient or constitutive? How might EMC accommodate interactions with several different partners?

•Are there similarities or differences compared to Msp1 that would be informative to discuss?

•As noted above, some structural observations (e.g., cytoplasmic cap conformation, membrane bending) are described but not discussed in terms of potential functional implications. Expanding on these points would strengthen the discussion.

•Is anything known in the literature about the effects of EMC7/10 deletion? For instance, does it lead to reduced quality control, which would support the authors' findings and their hypothesis that this may result from diminished Spf1 interaction?

•What is known about the triggers for ATP hydrolysis by Spf1, which would shift the complex from a gated to an ungated form? Conversely, what drives Spf1 to bind ATP and block the gate?

Figures:

•Figure1A & Figure 2A --> in the schematic, please label EMC, Spf1, the tag, the antibody

•Figure 1C --> please label the peaks on the chromatogram and indicate the corresponding lanes on the SDS-PAGE

Version 2:

Decision Letter:

Nature Structural & Molecular Biology NSMB-A51530B

4th Mar 2026

Dear Dr. Feige,

Thank you for submitting your manuscript, "Structural basis of an endoplasmic reticulum EMC:Spf1 insertase-dislocase complex". Please accept our apologies for the delay in this decision. Nevertheless, the comments of 3 expert referees who re-reviewed the work are below.

You will see that while the reviewers appreciate the efforts put into the revision, reviewer #2 brings up persistent concerns. Specifically, while they acknowledge the newly added experimental data to solidify the biological relevance presented in the study, they ultimately remain unconvinced regarding the physiological role of EMC:Spf1 complex. Unfortunately, based on these comments, we cannot offer to publish the study in Nature Structural & Molecular Biology. We hope the referees' comments will be useful to you in revising the manuscript for submission elsewhere.

However, considering the strengths of the work, and the extent of the current support, we consulted our editorial colleagues at Nature Commutations. The editors at Nature Communications are interested in publishing the work, pending the final revision to address the remaining suggestions of referee #2 and reformatting to match the journal style. They offer to accept the manuscript in principle should you transfer there. Depending on the extent of your revisions, they might be able to assess this final version in house, without consulting reviewers again, but they do reserve the right to consult one or more referees again should your changes require an external expert assessment. If you have any queries, Katarzyna Marcinkiewicz (katarzyna.marcinkiewicz@us.nature.com) will be the handling editor at Nature Communications.

I am sincerely sorry we could not be more positive on this occasion. I understand that this decision will come as a disappointment, and I thank you for the opportunity to consider this work at NSMB.

Sincerely,
Kat

Katarzyna Ciazynska, PhD
(she/her)
Senior Editor
Nature Structural & Molecular Biology
https://orcid.org/0000-0002-9899-2428

Reviewer #1 (Remarks to the Author):

The authors have addressed my previous comments and the manuscript is ready for publication.

Reviewer #2 (Remarks to the Author):

In the initial review, this reviewer expressed concerns about the physiological significance of the observed EMC:Spf1 supercomplex. In response, the authors performed additional experiments testing the effects of EMC:Spf1 interface disruption on Spf1's functions. The results show that the EMC-interaction mutant (luminal dock mutant E1087A) of Spf1 is fully functional for the dislocation of well-studied TA client proteins Gem1 and Pex10Δ30, comparable to WT Spf1. The authors also found that the mutants stabilize two additional clients, Cst26 and Om14, similarly to WT Spf1. Together, these results indicate that the EMC:Spf1 interaction is not necessary for Spf1's functions on these clients.

The authors also extended their functional analysis to the stability of Erg9 and Gnp1. In the Erg9 assay, the E1087A mutant behaved like WT Spf1. Interestingly, the arm mutant (R866E/W866E/N868D/Q869E) exhibited a hypermorphic phenotype

(performing better than WT Spf1), the mechanism of which remains unclear. In the Gnp1 assay, both Spf1 mutants were also largely active like WT, though mild defects were observed under stress conditions (+DTT or terbinafine).

While this reviewer greatly appreciates the authors' efforts in these revision experiments, the overall functional effects of EMC:Spf1 disruption appear weak. It remains unknown how stable this supercomplex is in native membranes, and the current evidence suggests that EMC and Spf1 can each perform their known functions well independently. While the observed structures are interesting, the physiological significance of the supercomplex appears limited.

Specific points:

1. The abstract must state that disruption of the EMC:Spf1 supercomplex does not cause functional defects.
2. Given the speculative nature of the current model, Figure 6 should be removed. Alternatively, the figure could be balanced by adding a panel depicting the canonical model of EMC and Spf1 performing their respective functions independently, and moved to the Extended Data Figures.
3. Fig. 5e and f: The authors should confirm by western blotting that Spf1 protein levels in yeast are comparable across mutants and under tested conditions.

Reviewer #3 (Remarks to the Author):

The authors have appropriately addressed the reviewer’s comments and concerns. The revisions have improved the manuscript, particularly in terms of clarity, figure presentation, and the discussion of structural and mechanistic interpretations. The additional explanations regarding the composite cavity, nucleotide states, and the functional implications of the observed conformational changes are especially helpful. I appreciate the authors’ careful and thoughtful responses, and I believe the manuscript is now suitable for publication.

** As a service to authors, Springer Nature Limited provides authors with the ability to transfer a manuscript that one journal cannot offer to publish to another journal, without the author having to upload the manuscript data again. To transfer your manuscript to another NPG journal using this service, please click on Link Redacted

** For Springer Nature Limited general information and news for authors, see http://npg.nature.com/authors.

Version 3:

Decision Letter:

12th May 2026

Dear Dr. Feige,

Thank you for your letter concerning your manuscript "Structural basis of an endoplasmic reticulum EMC:Spf1 insertase-dislocase complex". We have now had a chance to discuss the points you raised in detail, and we have decided to send your paper back to the reviewers.

In this case, we will ask reviewers #1 and #3 to weigh in on your responses to reviewer #2.

Before we can do it, we ask that you ensure that all necessary data and materials are available for re-review of this manuscript version. This includes final PDB validation reports, Reporting summary and the source data.

1. REPORTING SUMMARY: Please provide an updated file:

https://www.nature.com/documents/nr-reporting-summary.pdf

This form is a dynamic ‘smart pdf’ and must be downloaded and completed in Adobe Reader.

2. REPORTING OF STRUCTURAL DATA: If there are additional or modified structures in the revision, please submit the corresponding PDB validation reports with your other files, and use the figshare integration system to provide access to maps, half-maps, and models.

Manuscripts reporting new structures should contain a table summarizing structural and refinement statistics. Please include these tables for new cryo-EM or -ET (https://www.nature.com/documents/nr-tables-cryo-em.doc and modifying this file for ET), NMR (https://www.nature.com/documents/nr-tables-nmr.doc) and X-ray crystallography (https://www.nature.com/documents/nr-tables-xray.doc). To facilitate assessment of the quality of the structural data, a stereo image of a portion of the electron density map (for crystallography papers) or of the superimposed lowest energy structures (>10; for NMR papers) should be provided with the submitted manuscript. If the reported structure represents a novel overall fold, a stereo image of the entire structure (as a backbone trace) should also be provided. For cryo-EM structures, a representative micrograph showing individual particles should be provided in the figures alongside a processing workflow (please see our Editorial for more information: https://www.nature.com/articles/s41594-025-01567-9).

DATA DEPOSITION: We require deposition of coordinates (and, in the case of crystal structures, structure factors) into the Protein Data Bank with the designation of immediate release upon publication (HPUB). Electron microscopy-derived density maps and coordinate data must be deposited in EMDB and released upon publication. Deposition and immediate release of NMR chemical shift assignments are requested. To avoid delays in publication, dataset accession numbers must be supplied with the revised manuscript and appropriate release dates must be indicated at the galley proof stage.

3. REPORTING OF LIGHT MICROSCOPY DATA: For any revision that includes light microscopy data, we ask our authors to please include a completed light microscopy reporting table [https://www.nature.com/documents/Light_microscopy_reporting_table.xlsx] to ensure the methods are described thoroughly. The table will be available to reviewers and

ultimately published, should the manuscript be accepted at the journal. When submitting the revised version of your manuscript, please pay close attention to our href="https://www.nature.com/nature-portfolio/editorial-policies/image-integrity">Digital Image Integrity Guidelines.

4. CODE AND COMPUTATIONAL WORK: If newly developed, unpublished code was used in this work, it must be provided with the revision and access to it must be disclosed in the Code Availability Statement in the manuscript. Please also provide the completed software submission checklist. This form is a dynamic ‘smart pdf’ and must be downloaded and completed in Adobe Reader, instead of opening it in a web browser: https://www.nature.com/documents/nr-software-policy.pdf

If molecular dynamics (MD) simulations were performed, please refer to this Editorial and provide a completed MD simulations checklist with your revision: https://www.nature.com/articles/s42003-023-04653-0

Lastly, if it applies, please complete and upload the completed machine learning checklist with your submission for re-review: https://www.nature.com/documents/machine-learning-checklist.pdf

5. SOURCE GEL IMAGES: Unprocessed scans should be provided for all gels and western blots presented in figures and should be clearly labelled with the figure number in the file title. The source gel images should be uncropped, unmodified images, with molecular weight markers. Uncropped, unmodified gel images from existing experimental repeats, in addition to the data shown in the figures, can also be presented in source data. Please provide these files as PDF with your revision.

Please ensure that all control panels for gels and western blots are appropriately described as loading or IP controls. Lastly, please ensure that all images in the paper are checked for duplication of panels and for splicing of gel lanes.

6. SOURCE NUMERICAL DATA: we urge authors to provide, in tabular form in excel files, the data underlying the graphical representations used in figures. This is to further increase transparency in data reporting, as detailed in this editorial (http://www.nature.com/nsmb/journal/v22/n10/full/nsmb.3110.html). Please provide one excel file per figure max, with one panel per tab. When submitting files, the title field should indicate which figure the source data pertains to.

7. DATA AVAILABILITY: this journal strongly supports public availability of data. All data used in accepted papers should be available via a public data repository, or alternatively, as Supplementary Information. If data can only be shared on request, please explain why in your Data Availability Statement and in the correspondence with your editor.

Once we receive this document and review it to ensure that all requested information is provided, we will proceed to send your paper for review. If you have questions or anticipate delays, please let me know as soon as possible.

You can use the link below to be taken directly to the site and submit your manuscript:

Link Redacted

Sincerely,

Katarzyna Ciazynska, PhD
(she/her)
Senior Editor
Nature Structural & Molecular Biology
https://orcid.org/0000-0002-9899-2428

Version 4:

Decision Letter:

Our ref: NSMB-A51530D

5th Jun 2026

Dear Dr. Feige,

Thank you for submitting your revised manuscript "Structural basis of an endoplasmic reticulum EMC:Spf1 insertase-dislocase complex" (NSMB-A51530D). As you know, we have editorially considered the points you made in response to comments of Reviewer #2 and decided to consult reviewers #1 and #2 on your responses. The manuscript has now been seen again by Reviewers #1 and #3, who provided additional input, which you can see below.

The reviewers find the extent to which the data in the manuscript support the main conclusions appropriate, even in light of the criticism of Reviewer #2. We will therefore be happy in principle to publish it in Nature Structural & Molecular Biology, pending minor revisions to satisfy the referees' final requests and to comply with our editorial and formatting guidelines.

We are now performing detailed checks on your paper and will send you a checklist detailing our editorial and formatting requirements in about a week. Please do not upload the final materials and make any revisions until you receive this additional information from us.

Thank you again for your interest in Nature Structural & Molecular Biology Please do not hesitate to contact me if you have any questions.

Sincerely,
Kat

Katarzyna Ciazynska, PhD
(she/her)
Senior Editor
Nature Structural & Molecular Biology
https://orcid.org/0000-0002-9899-2428

Reviewer #1 (Remarks to the Author):

I have re-evaluated the manuscript by Klose et al., with regards to the comments from reviewer 2 and the author responses. Overall, I am still of the opinion that the authors present compelling evidence for a novel and exciting supercomplex between EMC and Spf1 that is ready for publication. I think the additional experiments that have been included in both revisions have addressed the specific points of reviewer 2 and indicate physiological relevance of the complex. Further functional studies targeting physiological significance are in my opinion beyond the necessary scope of this current work. I would specifically comment on the major concerns as follows:

1. From the initial review: “In this regard, Spf1 could simply represent another EMC client, rather than a proofreading factor.”

The authors perform the functional assays suggested by reviewer 2 for a variety of substrates. These clearly show that Spf1 is functional within the complex and I would agree this argues against a role of the complex in Spf1 biogenesis. Structurally, Spf1 binds to a distinct site in EMC from those typically occupied by clients like CaV i.e. the lipid-filled cavity or hydrophilic groove.

2. From the first review: “this may reflect an…..experimental artifact from the use of detergent” and from the second review: “it remains unknown how stable this supercomplex is in native membranes.”

I think this is the most critical point for concerns about ‘physiological relevance’. Whilst the manuscript largely deals with a complex that has been detergent-extracted from native membranes, in my extensive experience of purifying membrane

protein complexes for structural studies it is much more common a detergent destabilises a physiological complex rather than enforces a non-physiological one. The high stability and 1:1 stoichiometry of this complex (particularly when extracted at endogenous levels) together with accurate AlphaFold predictions of a novel complex, convince me this is a physiologically relevant complex. In addition, the authors show that mutations that disrupt the complex have some (albeit minor) affects on Spf1 function in native membranes and that the complex is maintained when reconstituted into a lipid environment from detergent.

3. From the second review: “the overall functional effects of EMC:Spf1 disruption appear weak” and “current evidence suggests that EMC and Spf1 can each perform their known functions well independently”

I agree with these statements but don’t understand why this is detrimental to the manuscript. As the authors explain, biogenesis and quality control pathways at the ER are often redundant and compensatory – indeed, they now show in SI Fig. 1c that knockouts of either EMC or Spf1 have modest effects on the proteome, therefore it is unsurprising the more subtle point mutations to the supercomplex have mild affects. Whilst their data show that EMC and Spf1 core functions do not require complex formation, the supercomplex could have an alternative role such as boosting the efficiency of protein quality control. To this end, the authors show that disruption of EMC:Spf1 has more pronounced effects on Gnp1 stability under stress conditions i.e. when the need for efficient protein quality control at the ER is at its greatest.

Reviewer #3 (Remarks to the Author):

After reviewing the revised manuscript and the authors’ responses to Reviewer #2’s concerns regarding the strength and physiological significance of the EMC association, I find the authors’ arguments convincing and overall support publication of the study.

In my opinion, the biochemical and structural evidence strongly supports the existence of a specific and physiologically relevant complex. It is generally unlikely for a non-specific interaction to survive the extensive purification workflow used here, involving affinity purification followed by size-exclusion chromatography, while remaining sufficiently homogeneous for high-resolution cryo-EM structure determination. The fact that the authors were able to obtain well resolved structures of the complex argues strongly against this being a transient detergent artifact or non specific association.

Regarding the relatively modest physiological phenotypes observed upon disruption of the interaction, I agree with the authors’ interpretation. Membrane protein biogenesis and quality control pathways are fundamentally important cellular processes and are therefore supported by multiple overlapping and partially redundant systems. As a result, disruption of a single interaction or pathway component often produces only subtle or context dependent phenotypes, (particularly under standard laboratory growth conditions).

Overall, I believe the revised manuscript provides compelling structural and biochemical evidence for the EMC supercomplex and offers important conceptual insight into how membrane protein biogenesis and quality control machineries may be spatially organized within the ER membrane.

Version 5:

Decision Letter:

12th Aug 2026

Dear Professor Feige,

We are now happy to accept your revised paper "Structural basis of an endoplasmic reticulum EMC:Spf1 insertase-dislocase complex" for publication as an Article in Nature Structural & Molecular Biology.

Acceptance is conditional on the manuscript's not being published elsewhere and on there being no announcement of this work to the newspapers, magazines, radio or television until the publication date in Nature Structural & Molecular Biology.

Over the next few weeks, your paper will be copyedited to ensure that it conforms to Nature Structural & Molecular Biology style. Once your paper is typeset, you will receive an email with a link to choose the appropriate publishing options for your paper and our Author Services team will be in touch regarding any additional information that may be required.

After the grant of rights is completed, you will receive a link to your electronic proof via email with a request to make any corrections within 48 hours. If, when you receive your proof, you cannot meet this deadline, please inform us at rjsproduction@springernature.com immediately.

You will not receive your proofs until the publishing agreement has been received through our system.

Due to the importance of these deadlines, we ask that you please let us know now whether you will be difficult to contact over the next month. If this is the case, we ask you provide us with the contact information (email, phone and fax) of someone who will be able to check the proofs on your behalf, and who will be available to address any last-minute problems.

To assist our authors in disseminating their research to the broader community, our SharedIt initiative provides all co-authors with the ability to generate a unique shareable link that will allow anyone (with or without a subscription) to read the published article. Recipients of the link with a subscription will also be able to download and print the PDF.

As soon as your article is published, you can generate your shareable link by entering the DOI of your article here: http://authors.springernature.com/share. Corresponding authors will also receive an automated email with the shareable link

Note the policy of the journal on data deposition: http://www.nature.com/authors/policies/availability.html.

Your paper will be published online soon after we receive proof corrections and will appear in print in the next available issue. You can find out your date of online publication by contacting the production team shortly after sending your proof corrections.

You may wish to make your media relations office aware of your accepted publication, in case they consider it appropriate to organize some internal or external publicity. Once your paper has been scheduled you will receive an email confirming the publication details. This is normally 3-4 working days in advance of publication. If you need additional notice of the date and time of publication, please let the production team know when you receive the proof of your article to ensure there is sufficient time to coordinate. Further information on our embargo policies can be found here: https://www.nature.com/authors/policies/embargo.html

You can now use a single sign-on for all your accounts, view the status of all your manuscript submissions and reviews, access usage statistics for your published articles and download a record of your refereeing activity for the Nature journals.

If you have not already done so, we strongly recommend that you upload the step-by-step protocols used in this manuscript to the Protocol Exchange. Protocol Exchange is an open online resource that allows researchers to share their detailed experimental know-how. All uploaded protocols are made freely available, assigned DOIs for ease of citation and fully searchable through nature.com. Protocols can be linked to any publications in which they are used and will be linked to from your article. You can also establish a dedicated page to collect all your lab Protocols. By uploading your Protocols to Protocol Exchange, you are enabling researchers to more readily reproduce or adapt the methodology you use, as well as increasing the visibility of your protocols and papers. Upload your Protocols at www.nature.com/protocolexchange/. Further information can be found at www.nature.com/protocolexchange/about.

An online order form for reprints of your paper is available at https://www.nature.com/reprints/author-reprints.html. Please let your coauthors and your institutions' public affairs office know that they are also welcome to order reprints by this method.

Authors may need to take specific actions to achieve compliance with funder and institutional open access mandates. If your research is supported by a funder that requires immediate open access (e.g. according to Plan S principles or the NIH public access policy) then you should select the gold OA route, and we will direct you to the compliant route where possible. Because authors warrant under our subscription licensing terms that they haven't committed to licensing any version of their article under a licence inconsistent with the terms of our agreement – including the applicable embargo period – publication under the subscription model isn't suitable for authors whose funders require no embargo.

In approximately 10 business days you will receive an email with a link to choose the appropriate publishing options for your paper and our Author Services team will be in touch regarding any additional information that may be required.

You will not receive your proofs until the publishing agreement has been received through our system.

If you have any questions about our publishing options, costs, Open Access requirements, or our legal forms, please contact ASJournals@springernature.com

Sincerely,

Dimitris Typas
Senior Editor
Nature Structural & Molecular Biology
ORCID: 0000-0002-8737-1319

on behalf of

Katarzyna Ciazynska, PhD
(she/her)
Senior Editor
Nature Structural & Molecular Biology
https://orcid.org/0000-0002-9899-2428

Click here if you would like to recommend Nature Structural & Molecular Biology to your librarian:
http://www.nature.com/subscriptions/recommend.html#forms

Open Access This Peer Review File is licensed under a Creative Commons Attribution 4.0 International License, which permits use, sharing, adaptation, distribution and reproduction in any medium or format, as long as you give appropriate credit to the original author(s) and the source, provide a link to the Creative Commons license, and indicate if changes were made.
In cases where reviewers are anonymous, credit should be given to 'Anonymous Referee' and the source.
The images or other third party material in this Peer Review File are included in the article's Creative Commons license, unless indicated otherwise in a credit line to the material. If material is not included in the article's Creative Commons license and your intended use is not permitted by statutory regulation or exceeds the permitted use, you will need to obtain permission directly from the copyright holder.
To view a copy of this license, visit https://creativecommons.org/licenses/by/4.0/

Klose et al., reply to the reviewers' comments:

Reviewer #1 (Remarks to the Author):

The manuscript by Klose et al. presents a break-through structural study of the yeast EMC in a previously unanticipated complex with the Spf1 P5A-ATPase. Using a combination of co-immunoprecipitation (co-IP) and mass spectrometry, the authors discover that EMC/Spf1 forms a stable complex at endogenous levels, and that the analogous EMC/ATP13A1 complex is conserved in human cells. They then solve a cryo-EM structure of the recombinant EMC/Spf1 complex at high-resolution, and verify that a low-resolution structure of the endogenous yeast complex has the same organisation. This apo structure reveals a juxtaposition of the EMC insertase and Spf1 dislocase sites, a pronounced kink in the putative membrane plane and an interaction interface formed by the luminal domains, the importance of which is verified by co-IPs of mutagenised subunits. An AlphaFold3 model for the EMC/ATP13A1 complex suggests a similar mode of interaction, which is characterised further by the same co-IP approach. Lastly, the authors solve cryo-EM structures of the EMC/Spf1 complex with ATPgS and AlF4- to provide models for the E1-ATP and E1-P state of Spf1 respectively. They obtain additional density for the Spf1 arm in the E1-ATP state, which provides an additional stabilising interface with the cytosolic domains of the EMC. Overall, the results are of high interest, providing pioneering evidence that TMD insertase and dislocase activity can be combined into single supercomplex. The experimental data are compelling and the manuscript is well-presented, so my enthusiasm for publication is high, pending a few points to be addressed

We are delighted by the reviewer's interest for our study. We thank the reviewer for the helpful suggestions. Addressing the points raised has helped to further improve our manuscript.

1. The quality of the cryo-EM maps is high (and I appreciate the ChimeraX session for reviewers), nevertheless I think there should be a figure showing snapshots of model fit to density, particularly for the relevant luminal dock and Spf1 arm interfaces, and regions that are resolved additionally or significantly better than in previous structures.

We have added images of model fit to density for key structural regions (SI Figure 4).

2. Page 6: "the cytoplasmic cap…was tilted and displaced" - it's not clear whether the authors think this is due to the Spf1 (arm) interaction?

In the revised manuscript, we have adjusted the wording to better describe the observed changes on pages 6 and 19. Because this effect was also seen in the apo structure that did not resolve the arm, we do not think that this is only due to the newly formed interface. However, it is striking that this difference is consistent for the comparisons across all three published structures of the yeast EMC without Spf1. We would like to remain cautious about an interpretation for this structural observation, and thus we simply note that the entrance to the insertase cavity from the cytosol is relatively more accessible in the structures with Spf1. The same conformation of the EMC cap can also be observed in the ATPγS bound state (with Spf1), yet the arm is now blocking access to the cavity from the cytosol in a lid-like manner.

3. Page 6: “and it is also implicated in the insertion of TMDs separated by only short hydrophilic loops.” I would remove this statement as there is no experimental evidence, rather a suggestion from the cited review based on the absence of the GEL complex in yeast.

We thank the reviewer for pointing this out and have removed this statement from the text.

4. From the panels in Fig. 2 and Extended Data Fig. 4, it is hard to see that the “functional sites for substrate engagement directly face each other”. I would suggest to show a panel where the substrate binding sites are highlighted on the model/map in the same colour.

To address this, our revised manuscript now highlights this structural feature in Figure 2d and Extended Data Fig. 4d, 4e.

5. Even in the apo map, it seems like the Emc4 gate is better resolved than in previous structures, suggesting it is stabilised in the observed conformation. The position in the E1-ATP map is not much different despite having better resolved cytosolic features. Is the Emc3 hydrophilic groove accessible for substrate insertion in this conformation? Do the authors still envisage the gate is dynamic in the context of the complex with Spf1? It would be worth to add these points to the text.

We agree with the reviewer that even in the apo reconstruction, the Emc4 gate is better resolved than in previously reported EMC structures, suggesting stabilization of a defined conformation. Of note, to date, multiple distinct gate conformations were reported for the human EMC; only a single gate conformation was observed in all the three prior structures of the yeast complex. The position of the Emc4 gate in the E1–ATP map is also very similar to that in the apo map, despite improved resolution of cytosolic features. In this conformation, access to the Emc3 hydrophilic groove is restricted, as entry is occluded by the arm domain. Thus, any notions of gate flexibility in yeast are speculative, and we have adapted the discussion to account for this. As we now discuss on page 19 of the revised manuscript, this gate arrangement laterally delimits the composite cavity.

6. On page 10: although the authors say “the structural prediction suggests an extended intramembrane interface”, they do not directly comment on the more planar orientation of the modelled human complex compared to the yeast complex. What I assume is the ATP13A1 arm is also quite prominent in the AlphaFold3 model shown in Fig. 4b and Extended Fig. 6a. Does it have a sensible conformation/interface with EMC? Would you expect the arm/EMC interaction to be conserved in humans and would the human complex have to orient differently to form this interaction?

The Spf1/ATP13A1 arm domain is poorly conserved between different species (Li et al., 2024), and the sequence of the human arm is much shorter (by 52 residues) than that from yeast. So, if the same arm-EMC interface exists, this might explain why the orientation is not angled/tilted by the interactions mediated by the arm. However, AlphaFold3 predicts the same complex orientation if the human structure is predicted with or without the arm domain (data not shown), suggesting that the EMC-arm

interface does not determine the supercomplex architecture in humans. Furthermore, the nature of the human ATP13A1:EMC interaction is different to the yeast Spf1:EMC one (e.g. missing the lumenal dock, as our data show and as we describe in the manuscript), which may also explain the lack of membrane curvature in the human complex. We now more explicitly mention this on page 12 of the revised manuscript.

7. Extended Data Fig. 7: what are the cross-linked species formed between EMC10 wt and EMC7 M168C or P172C? They appear at the same molecular weight as the cross-link with EMC10 Y230C and L238C – does this not suggest cross-linking with the naturally occurring cysteines?

We think it is most likely that EMC7 crosslinks with another partner, because the crosslinked bands are not recognized by the antibody recognizing the FLAG-tag on the expressed EMC10 (although we cannot exclude that the crosslinks do exclusively form with endogenous EMC10 and not the transfected variant, but this seems unlikely). The bands could for example indicate crosslinking with another EMC subunit such as EMC4, in which case the IP based on the tagged EMC subunit (EMC10) would still isolate this complex. We added this information to the figure legend of Extended Data Fig. 7.

8. Page 10: "we observed substantial conformational heterogeneity of Spf1 in our map of the endogenous EMC:Spf1 complex". Was this heterogeneity not observed for the recombinant complex? Are the complexes bound to the nucleotide analogues more homogeneous? The processing strategies and pipelines are quite different for the various structures and could lead to different conclusions about heterogeneity; what was the rationale for this?

We fully agree that this was misleading and removed the statement from the revised manuscript.

9. On a similar note, how do the two conformations shown in Fig. 5a compare to the apo complex? Does this complex essentially have the same conformation as the E1-P state or are there differences?

To compare these different states, we low pass filtered all maps to a resolution of 10 Å, allowing more accurate assessment of domain positioning that is not biased by differences in resolution. From this we conclude that the changes observed in Spf1 positioning and size of the composite cavity are most pronounced when comparing the ATPγS- and AlF_4^--bound states. In the last consensus map of the apo state, before separate refinement of EMC and Spf1 (see SI Fig. 2 for data processing details), Spf1 occupies a position broadly similar to that observed in the E1-ATP state, albeit slightly closer to the EMC.

In our speculative model for an ATP-driven translocation cycle (Fig. 6), this apo state would correspond to the E1 conformation, where we propose client helix release into the membrane or cytoplasm. Hence, an increased distance between the EMC and Spf1 might facilitate such a release process.

We have added this new comparison to Extended Data Fig. 8c and added a respective statement on page 15.

10. Page 10: currently there is no mention of whether the nucleotide analogues are resolved in the maps, the position of their binding site with respect to the Spf1 arm and how changes at the nucleotide binding site might be correlated with the stabilisation of the arm domain. These points would make a valuable addition to the text.

In the ATPγS-bound state, we observe additional density in the nucleotide binding site clearly indicating the presence of a nucleotide in the unsharpened cryo-EM map. However, the resolution is not sufficient to precisely build this nucleotide analog in the structural model. The nucleotide analog was added in approximately 200-fold molar excess to the protein complex and we see a clear conformational change in comparison to the apo state, now resembling the published E1-ATP conformation in the presence of AMPPCP (PDB: 6XMQ) (McKenna et al., 2020). These features led us to surmise that this map represents the E1-ATP state. We have now added this to the Methods section. We also mention this clearly in the Results section on page 15, and show the density in the nucleotide-binding site in Extended Data Fig. 8a.

Similarly, the cryo-EM map acquired in the presence of AlF_4^- agrees with the previously reported structure of Spf1 reconsituted with the same nucleotide analog that defined the E1-P state (PDB: 6XMS) (McKenna et al., 2020). The resolution of our map does not allow to determine the presence or absence of a nucleotide. Therefore, we are cautious in the text about the nucleotide state of this map. We have added an additional statement to clarify this on page 15 of the revised manuscript.

Structurally, the arm domain is positioned in close proximity to key catalytic elements that undergo large rearrangements upon ATP binding and hydrolysis. Notably, the catalytic aspartate in the P-domain is located ~15 Å away from the N- and C-termini of the arm domain, and the conserved SGE dephosphorylation motif in the A-domain is positioned between these termini. Given that ATP binding and hydrolysis are known to drive substantial relative movements of the A- and N-domains in P-type ATPases, these rearrangements provide a plausible structural basis for an indirect, relay-like coupling to the arm domain, thereby driving its observed repositioning in the ATPγS-bound state. So while our data do not allow us to define the precise molecular pathway of this coupling, the spatial arrangement of the arm relative to the catalytic core supports a model where nucleotide-dependent domain motions are transmitted to the arm domain through coordinated movements of the A- and N-domains.

We have added a panel to Extended Data Fig. 8b to clarify the relative locations of these key catalytic elements, and have added a statement to the text on page 15 of the revised manuscript that explains the underlying rationale.

Reviewer #2 (Remarks to the Author):

In this manuscript, Klose et al. report a cryo-EM structure of the yeast EMC:Spf1 complex. The EMC is a conserved ER-membrane complex that mediates the insertion of certain transmembrane helices, particularly those located near the N- and C-termini. Spf1 (ATP13A1in mammals) is a P-type ATPase that has been shown to function as a dislocase for mis-inserted transmembrane helices at the ER. In this sense, EMC and Spf1 act in the same general pathway but in opposite directions. The authors present a structure suggesting direct complex formation between the two, raising the interesting possibility that Spf1 serves as a proofreading factor for EMC-mediated membrane insertion. Using AlphaFold modeling, the authors further propose that the human EMC and ATP13A1 may form a similar complex although the interaction surfaces are not conserved. Mutagenesis analysis indicates that mutations in these sites reduce co-immunoprecipitation efficiency in both yeast and human complexes. While the model is interesting and, in principle, plausible, its physiological significance remains uncertain. The manuscript lacks functional experiments beyond binding assays, leaving the proposed mechanism largely speculative. Recent reports of EMC interactions with BOS (ref. 12), CaV (ref. 17), and VDAC (ref. 55)—the latter likely an artifact given VDAC's mitochondrial localization, raise concerns about the "stickiness" of EMC, whether this may reflect a chaperone-like role or experimental artifact from the use of detergent. In this regard, Spf1 could simply represent another EMC client, rather than a proofreading factor. Without addressing these issues, the model remains speculative, and new insights are limited.

We are very pleased that the reviewer found our structural data and model interesting. We thank the reviewer for the helpful suggestions. Addressing the points has helped to further improve our manuscript.

1. Biological significance of EMC:Spf1 formation: Do interaction-disrupting mutants affect the function of Spf1? There are several assays established for Spf1, for example, mistargeting mitochondrial proteins (e.g., Pex15Δ30 and Gem1) to the ER. Do the mutants fail to rescue mis-localization phenotypes?

This indeed is a highly relevant point. To address this, we performed many new experiments, now shown in Fig. 3d, 5e, 5f and Extended Data Fig. 5e, 5f, 5g, 8d, 8e and mentioned in the results of our revised manuscript.

As suggested by the reviewer, we performed several established assays for Spf1 function: 1. sensitivity to caffeine in a growth assay (Ando & Suzuki, 2005) (Extended Data Fig. 5e) 2. mislocalization of the endogenous mitochondrial TA protein Gem1 as seen by fluorescence microscopy (Krumpe et al., 2012) (Extended Data Fig. 5g) and 3. stabilization of a Pex15Δ30 stability reporter in flow cytometry (Dederer et al., 2019) (Fig. 3d, Extended Data Fig 5f). In addition, we designed further protein stability reporters allowing flow cytometry based readouts for the Spf1 client Cst26 (Cohen et al., 2013) and the Spf1 and EMC interactor Om14 (Fig. 3d, Extended Data Fig. 5f).

To be able to test roles of Spf1 and EMC, and to examine effects of mutations, we generated ∆Spf1 and ∆Emc3 yeast strains, as well as a control strain where the same antibiotic resistance was integrated into the HO locus. These were then transformed

with a centromeric plasmid encoding for an Spf1 variant or an empty vector and then subjected to the respective assay.

All the abovementioned assays were insensitive to an Emc3 KO, indicating that they truly report only on Spf1 activity. Our Spf1 mutants that disrupt the interaction with the EMC (E1087A at the lumenal dock and R866E W867E N868D Q869E designated as “arm mutation”) showed a similar behavior to the strain with wildtype Spf1 in all of these Spf1 activity assays, in contrast to controls that eliminate Spf1 catalytic activity (D487A) or the unrescued Spf1 KO. This indicates that interaction with the EMC is not required for these functions of Spf1 and that an EMC-interaction-deficient mutant is still catalytically active. Furthermore, we conclude that the biogenesis of Spf1 is still successful when disrupting the interaction with the EMC and thus Spf1 is not dependant on the EMC for maturation as a client via our observed interactions.

Additionally, we assessed the effects of Spf1 mutations on two EMC client proteins in the flow cytometry-based protein stability assay: Erg9, the human ortholog of squalene synthase (SQS/FDFT1) and Gnp1, a multipass protein that was shown to be destabilized in the absence of the EMC. We now include this data in Fig. 5e, f and Extended Data Fig. 8d, e.

Interestingly, Erg9 is destabilized when the EMC:arm interface is mutated and stabilized in the context of inactive Spf1 (D487A) or an unrescued Spf1 KO. This data could suggest that Spf1 plays a role in dislocating Erg9 from the ER membrane and that this dislocation mechanism is misregulated when the EMC:arm interface is disrupted.

In contrast, there is a slight (5%) increase in Gnp1 stability in the strain with the arm mutation, indicating that this mutant cannot fully rescue the Spf1 KO phenotype. This effect is more pronounced when subjecting the cells to the stress of DTT. The lumenal dock mutation (E1087A) shows a similar stabilizing effect when ergosterol biosynthesis is inhibited by Terbinafine.

These data provide first insights into functional consequences of disrupting the EMC:Spf1 supercomplex for certain EMC/Spf1 co-clients but are far from a comprehensive or mechanistic understanding. We envision protein targeting and insertion as a dynamic process that is constantly surveilled and corrected by a large network of quality control factors. While the current picture of this network is far from complete, we speculate that manipulation of one player may result in a spectrum of different outcomes, depending on compensatory mechanisms. We attribute the opposite phenotypes observed with Erg9 and Gnp1 upon Spf1 KO or disruption of the EMC:Spf1 interaction to this unexplored complexity of the network and a not fully understood role of Spf1 and the EMC in the biogenesis of their clients. This is now also mentioned in the Results section as well as in the Discussion.

2. Did the authors test AlphaFold predictions for the yeast complex? Does it produce consistent results that agree with the cryo-EM structure?

As suggested, we now add an AlphaFold3 model of the yeast complex to SI Fig. 6 and mention this in the revised results section. The model is overall consistent with our experimental structure, predicting the same overall architecture of the supercomplex

as well as the lumenal dock as the major interaction interface. We note differences in the angular orientation of Spf1 towards the EMC and variability in the arm conformations (which we find to be nucleotide-regulated).

3. AlphaFold was used to generate a model for the human EMC:ATP13A1 complex. The authors should show the PAE matrix and report structural variability across multiple predicted models, particularly in the interaction regions.

The PAE matrix, model confidence, and structural variability are shown in SI Figure 5 of the revised manuscript.

Minor point:
This term “shared cavity” is misleading. EMC and Spf1 each have distinct cavities. In the complex, there is a sizeable gap between the two cavities, across which the substrate may diffuse.

We agree and thus have renamed this “composite intramembrane cavity” throughout the text.

Reviewer #3 (Remarks to the Author):

Summary:

In this study, the authors demonstrate that the EMC and Spf1/ATP13A1 form a stable, evolutionarily conserved supercomplex in the eukaryotic ER that physically links membrane protein insertase and dislocase activities. Their cryo-EM structures show EMC and Spf1 form a composite intramembrane cavity, and the authors suggest that this cavity enables coordinated substrate engagement, topogenesis, and error correction. They also suggest that ATP-driven conformational changes of Spf1 control cavity access for protein insertion or extraction. Key interactions between the two proteins are mediated by accessory subunits Emc7 and Emc10, confirmed by mutagenesis, with structural conservation across yeast and human validated by modeling using AlphaFold3. Together, the findings establish a new paradigm for membrane protein quality control in eukaryotes, integrating opposing activities (protein insertion and removal) through a unified molecular machine.

General Comments:
This is a well-written and clearly presented study supported by strong experimental evidence. The work addresses an important and interesting question regarding the interaction between the insertase EMC and the dislocase Spf1. The structure determination of this complex in multiple conformations is a significant and exciting achievement, as it provides new insights such as the identification of shared substrate-binding cavities between the two proteins and the observation of nucleotide-dependent conformational changes.
That said, some parts of the manuscript require clearer explanation and improved figures, especially the description of the shared cavity, which represents one of the major findings in this study. The introduction and discussion could also be expanded to better situate the authors' findings within the broader context.
Below are specific comments organized in their order of appearance in the manuscript.

We are delighted by the interest in our manuscript and thank the reviewer for the helpful suggestions. We have addressed all remaining concerns as outlined below.

Introduction:

Introduction is generally well written, but the discussion of what is known about EMC and Spf1 is rather brief. A concise overview of the current state of knowledge from prior structural and biochemical studies would strengthen the introduction (without going into lots of detail, which is always a challenge, of course). Although some of this background is mentioned later in the results and discussion, presenting it here would help readers better appreciate the context of the work. For example, a brief description of EMC's subunit composition, the roles of individual units, and the current model of its mechanism would be valuable. A similar point applies to Spf1. Summarizing the current understanding of Spf1's substrate recognition and dislocation mechanism would add valuable context.

Picking up this suggestion, while still aiming for a concise introduction, we have extended the background about EMC and Spf1 in the introduction, adding more on their architecture and current model of mechanism on pages 3 and 4.

“EMC…serves as a central hub in membrane protein biogenesis and quality control by interacting with multiple further quality control factors” – Please provide some examples.

In the revised manuscript, we now add some examples and references for these on page 3.

Results:

Across all structures determined, it is not clear whether the nucleotide density is well resolved or whether nucleotides were modeled (the provided models suggest they are not). For example, did the authors observe clear density for ATPγS? If not, it should be explicitly stated, and how they determined that the reconstructed class corresponds to a given nucleotide state rather than another.

In the ATPγS-bound state, we observe density in the nucleotide binding site clearly indicating the presence of a nucleotide in the unsharpened cryo-EM map. However, the resolution is not sufficient to precisely build this nucleotide analog in the structural model. The nucleotide analog was added in approximately 200-fold molar excess to the protein complex and we see a clear conformational change in comparison to the apo state, now resembling the published E1-ATP conformation in the presence of AMPPCP (PDB: 6XMQ) (McKenna et al., 2020). These features led us to surmise that this map represents the E1-ATP state. We have now added this to the Methods section. We also mention this clearly in the Results section on page 15, and show the density in the nucleotide-binding site in Extended Data Fig. 8a.

Similarly, the cryo-EM map acquired in the presence of AlF_4^- agrees with the previously reported structure of Spf1 reconsituted with the same nucleotide analog that defined the E1-P state (PDB: 6XMS) (McKenna et al., 2020). The resolution of our map does not allow to determine the presence or absence of a nucleotide. Therefore, we are cautious in the text about the nucleotide state of this map. We have added an additional statement to clarify this on page 15 of the revised manuscript.

The composite intramembrane cavity shared by EMC and Spf1 needs to be more clearly described both in text and figures. In the figures, the approximate locations of the substrate-binding sites for EMC and Spf1 are indicated, but the shape and properties of these sites are difficult to discern. It may help to represent the full structure in cartoon mode but highlight the substrate-binding sites in surface representation (or another visualization strategy) to make them more apparent. From the provided figures, it is also difficult to see where the positively charged selective filters are located in both proteins. It would also be informative to show an electrostatic map and/or the residues that function as the selective filters.

We have adapted our representation and description of the composite cavity in the revised Fig. 2d and Extended Data Fig. 4d, e, now showing the surface and electrostatic properties of both substrate binding sites more clearly.

The conformational change of the Spf1 arm in the ATPγS-bound state is particularly interesting. Can the authors provide structural insights into the basis of this conformational change, specifically, how ATP binding drives the movement of the arm? In addition, it is not entirely clear from the figures where the nucleotide-binding site is located.

Structurally, the arm domain is positioned in close proximity to key catalytic elements that undergo large rearrangements upon ATP binding and hydrolysis. Notably, the catalytic aspartate in the P-domain is located ~15 Å away from the N- and C-termini of the arm domain, and the conserved SGE dephosphorylation motif in the A-domain is positioned between these termini. Given that ATP binding and hydrolysis are known to drive substantial relative movements of the A- and N-domains in P-type ATPases, these rearrangements provide a plausible structural basis for an indirect, relay-like coupling to the arm domain, thereby driving its observed repositioning in the ATPγS-bound state. So while our data do not allow us to define the precise molecular pathway of this coupling, the spatial arrangement of the arm relative to the catalytic core supports a model where nucleotide-dependent domain motions are transmitted to the arm domain through coordinated movements of the A- and N-domains.

We have added a panel to Extended Data Fig. 8b to clarify the relative locations of these key catalytic elements, and have added a statement to the text on page 15 of the revised manuscript that explains the underlying rationale.

Several structural changes are noted, such as the tilted cytoplasmic cap and bent membranes, but their structural or functional implications (observed or predicted) are not discussed. For example, does the tilted cytoplasmic cap seem to alter access to the cavity? Since EMC has been proposed to catalyze membrane protein insertion by inducing membrane thinning, could the observed membrane bending have a related effect? Do the authors observe membrane thinning around EMC in their structures?

We have expanded our text to discuss these findings and their potential functional implications.

The tilted cytoplasmic cap likely modulates access to the insertase cavity by altering the geometry of the cytosolic entry region, potentially contributing to regulation of substrate engagement as we now mention on pages 6 and 19 of the revised manuscript.

Consistent with previous reports proposing that the EMC facilitates membrane protein insertion by locally deforming the bilayer, we observe clear differences in detergent micelle thickness around the EMC. Specifically, micelle thickness is reduced on the side containing the EMC1 transmembrane helix and increased on the opposite side harboring the EMC4 gate. This asymmetry propagates through the composite cavity, suggesting that EMC induces a locally distorted, thinned membrane environment that could lower the energetic barrier for insertion. In contrast, we do not observe comparable changes in micelle thickness around Spf1. We have also expanded the discussion to explicitly relate the observed membrane bending and micelle thickness asymmetry to proposed models of EMC-mediated membrane thinning and insertion.

Discussion:

Since EMC is known to interact with multiple other factors, do the authors view the EMC–Spf1 interaction as transient or constitutive? How might EMC accommodate interactions with several different partners?

We see the interaction with Spf1 in yeast as constitutive given that it co-purifies easily at almost 1:1 stoichiometry. Different interactions are mediated by different interfaces (Extended Data Fig. 9b), which may allow simultaneous interaction of the EMC with different partners. These points are now more clearly mentioned in the discussion on page 20.

Are there similarities or differences compared to Msp1 that would be informative to discuss?

Although this is a very interesting idea, we did not identify any points that would be of particular relevance for the Spf1:EMC supercomplex. For Spf1 itself, a potential functional complementation of Spf1 and Msp1 has been discussed in the literature (Dederer & Lemberg, 2021; McKenna & Shao, 2023; McKenna et al., 2020; Sinning & McDowell, 2022).

As noted above, some structural observations (e.g., cytoplasmic cap conformation, membrane bending) are described but not discussed in terms of potential functional implications. Expanding on these points would strengthen the discussion.

Based on these suggestions we have extended the discussion on these points on page 19 as also outlined above.

We hypothesize that the different conformation of the cytoplasmic cap leads to an increased accessibility of the EMC insertase cavity from the cytoplasm, which is then occluded by Spf1's arm in the E1-ATP state.

Membrane deformation may result in multiple biophysical effects such as local distortions, a distinct, less-stiff lipid composition and altered thickness of the lipid bilayer. These effects can facilitate TMD insertion or extraction by lowering the energetic barrier for this process.

Is anything known in the literature about the effects of EMC7/10 deletion? For instance, does it lead to reduced quality control, which would support the authors' findings and their hypothesis that this may result from diminished Spf1 interaction?

Several studies indeed have examined the consequences of Emc7 and Emc10 loss, providing insight into their physiological roles in EMC function and quality control. We have thus extended our description of Emc7 and 10 as accessory EMC subunits on page 8 of the revised manuscript and have added reference to a study that associates prolonged ER retention and delayed export of certain clients with Emc7 KO: (Luo et al., 2002) demonstrated that Emc7 facilitates ER export of specific membrane proteins, including the plasma membrane H^+-ATPase Pma1 and the GPCR Ste2. In Emc7 deletion strains, both wild-type and defective forms of these clients exhibit prolonged residence times in the ER, suggesting altered quality control or delayed

export that may permit additional folding prior to trafficking. Whether these effects are due to diminished Spf1 interaction can not be determined at this stage, but they may provide a basis for further studies.

What is known about the triggers for ATP hydrolysis by Spf1, which would shift the complex from a gated to an ungated form? Conversely, what drives Spf1 to bind ATP and block the gate?

These are excellent questions which, to our knowledge, cannot be fully answered based on the current literature, particularly with respect to the rather recent discovery of Spf1's substrate class. We have added the current state of knowledge and future questions in this regard to the Discussion on pages 19 and 20.

Earlier biochemical studies analyzed the effects of ions and lipids on ATP turnover by purified Spf1(Sorensen et al., 2019), showing that apart from Mg^{2+}, PI4P and also ergosterol and ceramide seem to stimulate the ATPase activity, which is interesting taking into account that we (and others) observed putative lipid binding to Spf1. Further in vitro studies showed that purified Spf1 accumulated in the E1P state rather the E2P state, rather unusual for P-type ATPases, speculating that a trigger for state transition was absent in these assays (Corradi et al., 2012). A recent structural study on the Spf1 homolog from *Caetomium thermophiles* (Li et al., 2024) revealed more details on a putative mechanism for substrate translocation and different intermediate states thereof. Despite these studies, to our knowledge it is not well defined what could trigger ATP hydrolysis and in general it is believed that P-type ATPases do not possess dedicated nucleotide exchange factors rather that ATP binding is controlled by intracellular ATP concentrations and diffusion. Even more so, it remains unclear what the triggers in the EMC:Spf1 supercomplex would be. Our work now lays the basis to address these, as we also think very exciting questions in the future. We have added a statement to the discussion of the revised manuscript on page 19 to account for the present knowledge and open questions on the catalytic cycle of Spf1.

Figures:

Figure1A & Figure 2A --> in the schematic, please label EMC, Spf1, the tag, the antibody

Figure 1C --> please label the peaks on the chromatogram and indicate the corresponding lanes on the SDS-PAGE

The respective labels were added to the figures.

We also added the following references to the manuscript to address the suggestions from reviewers:

Ando, A., & Suzuki, C. (2005). Cooperative function of the CHD5-like protein Mdm39p with a P-type ATPase Spf1p in the maintenance of ER homeostasis in Saccharomyces cerevisiae. *Mol Genet Genomics*, *273*(6), 497-506. https://doi.org/10.1007/s00438-005-1153-6

Cohen, Y., Megyeri, M., Chen, O. C., Condomitti, G., Riezman, I., Loizides-Mangold, U., Abdul-Sada, A., Rimon, N., Riezman, H., Platt, F. M., Futerman, A. H., & Schuldiner, M. (2013). The yeast p5 type ATPase, spf1, regulates manganese transport into the endoplasmic reticulum. *PLoS One*, *8*(12), e85519. https://doi.org/10.1371/journal.pone.0085519

Corradi, G. R., de Tezanos Pinto, F., Mazzitelli, L. R., & Adamo, H. P. (2012). Shadows of an absent partner: ATP hydrolysis and phosphoenzyme turnover of the Spf1 (sensitivity to Pichia farinosa killer toxin) P5-ATPase. *J Biol Chem*, *287*(36), 30477-30484. https://doi.org/10.1074/jbc.M112.363465

Dederer, V., Khmelinskii, A., Huhn, A. G., Okreglak, V., Knop, M., & Lemberg, M. K. (2019). Cooperation of mitochondrial and ER factors in quality control of tail-anchored proteins. *Elife*, *8*. https://doi.org/10.7554/eLife.45506

Dederer, V., & Lemberg, M. K. (2021). Transmembrane dislocases: a second chance for protein targeting. *Trends Cell Biol*, *31*(11), 898-911. https://doi.org/10.1016/j.tcb.2021.05.007

Krumpe, K., Frumkin, I., Herzig, Y., Rimon, N., Ozbalci, C., Brugger, B., Rapaport, D., & Schuldiner, M. (2012). Ergosterol content specifies targeting of tail-anchored proteins to mitochondrial outer membranes. *Mol Biol Cell*, *23*(20), 3927-3935. https://doi.org/10.1091/mbc.E11-12-0994

Li, P., Bagenholm, V., Hagglund, P., Lindkvist-Petersson, K., Wang, K., & Gourdon, P. (2024). The structure and function of P5A-ATPases. *Nat Commun*, *15*(1), 9605. https://doi.org/10.1038/s41467-024-53757-6

Luo, W. J., Gong, X. H., & Chang, A. (2002). An ER membrane protein, Sop4, facilitates ER export of the yeast plasma membrane [H+]ATPase, Pma1. *Traffic*, *3*(10), 730-739. https://doi.org/10.1034/j.1600-0854.2002.31005.x

McKenna, M. J., & Shao, S. (2023). The Endoplasmic Reticulum and the Fidelity of Nascent Protein Localization. *Cold Spring Harb Perspect Biol*, *15*(3). https://doi.org/10.1101/cshperspect.a041249

McKenna, M. J., Sim, S. I., Ordureau, A., Wei, L., Harper, J. W., Shao, S., & Park, E. (2020). The endoplasmic reticulum P5A-ATPase is a transmembrane helix dislocase. *Science*, *369*(6511). https://doi.org/10.1126/science.abc5809

Sinning, I., & McDowell, M. A. (2022). Cryo-EM insights into tail-anchored membrane protein biogenesis in eukaryotes. *Curr Opin Struct Biol*, *75*, 102428. https://doi.org/10.1016/j.sbi.2022.102428

Sorensen, D. M., Holen, H. W., Pedersen, J. T., Martens, H. J., Silvestro, D., Stanchev, L. D., Costa, S. R., Gunther Pomorski, T., Lopez-Marques, R. L., & Palmgren, M. (2019). The P5A ATPase Spf1p is stimulated by phosphatidylinositol 4-phosphate and influences cellular sterol homeostasis. *Mol Biol Cell*, *30*(9), 1069-1084. https://doi.org/10.1091/mbc.E18-06-0365

Response to the Reviewers

We thank all Reviewers for their careful re-evaluation of our manuscript and for acknowledging the additional analysis and experimental work we have performed during the revision to improve our manuscript.

Reviewer #1 (Remarks to the Author):

The authors have addressed my previous comments and the manuscript is ready for publication.

We are delighted that the reviewer appreciates our study and recommends publication in NSMB.

Reviewer #2 (Remarks to the Author):

In the initial review, this reviewer expressed concerns about the physiological significance of the observed EMC:Spf1 supercomplex. In response, the authors performed additional experiments testing the effects of EMC:Spf1 interface disruption on Spf1's functions. The results show that the EMC-interaction mutant (luminal dock mutant E1087A) of Spf1 is fully functional for the dislocation of well-studied TA client proteins Gem1 and Pex10Δ30, comparable to WT Spf1. The authors also found that the mutants stabilize two additional clients, Cst26 and Om14, similarly to WT Spf1. Together, these results indicate that the EMC:Spf1 interaction is not necessary for Spf1's functions on these clients.

The authors also extended their functional analysis to the stability of Erg9 and Gnp1. In the Erg9 assay, the E1087A mutant behaved like WT Spf1. Interestingly, the arm mutant (R866E/W866E/N868D/Q869E) exhibited a hypermorphic phenotype (performing better than WT Spf1), the mechanism of which remains unclear. In the Gnp1 assay, both Spf1 mutants were also largely active like WT, though mild defects were observed under stress conditions (+DTT or terbinafine).

While this reviewer greatly appreciates the authors' efforts in these revision experiments, the overall functional effects of EMC:Spf1 disruption appear weak. It remains unknown how stable this supercomplex is in native membranes, and the current evidence suggests that EMC and Spf1 can each perform their known functions well independently. While the observed structures are interesting, the physiological significance of the supercomplex appears limited.

To address the remaining concerns, we have performed additional experiments and revised the main and SI parts of our manuscript (major changes in the text are highlighted in yellow in the revised version):

"It remains unknown how stable this supercomplex is in native membranes"

We apologize for lack of clarity in the previous versions of our manuscript, but we are very clear now in our further revision: in their pioneering studies defining EMC functions, Jonathan Weissman's laboratory identified Spf1 in mass spectrometric analyses as a stoichiometric partner with the yeast EMC complex (https://pubmed.ncbi.nlm.nih.gov/29809151/). In agreement with and extending this observation, our manuscript presents extensive co-immunoprecipitation data as well as a cryo-EM reconstruction of the **endogenous** EMC:Spf1 complex purified directly from native membranes (Fig. 1, Extended Data Fig. 1, Extended Data Fig. 3). We point out that very few studies of recombinant complexes go to the lengths of

purifying endogenous complexes to validate the assembly. In the attached revision, to further address this point, we have additionally included new data validating that the complex is stable in a membrane (and not only detergent) environment, specifically its reconstitution in lipid nanodiscs (SI Fig. 4). Taken together, published studies and our data provide strong evidence for a stable physiological complex.

"the current evidence suggests that EMC and Spf1 can each perform their known functions well independently"

We apologize for the lack of clarity in our previous text, because we believe this observation actually strengthens the conclusions of our study rather than weakening them. These experiments – which we performed in abundance during the first revision – very clearly address the original concern raised by Reviewer 2 that Spf1 could be an EMC substrate rather than a functional interaction partner. Our functional data clearly rule this out and support our structural data showing that Spf1 can adopt its functionally relevant conformations in complex with EMC. However, retrospectively, we now see that the extensive associated text may have inadvertently fueled concerns about the validity of the EMC:Spf1 complex. We have addressed this in the further revision by shortening and focusing on the main point: Spf1 is as functional in complex with EMC as it is alone.

"the overall functional effects of EMC:Spf1 disruption appear weak"

We also regret that the prior version of the manuscript did not clearly describe the known biological roles of EMC, and mutational effects we observed for EMC clients. Membrane protein insertion and surveillance are such central processes in cell biology that multiple ER membrane protein quality control machineries operate in parallel and can compensate for one another. Accordingly, perturbations of individual pathways often produce only limited phenotypes. For example, classic work from the Hampton laboratory demonstrated that cells rapidly adapt to the loss of ER quality control factors, masking their importance (https://pubmed.ncbi.nlm.nih.gov/29351849/). Similarly, recent studies from the Schuldiner laboratory showed that knockout approaches can fail to accurately reveal endogenous client ranges due to redundancy between insertion pathways (https://pubmed.ncbi.nlm.nih.gov/37073826/), and work from the Hegde and Keenan laboratories demonstrated that even complete loss of multipass translocon factors results in relatively mild phenotypes in cells (Figure 4 of https://pubmed.ncbi.nlm.nih.gov/36261522/). Indeed, our own proteomic analyses of EMC and Spf1 deletion strains, i.e.: full loss of either factor, reveal only relatively limited global proteome changes compared to wild type cells. We include these additional new data in the attached revision in SI Figure 1c, further supporting the well-recognized concept that membrane proteostasis networks are buffered by overlapping pathways and compensatory mechanisms.

The relatively modest phenotypes observed upon disruption of the complex are fully consistent with the well-established robustness and redundancy of ER membrane quality control. The mutations analyzed in our study weaken the interaction by approximately 70% but do not fully abolish it. Nevertheless, the mutants display clear stress-dependent defects, indicating that the interaction contributes measurably to membrane proteostasis under challenging conditions. Importantly, the observation that both machineries retain their core activities is consistent with the conclusion that EMC and Spf1 function as independent systems that can interact without compromising their catalytic cycles.

A highly important emerging topic in the field is that membrane protein biogenesis and quality control machineries can organize into functional hubs within the ER membrane, which brings

multiple machineries into proximity while preserving their independent activities. (https://pubmed.ncbi.nlm.nih.gov/36261528/, https://pubmed.ncbi.nlm.nih.gov/36261522/, https://pubmed.ncbi.nlm.nih.gov/39173640/). The structural basis for forming such assemblies while preserving the activities of the individual constituent machines had been a major unanswered question in the field. Our work provides some of the first structural insights into this principle. The EMC:Spf1 complex illustrates how two major membrane proteostasis machineries can assemble into a higher-order complex while maintaining their individual activities. Rather than forming a joint catalytic machine, this assembly may represent a membrane proteostasis hub that spatially couples insertion, extraction, and chaperoning activities. Such spatial organization will increase the efficiency and fidelity of membrane protein quality control by enabling newly inserted or misassembled membrane proteins to be rapidly assessed and, if necessary, removed locally without requiring diffusion to distant sites in the ER membrane. This notion is fully consistent with the effects we observed under stress, in which the integrative setup becomes even more relevant. In the revision, we now have placed stronger emphasis on this conceptual advance our study provides.

Specific points:

1. The abstract must state that disruption of the EMC:Spf1 supercomplex does not cause functional defects.
We revised the abstract, which now explicitly states that both proteins retain their function in the complex.

2. Given the speculative nature of the current model, Figure 6 should be removed. Alternatively, the figure could be balanced by adding a panel depicting the canonical model of EMC and Spf1 performing their respective functions independently, and moved to the Extended Data Figures.
Based on the Reviewer's suggestions, we have revised Figure 6 and now also depict the functions of the EMC and Spf1 when not in complex with each other and how they are retained in the EMC:Spf1 supercomplex. We further discuss alternative mechanisms in the figure legend. The title of this figure clearly states that this is a "possible model" and we therefore hope that this figure is not misleading but provides a visualization of our major structural findings in the context of a plausible hypothesis.

3. Fig. 5e and f: The authors should confirm by western blotting that Spf1 protein levels in yeast are comparable across mutants and under tested conditions.
As requested, the respective protein levels were assessed by western blot and one representative replicate is now shown in SI Figure 12b.

Reviewer #3 (Remarks to the Author):

The authors have appropriately addressed the reviewer's comments and concerns. The revisions have improved the manuscript, particularly in terms of clarity, figure presentation, and the discussion of structural and mechanistic interpretations. The additional explanations regarding the composite cavity, nucleotide states, and the functional implications of the observed conformational changes are especially helpful. I appreciate the authors' careful and thoughtful responses, and I believe the manuscript is now suitable for publication.

We are delighted that the reviewer appreciates our study and recommends publication in NSMB.

Reply to the reviewers' comments:

Reviewer #1 (Remarks to the Author):

The manuscript by Klose et al. presents a break-through structural study of the yeast EMC in a previously unanticipated complex with the Spf1 P5A-ATPase. Using a combination of co-immunoprecipitation (co-IP) and mass spectrometry, the authors discover that EMC/Spf1 forms a stable complex at endogenous levels, and that the analogous EMC/ATP13A1 complex is conserved in human cells. They then solve a cryo-EM structure of the recombinant EMC/Spf1 complex at high-resolution, and verify that a low-resolution structure of the endogenous yeast complex has the same organisation. This apo structure reveals a juxtaposition of the EMC insertase and Spf1 dislocase sites, a pronounced kink in the putative membrane plane and an interaction interface formed by the luminal domains, the importance of which is verified by co-IPs of mutagenised subunits. An AlphaFold3 model for the EMC/ATP13A1 complex suggests a similar mode of interaction, which is characterised further by the same co-IP approach. Lastly, the authors solve cryo-EM structures of the EMC/Spf1 complex with ATPgS and AlF4- to provide models for the E1-ATP and E1-P state of Spf1 respectively. They obtain additional density for the Spf1 arm in the E1-ATP state, which provides an additional stabilising interface with the cytosolic domains of the EMC. Overall, the results are of high interest, providing pioneering evidence that TMD insertase and dislocase activity can be combined into single supercomplex. The experimental data are compelling and the manuscript is well-presented, so my enthusiasm for publication is high, pending a few points to be addressed

We are delighted by the reviewer's interest for our study. We thank the reviewer for the helpful suggestions. Addressing the points raised has helped to further improve our manuscript.

1. The quality of the cryo-EM maps is high (and I appreciate the ChimeraX session for reviewers), nevertheless I think there should be a figure showing snapshots of model fit to density, particularly for the relevant luminal dock and Spf1 arm interfaces, and regions that are resolved additionally or significantly better than in previous structures.

We have added images of model fit to density for key structural regions (SI Figure 4).

2. Page 6: "the cytoplasmic cap…was tilted and displaced" - it's not clear whether the authors think this is due to the Spf1 (arm) interaction?

In the revised manuscript, we have adjusted the wording to better describe the observed changes on pages 6 and 19. Because this effect was also seen in the apo structure that did not resolve the arm, we do not think that this is only due to the newly formed interface. However, it is striking that this difference is consistent for the comparisons across all three published structures of the yeast EMC without Spf1. We would like to remain cautious about an interpretation for this structural observation, and thus we simply note that the entrance to the insertase cavity from the cytosol is relatively more accessible in the structures with Spf1. The same conformation of the EMC cap can also be observed in the ATPγS bound state (with Spf1), yet the arm is now blocking access to the cavity from the cytosol in a lid-like manner.

3. Page 6: “and it is also implicated in the insertion of TMDs separated by only short hydrophilic loops.” I would remove this statement as there is no experimental evidence, rather a suggestion from the cited review based on the absence of the GEL complex in yeast.

We thank the reviewer for pointing this out and have removed this statement from the text.

4. From the panels in Fig. 2 and Extended Data Fig. 4, it is hard to see that the “functional sites for substrate engagement directly face each other”. I would suggest to show a panel where the substrate binding sites are highlighted on the model/map in the same colour.

To address this, our revised manuscript now highlights this structural feature in Figure 2d and Extended Data Fig. 4d, 4e.

5. Even in the apo map, it seems like the Emc4 gate is better resolved than in previous structures, suggesting it is stabilised in the observed conformation. The position in the E1-ATP map is not much different despite having better resolved cytosolic features. Is the Emc3 hydrophilic groove accessible for substrate insertion in this conformation? Do the authors still envisage the gate is dynamic in the context of the complex with Spf1? It would be worth to add these points to the text.

We agree with the reviewer that even in the apo reconstruction, the Emc4 gate is better resolved than in previously reported EMC structures, suggesting stabilization of a defined conformation. Of note, to date, multiple distinct gate conformations were reported for the human EMC; only a single gate conformation was observed in all the three prior structures of the yeast complex. The position of the Emc4 gate in the E1–ATP map is also very similar to that in the apo map, despite improved resolution of cytosolic features. In this conformation, access to the Emc3 hydrophilic groove is restricted, as entry is occluded by the arm domain. Thus, any notions of gate flexibility in yeast are speculative, and we have adapted the discussion to account for this. As we now discuss on page 19 of the revised manuscript, this gate arrangement laterally delimits the composite cavity.

6. On page 10: although the authors say “the structural prediction suggests an extended intramembrane interface”, they do not directly comment on the more planar orientation of the modelled human complex compared to the yeast complex. What I assume is the ATP13A1 arm is also quite prominent in the AlphaFold3 model shown in Fig. 4b and Extended Fig. 6a. Does it have a sensible conformation/interface with EMC? Would you expect the arm/EMC interaction to be conserved in humans and would the human complex have to orient differently to form this interaction?

The Spf1/ATP13A1 arm domain is poorly conserved between different species (Li et al., 2024), and the sequence of the human arm is much shorter (by 52 residues) than that from yeast. So, if the same arm-EMC interface exists, this might explain why the orientation is not angled/tilted by the interactions mediated by the arm. However, AlphaFold3 predicts the same complex orientation if the human structure is predicted with or without the arm domain (data not shown), suggesting that the EMC-arm

interface does not determine the supercomplex architecture in humans. Furthermore, the nature of the human ATP13A1:EMC interaction is different to the yeast Spf1:EMC one (e.g. missing the lumenal dock, as our data show and as we describe in the manuscript), which may also explain the lack of membrane curvature in the human complex. We now more explicitly mention this on page 12 of the revised manuscript.

7. Extended Data Fig. 7: what are the cross-linked species formed between EMC10 wt and EMC7 M168C or P172C? They appear at the same molecular weight as the cross-link with EMC10 Y230C and L238C – does this not suggest cross-linking with the naturally occurring cysteines?

We think it is most likely that EMC7 crosslinks with another partner, because the crosslinked bands are not recognized by the antibody recognizing the FLAG-tag on the expressed EMC10 (although we cannot exclude that the crosslinks do exclusively form with endogenous EMC10 and not the transfected variant, but this seems unlikely). The bands could for example indicate crosslinking with another EMC subunit such as EMC4, in which case the IP based on the tagged EMC subunit (EMC10) would still isolate this complex. We added this information to the figure legend of Extended Data Fig. 7.

8. Page 10: "we observed substantial conformational heterogeneity of Spf1 in our map of the endogenous EMC:Spf1 complex". Was this heterogeneity not observed for the recombinant complex? Are the complexes bound to the nucleotide analogues more homogeneous? The processing strategies and pipelines are quite different for the various structures and could lead to different conclusions about heterogeneity; what was the rationale for this?

We fully agree that this was misleading and removed the statement from the revised manuscript.

9. On a similar note, how do the two conformations shown in Fig. 5a compare to the apo complex? Does this complex essentially have the same conformation as the E1-P state or are there differences?

To compare these different states, we low pass filtered all maps to a resolution of 10 Å, allowing more accurate assessment of domain positioning that is not biased by differences in resolution. From this we conclude that the changes observed in Spf1 positioning and size of the composite cavity are most pronounced when comparing the ATPγS- and AlF_4^--bound states. In the last consensus map of the apo state, before separate refinement of EMC and Spf1 (see SI Fig. 2 for data processing details), Spf1 occupies a position broadly similar to that observed in the E1-ATP state, albeit slightly closer to the EMC.

In our speculative model for an ATP-driven translocation cycle (Fig. 6), this apo state would correspond to the E1 conformation, where we propose client helix release into the membrane or cytoplasm. Hence, an increased distance between the EMC and Spf1 might facilitate such a release process.

We have added this new comparison to Extended Data Fig. 8c and added a respective statement on page 15.

10. Page 10: currently there is no mention of whether the nucleotide analogues are resolved in the maps, the position of their binding site with respect to the Spf1 arm and how changes at the nucleotide binding site might be correlated with the stabilisation of the arm domain. These points would make a valuable addition to the text.

In the ATPγS-bound state, we observe additional density in the nucleotide binding site clearly indicating the presence of a nucleotide in the unsharpened cryo-EM map. However, the resolution is not sufficient to precisely build this nucleotide analog in the structural model. The nucleotide analog was added in approximately 200-fold molar excess to the protein complex and we see a clear conformational change in comparison to the apo state, now resembling the published E1-ATP conformation in the presence of AMPPCP (PDB: 6XMQ) (McKenna et al., 2020). These features led us to surmise that this map represents the E1-ATP state. We have now added this to the Methods section. We also mention this clearly in the Results section on page 15, and show the density in the nucleotide-binding site in Extended Data Fig. 8a.

Similarly, the cryo-EM map acquired in the presence of AlF_4^- agrees with the previously reported structure of Spf1 reconsituted with the same nucleotide analog that defined the E1-P state (PDB: 6XMS) (McKenna et al., 2020). The resolution of our map does not allow to determine the presence or absence of a nucleotide. Therefore, we are cautious in the text about the nucleotide state of this map. We have added an additional statement to clarify this on page 15 of the revised manuscript.

Structurally, the arm domain is positioned in close proximity to key catalytic elements that undergo large rearrangements upon ATP binding and hydrolysis. Notably, the catalytic aspartate in the P-domain is located ~15 Å away from the N- and C-termini of the arm domain, and the conserved SGE dephosphorylation motif in the A-domain is positioned between these termini. Given that ATP binding and hydrolysis are known to drive substantial relative movements of the A- and N-domains in P-type ATPases, these rearrangements provide a plausible structural basis for an indirect, relay-like coupling to the arm domain, thereby driving its observed repositioning in the ATPγS-bound state. So while our data do not allow us to define the precise molecular pathway of this coupling, the spatial arrangement of the arm relative to the catalytic core supports a model where nucleotide-dependent domain motions are transmitted to the arm domain through coordinated movements of the A- and N-domains.

We have added a panel to Extended Data Fig. 8b to clarify the relative locations of these key catalytic elements, and have added a statement to the text on page 15 of the revised manuscript that explains the underlying rationale.

Reviewer #2 (Remarks to the Author):

In this manuscript, Klose et al. report a cryo-EM structure of the yeast EMC:Spf1 complex. The EMC is a conserved ER-membrane complex that mediates the insertion of certain transmembrane helices, particularly those located near the N- and C-termini. Spf1 (ATP13A1in mammals) is a P-type ATPase that has been shown to function as a dislocase for mis-inserted transmembrane helices at the ER. In this sense, EMC and Spf1 act in the same general pathway but in opposite directions. The authors present a structure suggesting direct complex formation between the two, raising the interesting possibility that Spf1 serves as a proofreading factor for EMC-mediated membrane insertion. Using AlphaFold modeling, the authors further propose that the human EMC and ATP13A1 may form a similar complex although the interaction surfaces are not conserved. Mutagenesis analysis indicates that mutations in these sites reduce co-immunoprecipitation efficiency in both yeast and human complexes. While the model is interesting and, in principle, plausible, its physiological significance remains uncertain. The manuscript lacks functional experiments beyond binding assays, leaving the proposed mechanism largely speculative. Recent reports of EMC interactions with BOS (ref. 12), CaV (ref. 17), and VDAC (ref. 55)—the latter likely an artifact given VDAC's mitochondrial localization, raise concerns about the "stickiness" of EMC, whether this may reflect a chaperone-like role or experimental artifact from the use of detergent. In this regard, Spf1 could simply represent another EMC client, rather than a proofreading factor. Without addressing these issues, the model remains speculative, and new insights are limited.

We are very pleased that the reviewer found our structural data and model interesting. We thank the reviewer for the helpful suggestions. Addressing the points has helped to further improve our manuscript.

1. Biological significance of EMC:Spf1 formation: Do interaction-disrupting mutants affect the function of Spf1? There are several assays established for Spf1, for example, mistargeting mitochondrial proteins (e.g., Pex15Δ30 and Gem1) to the ER. Do the mutants fail to rescue mis-localization phenotypes?

This indeed is a highly relevant point. To address this, we performed many new experiments, now shown in Fig. 3d, 5e, 5f and Extended Data Fig. 5e, 5f, 5g, 8d, 8e and mentioned in the results of our revised manuscript.

As suggested by the reviewer, we performed several established assays for Spf1 function: 1. sensitivity to caffeine in a growth assay (Ando & Suzuki, 2005) (Extended Data Fig. 5e) 2. mislocalization of the endogenous mitochondrial TA protein Gem1 as seen by fluorescence microscopy (Krumpe et al., 2012) (Extended Data Fig. 5g) and 3. stabilization of a Pex15Δ30 stability reporter in flow cytometry (Dederer et al., 2019) (Fig. 3d, Extended Data Fig 5f). In addition, we designed further protein stability reporters allowing flow cytometry based readouts for the Spf1 client Cst26 (Cohen et al., 2013) and the Spf1 and EMC interactor Om14 (Fig. 3d, Extended Data Fig. 5f).

To be able to test roles of Spf1 and EMC, and to examine effects of mutations, we generated ΔSpf1 and ΔEmc3 yeast strains, as well as a control strain where the same antibiotic resistance was integrated into the HO locus. These were then transformed

with a centromeric plasmid encoding for an Spf1 variant or an empty vector and then subjected to the respective assay.

All the abovementioned assays were insensitive to an Emc3 KO, indicating that they truly report only on Spf1 activity. Our Spf1 mutants that disrupt the interaction with the EMC (E1087A at the lumenal dock and R866E W867E N868D Q869E designated as "arm mutation") showed a similar behavior to the strain with wildtype Spf1 in all of these Spf1 activity assays, in contrast to controls that eliminate Spf1 catalytic activity (D487A) or the unrescued Spf1 KO. This indicates that interaction with the EMC is not required for these functions of Spf1 and that an EMC-interaction-deficient mutant is still catalytically active. Furthermore, we conclude that the biogenesis of Spf1 is still successful when disrupting the interaction with the EMC and thus Spf1 is not dependant on the EMC for maturation as a client via our observed interactions.

Additionally, we assessed the effects of Spf1 mutations on two EMC client proteins in the flow cytometry-based protein stability assay: Erg9, the human ortholog of squalene synthase (SQS/FDFT1) and Gnp1, a multipass protein that was shown to be destabilized in the absence of the EMC. We now include this data in Fig. 5e, f and Extended Data Fig. 8d, e.

Interestingly, Erg9 is destabilized when the EMC:arm interface is mutated and stabilized in the context of inactive Spf1 (D487A) or an unrescued Spf1 KO. This data could suggest that Spf1 plays a role in dislocating Erg9 from the ER membrane and that this dislocation mechanism is misregulated when the EMC:arm interface is disrupted.

In contrast, there is a slight (5%) increase in Gnp1 stability in the strain with the arm mutation, indicating that this mutant cannot fully rescue the Spf1 KO phenotype. This effect is more pronounced when subjecting the cells to the stress of DTT. The lumenal dock mutation (E1087A) shows a similar stabilizing effect when ergosterol biosynthesis is inhibited by Terbinafine.

These data provide first insights into functional consequences of disrupting the EMC:Spf1 supercomplex for certain EMC/Spf1 co-clients but are far from a comprehensive or mechanistic understanding. We envision protein targeting and insertion as a dynamic process that is constantly surveilled and corrected by a large network of quality control factors. While the current picture of this network is far from complete, we speculate that manipulation of one player may result in a spectrum of different outcomes, depending on compensatory mechanisms. We attribute the opposite phenotypes observed with Erg9 and Gnp1 upon Spf1 KO or disruption of the EMC:Spf1 interaction to this unexplored complexity of the network and a not fully understood role of Spf1 and the EMC in the biogenesis of their clients. This is now also mentioned in the Results section as well as in the Discussion.

2. Did the authors test AlphaFold predictions for the yeast complex? Does it produce consistent results that agree with the cryo-EM structure?

As suggested, we now add an AlphaFold3 model of the yeast complex to SI Fig. 6 and mention this in the revised results section. The model is overall consistent with our experimental structure, predicting the same overall architecture of the supercomplex

as well as the lumenal dock as the major interaction interface. We note differences in the angular orientation of Spf1 towards the EMC and variability in the arm conformations (which we find to be nucleotide-regulated).

3. AlphaFold was used to generate a model for the human EMC:ATP13A1 complex. The authors should show the PAE matrix and report structural variability across multiple predicted models, particularly in the interaction regions.

The PAE matrix, model confidence, and structural variability are shown in SI Figure 5 of the revised manuscript.

Minor point:
This term “shared cavity” is misleading. EMC and Spf1 each have distinct cavities. In the complex, there is a sizeable gap between the two cavities, across which the substrate may diffuse.

We agree and thus have renamed this “composite intramembrane cavity” throughout the text.

Reviewer #3 (Remarks to the Author):

Summary:

In this study, the authors demonstrate that the EMC and Spf1/ATP13A1 form a stable, evolutionarily conserved supercomplex in the eukaryotic ER that physically links membrane protein insertase and dislocase activities. Their cryo-EM structures show EMC and Spf1 form a composite intramembrane cavity, and the authors suggest that this cavity enables coordinated substrate engagement, topogenesis, and error correction. They also suggest that ATP-driven conformational changes of Spf1 control cavity access for protein insertion or extraction. Key interactions between the two proteins are mediated by accessory subunits Emc7 and Emc10, confirmed by mutagenesis, with structural conservation across yeast and human validated by modeling using AlphaFold3. Together, the findings establish a new paradigm for membrane protein quality control in eukaryotes, integrating opposing activities (protein insertion and removal) through a unified molecular machine.

General Comments:
This is a well-written and clearly presented study supported by strong experimental evidence. The work addresses an important and interesting question regarding the interaction between the insertase EMC and the dislocase Spf1. The structure determination of this complex in multiple conformations is a significant and exciting achievement, as it provides new insights such as the identification of shared substrate-binding cavities between the two proteins and the observation of nucleotide-dependent conformational changes.
That said, some parts of the manuscript require clearer explanation and improved figures, especially the description of the shared cavity, which represents one of the major findings in this study. The introduction and discussion could also be expanded to better situate the authors' findings within the broader context.
Below are specific comments organized in their order of appearance in the manuscript.

We are delighted by the interest in our manuscript and thank the reviewer for the helpful suggestions. We have addressed all remaining concerns as outlined below.

Introduction:

Introduction is generally well written, but the discussion of what is known about EMC and Spf1 is rather brief. A concise overview of the current state of knowledge from prior structural and biochemical studies would strengthen the introduction (without going into lots of detail, which is always a challenge, of course). Although some of this background is mentioned later in the results and discussion, presenting it here would help readers better appreciate the context of the work. For example, a brief description of EMC's subunit composition, the roles of individual units, and the current model of its mechanism would be valuable. A similar point applies to Spf1. Summarizing the current understanding of Spf1's substrate recognition and dislocation mechanism would add valuable context.

Picking up this suggestion, while still aiming for a concise introduction, we have extended the background about EMC and Spf1 in the introduction, adding more on their architecture and current model of mechanism on pages 3 and 4.

“EMC…serves as a central hub in membrane protein biogenesis and quality control by interacting with multiple further quality control factors” – Please provide some examples.

In the revised manuscript, we now add some examples and references for these on page 3.

Results:

Across all structures determined, it is not clear whether the nucleotide density is well resolved or whether nucleotides were modeled (the provided models suggest they are not). For example, did the authors observe clear density for ATPγS? If not, it should be explicitly stated, and how they determined that the reconstructed class corresponds to a given nucleotide state rather than another.

In the ATPγS-bound state, we observe density in the nucleotide binding site clearly indicating the presence of a nucleotide in the unsharpened cryo-EM map. However, the resolution is not sufficient to precisely build this nucleotide analog in the structural model. The nucleotide analog was added in approximately 200-fold molar excess to the protein complex and we see a clear conformational change in comparison to the apo state, now resembling the published E1-ATP conformation in the presence of AMPPCP (PDB: 6XMQ) (McKenna et al., 2020). These features led us to surmise that this map represents the E1-ATP state. We have now added this to the Methods section. We also mention this clearly in the Results section on page 15, and show the density in the nucleotide-binding site in Extended Data Fig. 8a.

Similarly, the cryo-EM map acquired in the presence of AlF_4^- agrees with the previously reported structure of Spf1 reconsituted with the same nucleotide analog that defined the E1-P state (PDB: 6XMS) (McKenna et al., 2020). The resolution of our map does not allow to determine the presence or absence of a nucleotide. Therefore, we are cautious in the text about the nucleotide state of this map. We have added an additional statement to clarify this on page 15 of the revised manuscript.

The composite intramembrane cavity shared by EMC and Spf1 needs to be more clearly described both in text and figures. In the figures, the approximate locations of the substrate-binding sites for EMC and Spf1 are indicated, but the shape and properties of these sites are difficult to discern. It may help to represent the full structure in cartoon mode but highlight the substrate-binding sites in surface representation (or another visualization strategy) to make them more apparent. From the provided figures, it is also difficult to see where the positively charged selective filters are located in both proteins. It would also be informative to show an electrostatic map and/or the residues that function as the selective filters.

We have adapted our representation and description of the composite cavity in the revised Fig. 2d and Extended Data Fig. 4d, e, now showing the surface and electrostatic properties of both substrate binding sites more clearly.

The conformational change of the Spf1 arm in the ATPγS-bound state is particularly interesting. Can the authors provide structural insights into the basis of this conformational change, specifically, how ATP binding drives the movement of the arm? In addition, it is not entirely clear from the figures where the nucleotide-binding site is located.

Structurally, the arm domain is positioned in close proximity to key catalytic elements that undergo large rearrangements upon ATP binding and hydrolysis. Notably, the catalytic aspartate in the P-domain is located ~15 Å away from the N- and C-termini of the arm domain, and the conserved SGE dephosphorylation motif in the A-domain is positioned between these termini. Given that ATP binding and hydrolysis are known to drive substantial relative movements of the A- and N-domains in P-type ATPases, these rearrangements provide a plausible structural basis for an indirect, relay-like coupling to the arm domain, thereby driving its observed repositioning in the ATPγS-bound state. So while our data do not allow us to define the precise molecular pathway of this coupling, the spatial arrangement of the arm relative to the catalytic core supports a model where nucleotide-dependent domain motions are transmitted to the arm domain through coordinated movements of the A- and N-domains.

We have added a panel to Extended Data Fig. 8b to clarify the relative locations of these key catalytic elements, and have added a statement to the text on page 15 of the revised manuscript that explains the underlying rationale.

Several structural changes are noted, such as the tilted cytoplasmic cap and bent membranes, but their structural or functional implications (observed or predicted) are not discussed. For example, does the tilted cytoplasmic cap seem to alter access to the cavity? Since EMC has been proposed to catalyze membrane protein insertion by inducing membrane thinning, could the observed membrane bending have a related effect? Do the authors observe membrane thinning around EMC in their structures?

We have expanded our text to discuss these findings and their potential functional implications.

The tilted cytoplasmic cap likely modulates access to the insertase cavity by altering the geometry of the cytosolic entry region, potentially contributing to regulation of substrate engagement as we now mention on pages 6 and 19 of the revised manuscript.

Consistent with previous reports proposing that the EMC facilitates membrane protein insertion by locally deforming the bilayer, we observe clear differences in detergent micelle thickness around the EMC. Specifically, micelle thickness is reduced on the side containing the EMC1 transmembrane helix and increased on the opposite side harboring the EMC4 gate. This asymmetry propagates through the composite cavity, suggesting that EMC induces a locally distorted, thinned membrane environment that could lower the energetic barrier for insertion. In contrast, we do not observe comparable changes in micelle thickness around Spf1. We have also expanded the discussion to explicitly relate the observed membrane bending and micelle thickness asymmetry to proposed models of EMC-mediated membrane thinning and insertion.

Discussion:

Since EMC is known to interact with multiple other factors, do the authors view the EMC–Spf1 interaction as transient or constitutive? How might EMC accommodate interactions with several different partners?

We see the interaction with Spf1 in yeast as constitutive given that it co-purifies easily at almost 1:1 stoichiometry. Different interactions are mediated by different interfaces (Extended Data Fig. 9b), which may allow simultaneous interaction of the EMC with different partners. These points are now more clearly mentioned in the discussion on page 20.

Are there similarities or differences compared to Msp1 that would be informative to discuss?

Although this is a very interesting idea, we did not identify any points that would be of particular relevance for the Spf1:EMC supercomplex. For Spf1 itself, a potential functional complementation of Spf1 and Msp1 has been discussed in the literature (Dederer & Lemberg, 2021; McKenna & Shao, 2023; McKenna et al., 2020; Sinning & McDowell, 2022).

As noted above, some structural observations (e.g., cytoplasmic cap conformation, membrane bending) are described but not discussed in terms of potential functional implications. Expanding on these points would strengthen the discussion.

Based on these suggestions we have extended the discussion on these points on page 19 as also outlined above.

We hypothesize that the different conformation of the cytoplasmic cap leads to an increased accessibility of the EMC insertase cavity from the cytoplasm, which is then occluded by Spf1's arm in the E1-ATP state.

Membrane deformation may result in multiple biophysical effects such as local distortions, a distinct, less-stiff lipid composition and altered thickness of the lipid bilayer. These effects can facilitate TMD insertion or extraction by lowering the energetic barrier for this process.

Is anything known in the literature about the effects of EMC7/10 deletion? For instance, does it lead to reduced quality control, which would support the authors' findings and their hypothesis that this may result from diminished Spf1 interaction?

Several studies indeed have examined the consequences of Emc7 and Emc10 loss, providing insight into their physiological roles in EMC function and quality control. We have thus extended our description of Emc7 and 10 as accessory EMC subunits on page 8 of the revised manuscript and have added reference to a study that associates prolonged ER retention and delayed export of certain clients with Emc7 KO: (Luo et al., 2002) demonstrated that Emc7 facilitates ER export of specific membrane proteins, including the plasma membrane H^+-ATPase Pma1 and the GPCR Ste2. In Emc7 deletion strains, both wild-type and defective forms of these clients exhibit prolonged residence times in the ER, suggesting altered quality control or delayed

export that may permit additional folding prior to trafficking. Whether these effects are due to diminished Spf1 interaction can not be determined at this stage, but they may provide a basis for further studies.

What is known about the triggers for ATP hydrolysis by Spf1, which would shift the complex from a gated to an ungated form? Conversely, what drives Spf1 to bind ATP and block the gate?

These are excellent questions which, to our knowledge, cannot be fully answered based on the current literature, particularly with respect to the rather recent discovery of Spf1's substrate class. We have added the current state of knowledge and future questions in this regard to the Discussion on pages 19 and 20.

Earlier biochemical studies analyzed the effects of ions and lipids on ATP turnover by purified Spf1(Sorensen et al., 2019), showing that apart from Mg^{2+}, PI4P and also ergosterol and ceramide seem to stimulate the ATPase activity, which is interesting taking into account that we (and others) observed putative lipid binding to Spf1. Further in vitro studies showed that purified Spf1 accumulated in the E1P state rather the E2P state, rather unusual for P-type ATPases, speculating that a trigger for state transition was absent in these assays (Corradi et al., 2012). A recent structural study on the Spf1 homolog from *Caetomium thermophiles* (Li et al., 2024) revealed more details on a putative mechanism for substrate translocation and different intermediate states thereof. Despite these studies, to our knowledge it is not well defined what could trigger ATP hydrolysis and in general it is believed that P-type ATPases do not possess dedicated nucleotide exchange factors rather that ATP binding is controlled by intracellular ATP concentrations and diffusion. Even more so, it remains unclear what the triggers in the EMC:Spf1 supercomplex would be. Our work now lays the basis to address these, as we also think very exciting questions in the future. We have added a statement to the discussion of the revised manuscript on page 19 to account for the present knowledge and open questions on the catalytic cycle of Spf1.

Figures:

Figure1A & Figure 2A --> in the schematic, please label EMC, Spf1, the tag, the antibody

Figure 1C --> please label the peaks on the chromatogram and indicate the corresponding lanes on the SDS-PAGE

The respective labels were added to the figures.

We also added the following references to the manuscript to address the suggestions from reviewers:

Ando, A., & Suzuki, C. (2005). Cooperative function of the CHD5-like protein Mdm39p with a P-type ATPase Spf1p in the maintenance of ER homeostasis in Saccharomyces cerevisiae. *Mol Genet Genomics*, *273*(6), 497-506. https://doi.org/10.1007/s00438-005-1153-6

Cohen, Y., Megyeri, M., Chen, O. C., Condomitti, G., Riezman, I., Loizides-Mangold, U., Abdul-Sada, A., Rimon, N., Riezman, H., Platt, F. M., Futerman, A. H., & Schuldiner, M. (2013). The yeast p5 type ATPase, spf1, regulates manganese transport into the endoplasmic reticulum. *PLoS One*, *8*(12), e85519. https://doi.org/10.1371/journal.pone.0085519

Corradi, G. R., de Tezanos Pinto, F., Mazzitelli, L. R., & Adamo, H. P. (2012). Shadows of an absent partner: ATP hydrolysis and phosphoenzyme turnover of the Spf1 (sensitivity to Pichia farinosa killer toxin) P5-ATPase. *J Biol Chem*, *287*(36), 30477-30484. https://doi.org/10.1074/jbc.M112.363465

Dederer, V., Khmelinskii, A., Huhn, A. G., Okreglak, V., Knop, M., & Lemberg, M. K. (2019). Cooperation of mitochondrial and ER factors in quality control of tail-anchored proteins. *Elife*, *8*. https://doi.org/10.7554/eLife.45506

Dederer, V., & Lemberg, M. K. (2021). Transmembrane dislocases: a second chance for protein targeting. *Trends Cell Biol*, *31*(11), 898-911. https://doi.org/10.1016/j.tcb.2021.05.007

Krumpe, K., Frumkin, I., Herzig, Y., Rimon, N., Ozbalci, C., Brugger, B., Rapaport, D., & Schuldiner, M. (2012). Ergosterol content specifies targeting of tail-anchored proteins to mitochondrial outer membranes. *Mol Biol Cell*, *23*(20), 3927-3935. https://doi.org/10.1091/mbc.E11-12-0994

Li, P., Bagenholm, V., Hagglund, P., Lindkvist-Petersson, K., Wang, K., & Gourdon, P. (2024). The structure and function of P5A-ATPases. *Nat Commun*, *15*(1), 9605. https://doi.org/10.1038/s41467-024-53757-6

Luo, W. J., Gong, X. H., & Chang, A. (2002). An ER membrane protein, Sop4, facilitates ER export of the yeast plasma membrane [H+]ATPase, Pma1. *Traffic*, *3*(10), 730-739. https://doi.org/10.1034/j.1600-0854.2002.31005.x

McKenna, M. J., & Shao, S. (2023). The Endoplasmic Reticulum and the Fidelity of Nascent Protein Localization. *Cold Spring Harb Perspect Biol*, *15*(3). https://doi.org/10.1101/cshperspect.a041249

McKenna, M. J., Sim, S. I., Ordureau, A., Wei, L., Harper, J. W., Shao, S., & Park, E. (2020). The endoplasmic reticulum P5A-ATPase is a transmembrane helix dislocase. *Science*, *369*(6511). https://doi.org/10.1126/science.abc5809

Sinning, I., & McDowell, M. A. (2022). Cryo-EM insights into tail-anchored membrane protein biogenesis in eukaryotes. *Curr Opin Struct Biol*, *75*, 102428. https://doi.org/10.1016/j.sbi.2022.102428

Sorensen, D. M., Holen, H. W., Pedersen, J. T., Martens, H. J., Silvestro, D., Stanchev, L. D., Costa, S. R., Gunther Pomorski, T., Lopez-Marques, R. L., & Palmgren, M. (2019). The P5A ATPase Spf1p is stimulated by phosphatidylinositol 4-phosphate and influences cellular sterol homeostasis. *Mol Biol Cell*, *30*(9), 1069-1084. https://doi.org/10.1091/mbc.E18-06-0365

Reply to the reviewers' comments:

We thank all Reviewers for their careful re-evaluation of our manuscript and for acknowledging the additional analysis and experimental work performed during the revision to improve our manuscript.

Reviewer #1 (Remarks to the Author):

The authors have addressed my previous comments and the manuscript is ready for publication.

We are delighted that the reviewer appreciates our study and recommends publication in NSMB.

Reviewer #2 (Remarks to the Author):

In the initial review, this reviewer expressed concerns about the physiological significance of the observed EMC:Spf1 supercomplex. In response, the authors performed additional experiments testing the effects of EMC:Spf1 interface disruption on Spf1's functions. The results show that the EMC-interaction mutant (luminal dock mutant E1087A) of Spf1 is fully functional for the dislocation of well-studied TA client proteins Gem1 and Pex10Δ30, comparable to WT Spf1. The authors also found that the mutants stabilize two additional clients, Cst26 and Om14, similarly to WT Spf1. Together, these results indicate that the EMC:Spf1 interaction is not necessary for Spf1's functions on these clients.

The authors also extended their functional analysis to the stability of Erg9 and Gnp1. In the Erg9 assay, the E1087A mutant behaved like WT Spf1. Interestingly, the arm mutant (R866E/W866E/N868D/Q869E) exhibited a hypermorphic phenotype (performing better than WT Spf1), the mechanism of which remains unclear. In the Gnp1 assay, both Spf1 mutants were also largely active like WT, though mild defects were observed under stress conditions (+DTT or terbinafine).

While this reviewer greatly appreciates the authors' efforts in these revision experiments, the overall functional effects of EMC:Spf1 disruption appear weak. It remains unknown how stable this supercomplex is in native membranes, and the current evidence suggests that EMC and Spf1 can each perform their known functions well independently. While the observed structures are interesting, the physiological significance of the supercomplex appears limited.

To address the remaining concerns, we have performed additional experiments and revised the main and SI parts of our manuscript, as follows:

“It remains unknown how stable this supercomplex is in native membranes”
We apologize for lack of clarity in the previous versions of our manuscript, but we are very clear now in our further revision: in their pioneering studies defining EMC functions, Jonathan Weissman’s laboratory identified Spf1 in mass spectrometric analyses as a stoichiometric partner with the yeast EMC complex (https://pubmed.ncbi.nlm.nih.gov/29809151/). In agreement with and extending this

observation, our manuscript presents extensive co-immunoprecipitation data as well as a cryo-EM reconstruction of the **endogenous** EMC:Spf1 complex purified directly from native membranes (Fig. 1, Extended Data Fig. 1, Extended Data Fig. 3). We point out that very few studies of recombinant complexes go to the lengths of purifying endogenous complexes to validate the assembly. In the attached revision, we have additionally included further data validating that the complex is stable in a membrane (and not only detergent) environment, specifically its reconstitution in lipid nanodiscs (SI Fig. 4). Taken together, published studies and our data provide strong evidence for a stable physiological complex.

“the current evidence suggests that EMC and Spf1 can each perform their known functions well independently”
We apologize for the lack of clarity in our previous text, because we believe this observation actually strengthens the conclusions of our study rather than weakening them. These experiments – which we performed in abundance during the first revision – very clearly address the original concern raised by Reviewer 2 that Spf1 could be an EMC substrate rather than a functional interaction partner. Our functional data clearly rule this out, and support our structural data showing that Spf1 can adopt its functionally relevant conformations in complex with EMC. However, retrospectively, we now see that the extensive associated text may have inadvertently fueled concerns about the validity of the EMC:Spf1 complex. We have addressed this in the further revision by shortening and focusing on the main point: Spf1 is as functional in complex with EMC as it is alone.

“the overall functional effects of EMC:Spf1 disruption appear weak”
We also regret that the prior version of the manuscript did not clearly describe the known biological roles of EMC, and mutational effects we observed for EMC clients. Membrane protein insertion and surveillance are such central processes in cell biology that multiple ER membrane protein quality control machineries operate in parallel and can compensate for one another. Accordingly, perturbations of individual pathways often produce only limited phenotypes. For example, classic work from the Hampton laboratory demonstrated that cells rapidly adapt to the loss of ER quality control factors, masking their importance (https://pubmed.ncbi.nlm.nih.gov/29351849/). Similarly, recent studies from the Schuldiner laboratory showed that knockout approaches can fail to accurately reveal endogenous client ranges due to redundancy between insertion pathways (https://pubmed.ncbi.nlm.nih.gov/37073826/), and work from the Hegde and Keenan laboratories demonstrated that even complete loss of multipass translocon factors results in relatively mild phenotypes in cells (see Figure 4 of https://pubmed.ncbi.nlm.nih.gov/36261522/). Indeed, our own proteomic analyses of EMC and Spf1 deletion strains, i.e.: full loss of either factor, reveal only relatively limited global proteome changes compared to wild type cells. We include these additional data in the attached revision in SI Figure 1c, further supporting the well-recognized concept that membrane proteostasis networks are buffered by overlapping pathways and compensatory mechanisms.

The relatively modest phenotypes observed upon disruption of the complex are fully consistent with the well-established robustness and redundancy of ER membrane quality control. The mutations analyzed in our study weaken the interaction by approximately 70% but do not fully abolish it. Nevertheless, the mutants display clear stress-dependent defects, indicating that the interaction contributes measurably to

membrane proteostasis under challenging conditions. Importantly, the observation that both machineries retain their core activities is consistent with the conclusion that EMC and Spf1 function as independent systems that can interact without compromising their catalytic cycles.

A highly important emerging topic in the field is that membrane protein biogenesis and quality control machineries can organize into functional hubs within the ER membrane, which brings multiple machineries into proximity while preserving their independent activities. (https://pubmed.ncbi.nlm.nih.gov/36261528/, https://pubmed.ncbi.nlm.nih.gov/36261522/, https://pubmed.ncbi.nlm.nih.gov/39173640/). The structural routes for forming such assemblies while preserving the activities of the individual constituent machines had been a major unanswered question in the field. Our work provides some of the first structural insights into this principle. The EMC:Spf1 complex illustrates how two major membrane proteostasis machineries can assemble into a higher-order complex while maintaining their individual activities. Rather than forming a joint catalytic machine, this assembly may represent a membrane proteostasis hub that spatially couples insertion, extraction, and chaperoning activities. Such spatial organization will increase the efficiency and fidelity of membrane protein quality control by enabling newly inserted or misassembled membrane proteins to be rapidly assessed and, if necessary, removed locally without requiring diffusion to distant sites in the ER membrane.This notion is fully consistent with the effects we observed under stress, in which the integrative setup becomes even more relevant. In the revision, we now have placed stronger emphasis on this conceptual advance our study provides.

Specific points:

1. The abstract must state that disruption of the EMC:Spf1 supercomplex does not cause functional defects.
We revised the abstract, which now explicitly states that both proteins retain their function in the complex.

2. Given the speculative nature of the current model, Figure 6 should be removed. Alternatively, the figure could be balanced by adding a panel depicting the canonical model of EMC and Spf1 performing their respective functions independently, and moved to the Extended Data Figures.
Based on the Reviewer's suggestions, we have revised Figure 6 and now also depict the functions of the EMC and Spf1 when not in complex with each other and how they are retained in the EMC:Spf1 supercomplex. We further discuss alternative mechanisms in the figure legend. The title of this figure clearly states that this is a "possible model" and we therefore hope that this figure is not misleading but provides a visualization of our major structural findings in the context of a plausible hypothesis.

3. Fig. 5e and f: The authors should confirm by western blotting that Spf1 protein levels in yeast are comparable across mutants and under tested conditions.
As requested, the respective protein levels were assessed by western blot and one representative repliate is now shown in SI Figure 12b.

Reviewer #3 (Remarks to the Author):

The authors have appropriately addressed the reviewer's comments and concerns. The revisions have improved the manuscript, particularly in terms of clarity, figure presentation, and the discussion of structural and mechanistic interpretations. The additional explanations regarding the composite cavity, nucleotide states, and the functional implications of the observed conformational changes are especially helpful. I appreciate the authors' careful and thoughtful responses, and I believe the manuscript is now suitable for publication.

We are delighted that the reviewer appreciates our study and recommends publication in NSMB.

Reviewer #1:
Remarks to the Author:
I have re-evaluated the manuscript by Klose et al., with regards to the comments from reviewer 2 and the author responses. Overall, I am still of the opinion that the authors present compelling evidence for a novel and exciting supercomplex between EMC and Spf1 that is ready for publication. I think the additional experiments that have been included in both revisions have addressed the specific points of reviewer 2 and indicate physiological relevance of the complex. Further functional studies targeting physiological significance are in my opinion beyond the necessary scope of this current work. I would specifically comment on the major concerns as follows:

1. From the initial review: “In this regard, Spf1 could simply represent another EMC client, rather than a proofreading factor.”

The authors perform the functional assays suggested by reviewer 2 for a variety of substrates. These clearly show that Spf1 is functional within the complex and I would agree this argues against a role of the complex in Spf1 biogenesis. Structurally, Spf1 binds to a distinct site in EMC from those typically occupied by clients like CaV i.e. the lipid-filled cavity or hydrophilic groove.

2. From the first review: “this may reflect an…..experimental artifact from the use of detergent” and from the second review: “it remains unknown how stable this supercomplex is in native membranes.”

I think this is the most critical point for concerns about ‘physiological relevance’. Whilst the manuscript largely deals with a complex that has been detergent-extracted from native membranes, in my extensive experience of purifying membrane protein complexes for structural studies it is much more common a detergent destabilises a physiological complex rather than enforces a non-physiological one. The high stability and 1:1 stoichiometry of this complex (particularly when extracted at endogenous levels) together with accurate AlphaFold predictions of a novel complex, convince me this is a physiologically relevant complex. In addition, the authors show that mutations that disrupt the complex have some (albeit minor) affects on Spf1 function in native membranes and that the complex is maintained when reconstituted into a lipid environment from detergent.

3. From the second review: “the overall functional effects of EMC:Spf1 disruption appear weak” and “current evidence suggests that EMC and Spf1 can each perform their known functions well independently”

I agree with these statements but don’t understand why this is detrimental to the manuscript. As the authors explain, biogenesis and quality control pathways at the ER are often redundant and compensatory – indeed, they now show in SI Fig. 1c that knockouts of either EMC or Spf1 have modest effects on the proteome, therefore it is unsurprising the more subtle point mutations to the supercomplex have mild affects. Whilst their data show that EMC and Spf1 core functions do not require complex formation, the supercomplex could have an alternative role such as boosting the efficiency of protein quality control. To this end, the authors show that disruption of EMC:Spf1 has more pronounced effects on Gnp1 stability under stress conditions i.e. when the need for efficient protein quality control at the ER is at its greatest.

Reviewer #3:
Remarks to the Author:
After reviewing the revised manuscript and the authors' responses to Reviewer #2's concerns regarding the strength and physiological significance of the EMC association, I find the authors' arguments convincing and overall support publication of the study.

In my opinion, the biochemical and structural evidence strongly supports the existence of a specific and physiologically relevant complex. It is generally unlikely for a non-specific interaction to survive the extensive purification workflow used here, involving affinity purification followed by size-exclusion chromatography, while remaining sufficiently homogeneous for high-resolution cryo-EM structure determination. The fact that the authors were able to obtain well resolved structures of the complex argues strongly against this being a transient detergent artifact or non specific association.

Regarding the relatively modest physiological phenotypes observed upon disruption of the interaction, I agree with the authors' interpretation. Membrane protein biogenesis and quality control pathways are fundamentally important cellular processes and are therefore supported by multiple overlapping and partially redundant systems. As a result, disruption of a single interaction or pathway component often produces only subtle or context dependent phenotypes, (particularly under standard laboratory growth conditions).

Overall, I believe the revised manuscript provides compelling structural and biochemical evidence for the EMC supercomplex and offers important conceptual insight into how membrane protein biogenesis and quality control machineries may be spatially organized within the ER membrane.
