# Supplementary material for: Structural basis of an endoplasmic reticulum EMC:Spf1 insertase–dislocase complex
Source: Nat Struct Mol Biol. 2026 Oct 13;33(10):1548–60. doi: 10.1038/s41594-026-01883-8 (PMC13672079; doi:10.1038/s41594-026-01883-8)

Figure 1c

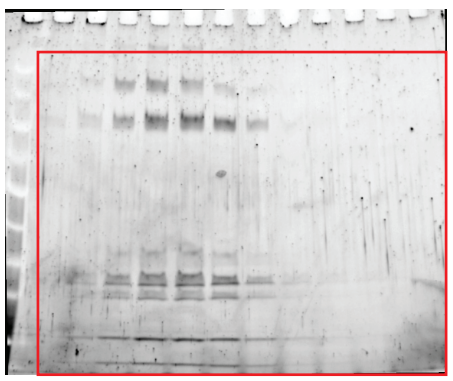

Extended Data Figure 1d

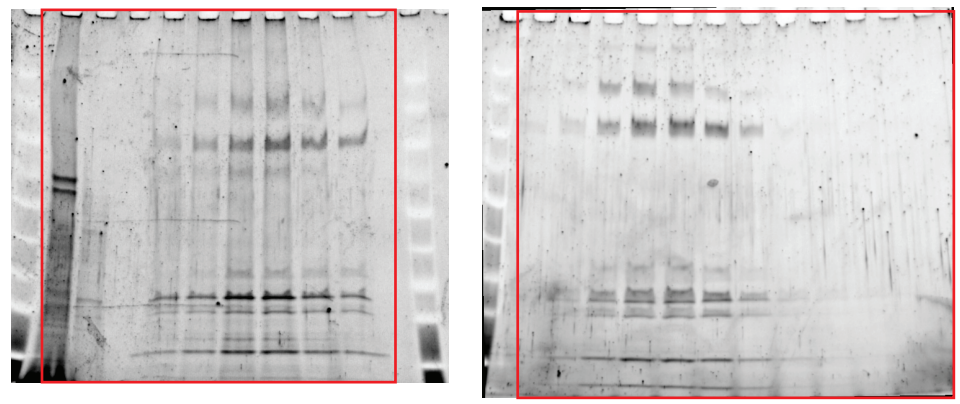

Figure 2a

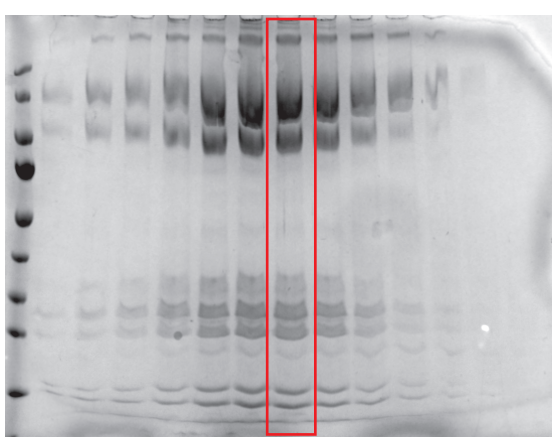

Extended Data Figure 2a

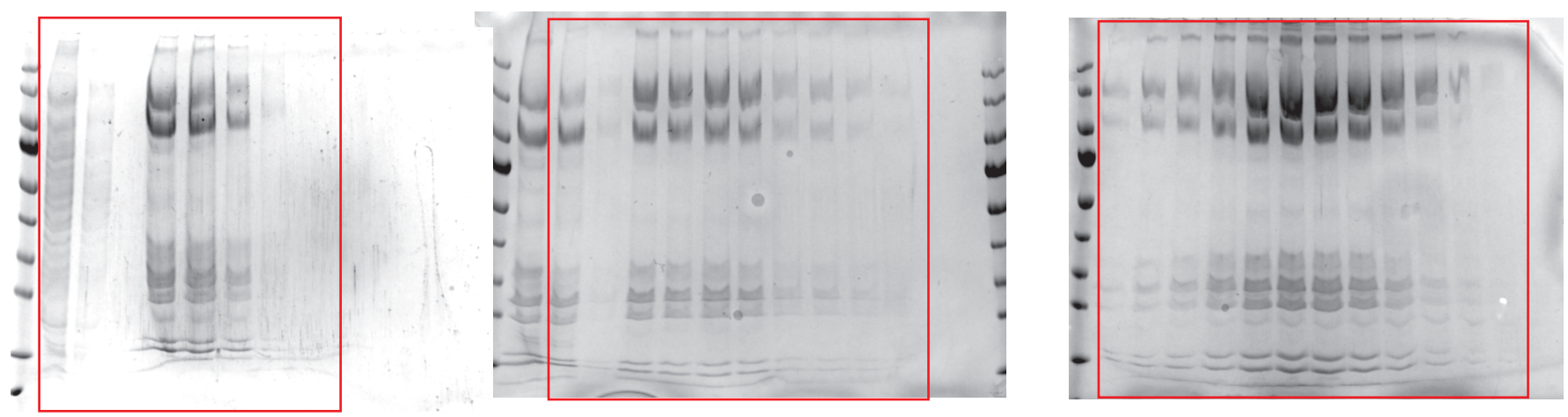

Extended Data Figure 5b

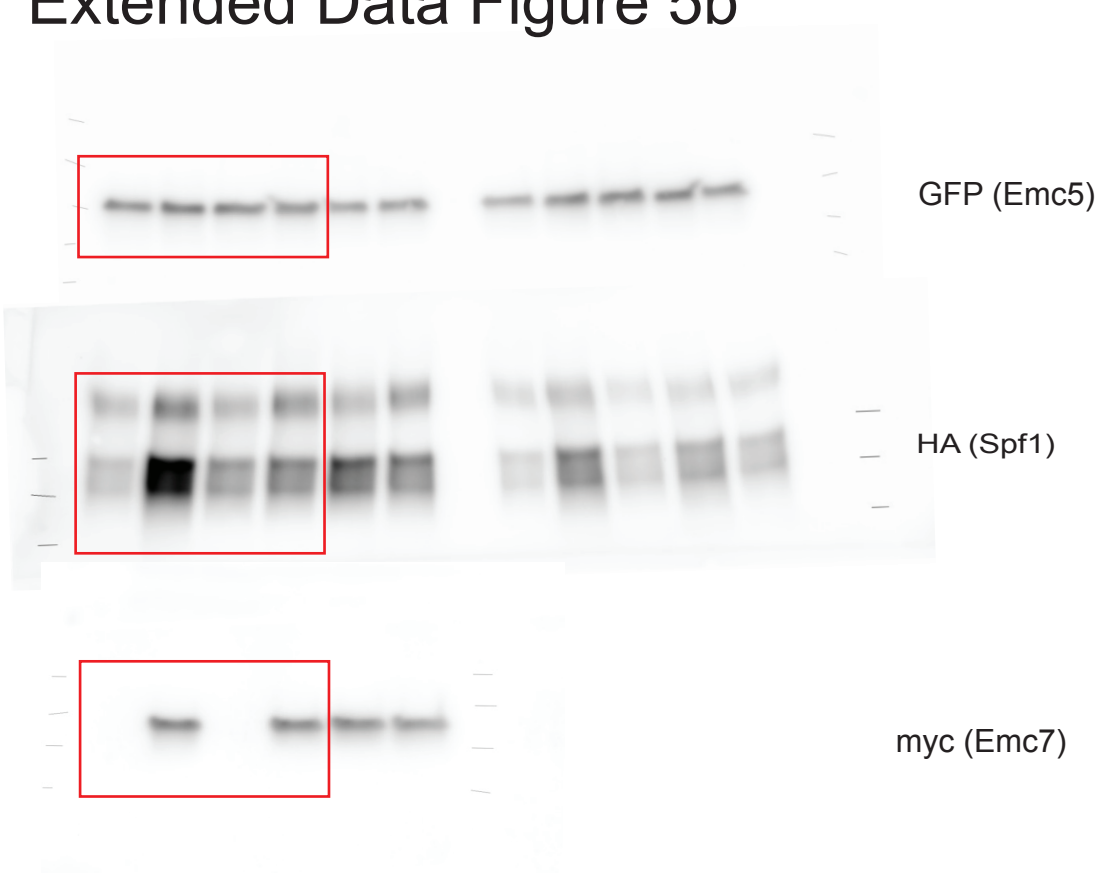

Extended Data Figure 5d

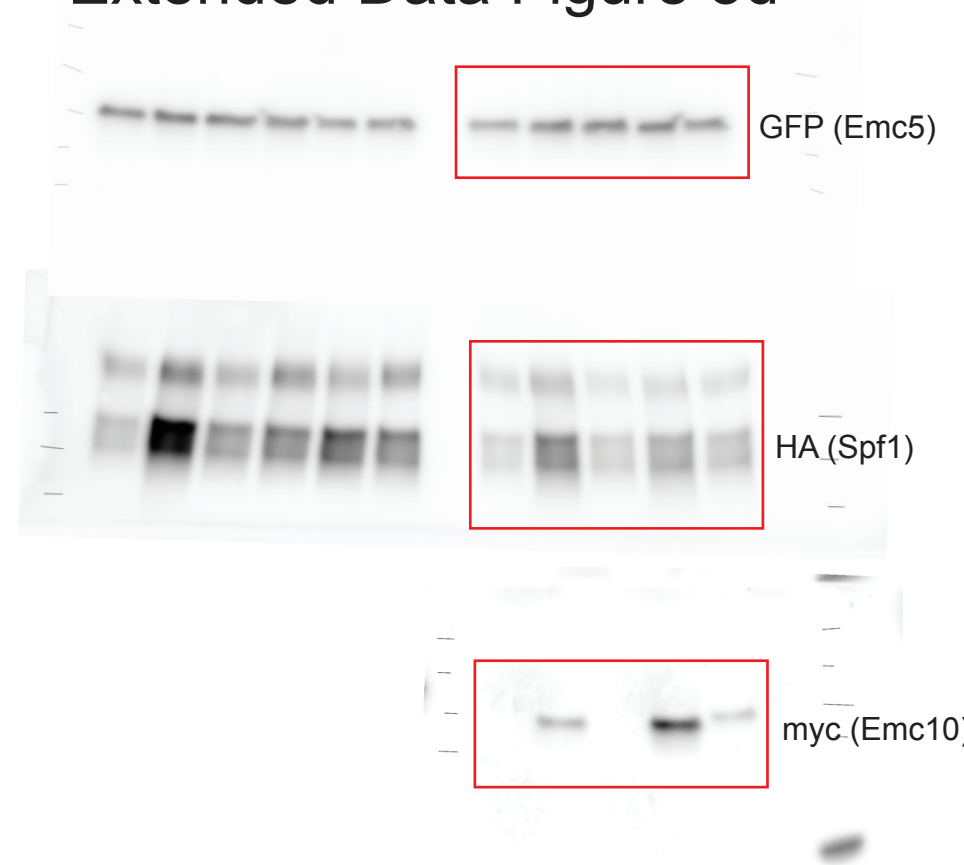

Supplement: Supplementary file 4 [file 41594_2026_1883_MOESM7_ESM.pdf]
